# Structure-based drug discovery of a corticotropin-releasing hormone receptor 1 antagonist using an X-ray free-electron laser

**DOI:** 10.1038/s12276-023-01082-1

**Published:** 2023-09-01

**Authors:** Hoyoung Kim, Taehyun Lim, Go Eun Ha, Jee-Young Lee, Jun-Woo Kim, Nienping Chang, Si Hyun Kim, Ki Hun Kim, Jaeick Lee, Yongju Cho, Byeong Wook Kim, Alva Abrahamsson, Sung Hwan Kim, Hyo-Ji Kim, Sehan Park, Sang Jae Lee, Jaehyun Park, Eunji Cheong, B. Moon Kim, Hyun-Soo Cho

**Affiliations:** 1 Department of Systems Biology, College of Life Science and Biotechnology, Yonsei University, 50 Yonsei-ro, Seoul, 03722 Republic of Korea; 2 Department of Chemistry, College of Natural Sciences, Seoul National University, Seoul, 08826 Republic of Korea; 3 Department of Biotechnology, College of Life Science and Biotechnology, Yonsei University, Seoul, 03722 Republic of Korea; 4 New Drug Development Center (NDDC), Daegu Gyeongbuk Medical Innovation Foundation (K-Medi hub), 80 Chumbok-ro, Dong-gu, Daegu, 41061 Korea; 5 Doping Control Center, Korea Institute of Science and Technology, Seoul, 02792 Republic of Korea; 6 Department of Chemistry, Sogang University, Seoul, 04107 Republic of Korea; 7 Pohang Accelerator Laboratory, POSTECH, Pohang, 37673 Republic of Korea

**Keywords:** Protein-protein interaction networks, Drug delivery

## Abstract

Thus far, attempts to develop drugs that target corticotropin-releasing hormone receptor 1 (CRF_1_R), a drug target in stress-related therapy, have been unsuccessful. Studies have focused on using high-resolution G protein-coupled receptor (GPCR) structures to develop drugs. X-ray free-electron lasers (XFELs), which prevent radiation damage and provide access to high-resolution compositions, have helped accelerate GPCR structural studies. We elucidated the crystal structure of CRF_1_R complexed with a BMK-I-152 antagonist at 2.75 Å using fixed-target serial femtosecond crystallography. The results revealed that two unique hydrogen bonds are present in the hydrogen bond network, the stalk region forms an alpha helix and the hydrophobic network contains an antagonist binding site. We then developed two antagonists—BMK-C203 and BMK-C205—and determined the CRF_1_R/BMK-C203 and CRF_1_R/BMK-C205 complex structures at 2.6 and 2.2 Å, respectively. BMK-C205 exerted significant antidepressant effects in mice and, thus, may be utilized to effectively identify structure-based drugs against CRF_1_R.

## Drug discovery: Hormone receptor structure helps search for new antidepressants

Structural studies of complexes of a receptor protein for corticotropin-releasing hormone and specific small molecules could guide the development of new antidepressants and drugs for stress-related diseases. The activity of the corticotropin-releasing hormone receptor 1 protein has been associated with many human diseases, including psychiatric disorders, diabetes, cancer, osteoporosis, cardiovascular conditions and neurodegeneration. Hoyoung Kim at Yonsei University in Seoul, South Korea, utilized X-ray free electron laser technology, a method that minimizes sample damage, to determine the protein complexes. Through their study, they unveiled subtle, previously overlooked structural characteristics at two crucial sites. Leveraging these insights, they developed multiple ‘antagonist’ compounds that disrupt the protein’s normal function. One of these antagonists demonstrated significant antidepressant effects in mouse models, sparking hope for potential similar benefits in humans.

## Introduction

Human class B G protein-coupled receptors (GPCRs) are important drug targets in many human diseases, including diabetes, cancer, osteoporosis, cardiovascular disease, neurodegeneration, and psychiatric disorders. Agonists or antagonists that modulate GPCR activity are potential drug candidates for efficient therapeutic disease interventions^1^. Corticotropin-releasing hormone receptor 1 (CRF_1_R), a class B secretin in the G protein-coupled receptor family, contains receptors involved in critical peptide hormone regulation, such as corticotropin-releasing factor (CRF)- and urocortin (UCN1)-activated CRF_1_R^2^. Previous studies have indicated that CRF mRNA and CRF are widely distributed in the central nervous system (CNS) and are enriched in the paraventricular nucleus (PVN) of the hypothalamus, brain stem, amygdala, hippocampus, and neocortex^2^. CRF_1_R regulates behavioral, endocrinal, immune, and autonomic responses to stress in humans and animals^3^. The autonomic nervous system and the hypothalamus–pituitary–adrenal (HPA) axis are activated in response to physical or psychological stress. The release of CRF by the PVN activates CRF receptors in the anterior lobe of the pituitary gland, leading to adrenocorticotropic hormone (ACTH) secretion. ACTH stimulates the adrenal cortex, producing and releasing glucocorticoid hormones (cortisol in humans and corticosterone (CORT) in mice), which act as stress hormones. Many studies have shown that HPA axis hyperactivity, which leads to increased basal levels of glucocorticoid hormones^4,5^ and alterations in the CRF system^6^, is associated with depressive disorders. In some patients with depression, hypothalamic PVN displays increased CRF concentrations in the cerebrospinal fluid^7^, increased CRF immunoreactivity^8^, and CRF mRNA overexpression^9^. Therefore, CRF_1_R antagonists, which downregulate HPA axis activity, show great potential as antidepressants^7^. Depression, a major psychiatric disorder, occurs in 4.7% of adults over the age of 18^10^. Selective serotonin reuptake inhibitors (SSRIs) and serotonin-noradrenaline reuptake inhibitors (SNRIs) are the most frequently used antidepressants. However, in multiple trials, ~30% of patients did not respond to SSRIs or SNRIs^11^. Therefore, an increasing demand for a new class of antidepressants has materialized^12^.

In 2012, the structure of the transmembrane domain (TMD) of CRF_1_R was determined in its inactive state as a complex bound to a small-molecule antagonist, CP376395. However, the structure of its extracellular domain (ECD) was not established^13^. The inactive CRF_1_R structure, ascertained in 2017, confirmed that the flexibility of extracellular loop 3 (ECL3) in opening and closing the CRF_1_R ECD was important^14^. In 2020, the CRF_1_R structure in its activated state was determined via cryogenic electron microscopy (Cryo-EM). Elucidation of these structures enabled us to study conformational changes in the TMD during CRF_1_R activation^15,16^. However, the complete composition of CRF_1_R in its inactive state, including that of its ECD, remains unclear, and determining an exact activation mechanism is difficult. In particular, these previous studies did not identify the activation of the CRF_1_R stalk region, which links its ECD to its TMD and is vital in the activation mechanism^17^. Moreover, all CRF_1_R-targeting antagonists have been excluded from clinical processes, preventing CRF_1_R-targeting drugs from being developed^12^.

We used an X-ray free-electron laser (XFEL) to determine the CRF_1_R structure and the composition of BMK-I-152, an antagonist known for its high binding affinity to CRF_1_R. BMK-I-152 is an allosteric antagonist located in the hydrophobic network of the CRF_1_R structure^13^. Although we used the same CRF_1_R construct, our structure exhibited less activity than that of previous structures. We analyzed the stalk region of this inactive state via structural comparisons. Previous studies have indicated that BMK-I-152 bound CRF_1_R with a 0.35 nM Ki value^18^ but showed sparse metabolic stability with a half-life of <30 min in a liver microsomal stability assay. This indicated that BMK-I-152 might be ineffective in animal models or as a treatment. We developed new compounds to solve this issue by simulating structure-based docking and selected BMK-C203 and BMK-C205 based on calcium mobilization, cytochrome P450 (CYP) inhibition, and microsomal stability assays. We subjected mouse CRF_1_R antagonist-treated models to tail suspension tests (TSTs), a depressive behavior test, to verify the antidepressant effects of BMK-C203, BMK-C205, and antalarmin (a conventional CRF_1_R antagonist). Additionally, we measured the ACTH and CORT serum levels to confirm that HPA axis activation had decreased. The results indicated that only BMK-C205 effectively reduced in vivo depression-like behavior and HPA axis activation. These results may help researchers develop new antidepressant drugs based on high-resolution crystal structures.

## Methods

### CRF_1_R expression and purification

Crystal-generating CRF_1_R construction was based on a previously published method to construct CRF_1_R. T4L was fused into the second intracellular loop of CRF_1_R (T220–L222) with truncations at the N-terminal 1–103 residues and C termini residue 374–end and 12 thermostabilization mutations^13^. The modified CRF_1_R-T4L protein was expressed in *Spodoptera frugiperda* (sf9) cells using ESF921 cell culture media (Expression Systems) and the Bac-to-Bac baculovirus expression system (Invitrogen) for 60 h. The insect cells were disrupted via repeated washing and centrifugation with a hypotonic buffer (10 mM HEPES, 10 mM MgCl_2_, and 20 mM KCl; pH 7.5) containing protease inhibitors (once; 500 μM AEBSF, 1 μM E-64, 1 μM leupeptin, and 150 nM aprotinin) and a high-osmotic buffer (three times; 1.0 M NaCl, 10 mM HEPES (pH 7.5), 10 mM MgCl_2_, and 20 mM KCl). Purified membranes were resuspended in a buffer containing 50 mM Tris–HCl (pH 8.0), 500 mM NaCl, and protease inhibitors (500 μM AEBSF, 1 μM E-64, 1 μM leupeptin, and 150 nM aprotinin). Additionally, 25 µM CRF_1_R antagonist (BMK-I-152 or other antagonists), 2 mg/mL iodoacetamide (Sigma), 1% (w/v) n-dodecyl-β-d-maltopyranoside (DDM) (Anatrace), and 0.2% (w/v) cholesteryl hemisuccinate (CHS) (Anatrace) were added for 2 h at 4 °C for solubilization. Insoluble materials were removed via centrifugation at 150,000×*g* for 30 min, followed by incubation with TALON IMAC resin (Clontech) overnight at 4 °C. Next, the resin was washed with 20 column volumes (CVs) of wash buffer (50 mM Tris–HCl (pH 8.0), 500 mM NaCl, 0.05% (w/v) DDM, 0.01% (w/v) CHS, 20 mM imidazole, and 5 µM CRF_1_R antagonist (BMK-I-152 or other antagonists)). The protein was then eluted in 5–6 CVs of elution buffer (50 mM Tris–HCl (pH 8.0), 500 mM NaCl, 0.05% (w/v) DDM, 0.01% (w/v) CHS, 200 mM imidazole, and 5 µM CRF_1_R antagonist (BMK-I-152 or other antagonists)). The eluted CRF_1_R was purified via gel filtration in 20 mM Tris–HCl (pH 8.0), 150 mM NaCl, 0.05% (w/v) DDM, 0.01% (w/v) CHS, and 1 µM CRF_1_R antagonist (BMK-I-152 or other antagonists). Finally, the collected CRF_1_R was concentrated to 20 mg/mL with a 100 kDa molecular mass cutoff Vivaspin (GE Healthcare).

### LCP crystallization

CRF_1_R/antagonist complexes were reconstituted into the LCP by mixing proteins and a monoolein and cholesterol mixture at a ratio of 40%:54%:6% using the twin-syringe method^19^. Crystallization was performed on 96-well glass sandwich plates (LPS solution) using a Mosquito machine (TTP LabTech), and 60 nL of protein-laden LCP and 800 nL of precipitant solution were dispensed per well. The plates were incubated at 20 °C. Crystals, which reached their full size after ~2 weeks, were obtained using 100 mM Na-citrate (pH 4.5), 50 mM NaCl, and 26–28% PEG400 (Supplementary Fig. 1a). The crystals were harvested using MicroMounts (MiTeGen) directly from the LCP and flash-frozen in liquid nitrogen to collect diffraction data at the synchrotron radiation beamline. Microcrystals of CRF_1_R/antagonists for XFEL data collection were prepared by injecting 5 μL LCP mixture aliquots into a 100 μL Hamilton syringe filled with 70 μL precipitant solution (100 mM Na-citrate (pH 4.5), 50 mM NaCl, and 26–28% PEG400)^20^. As a result, crystals grew to an average size of 20 μm within 1–2 weeks at 20 °C (Supplementary Fig. 1b).

### Obtaining crystal unit cell information using synchrotron radiation

X-ray diffraction data of CRF_1_R/antagonist crystals were collected at the Pohang Accelerator Laboratory (PAL) beamline 11C, Pohang, Korea, using a PILATUS3 6 M Detector. The crystals were exposed to 1 s and 1° oscillations per frame to collect ~30 diffraction data points. The diffraction data were indexed by XDS^21^, and crystal unit cell information (*a* = 95.66 Å, *b* = 70.65 Å, *c* = 86.75 Å, *α* = 90°, *β* = 97.82°, and *γ* = 90°) was obtained to perform serial femtosecond crystallography (SFX).

### Diffraction data collection using an XFEL

SFX data collection was performed using an NCI instrument at PAL-XFEL^22,23^ and the fixed target method^24^. The sample holder and lid were made to size using a polyvinyl chloride (PVC) frame and ketone film in a double-sided form for use in the fixed target method. They were made sequentially using double-sided tape, a PVC frame, and ketone film. The precipitant solution was removed using a Hamilton syringe, and the crystal was sprayed on the sample holder and covered with the sample holder lid. The ketone film part of the sample holder was gently pressed and evenly spread below 0.5 mm (Supplementary Fig. 1c). An X-ray energy of 9.705 keV with a photon flux of ~5 × 10^11^ photons per pulse was used for 20 fs. Data were collected at room temperature and recorded using an MX225-HS detector (Rayonix, LLC, Evanston, IL, USA) with a 4 × 4 binning mode (pixel size: 156 μm × 156 μm). The distance between the detector and sample holder was 150 μm. The sample holder containing the crystals was scanned at 50-μm intervals from left to right. The bottom was scanned at 50 μm. The next scan was performed from right to left at 50-μm intervals. The bottom was scanned at 50 μm with a scan range of 22 mm × 22 mm.

### Data processing and structure determination

Collected images were filtered using Cheetah^25^, and filtered images were indexed using crystFEL^26^. Cell parameter values obtained via synchrotron radiation diffraction were used, while MOSflm^27^, XDS^21^, and dirax^28^ were used for data indexing. Indexing command defaults were applied to all others except int-radius = 3,5,6. Phasing of CRF_1_R was obtained by molecule replacement via Phaser-MR in PHENIX with CRF_1_R (PDB code: 4K5Y)^13^. The refinements were Coot^29^ and Phenix.refinement in PHENIX^30^. Supplementary Table 1 shows the structural information.

### Calcium mobilization assay

HEK-Gα15 cells (Eurofins Scientific) transfected with CRF_1_R were plated in 96-well plates at a density of 50,000 cells/100 μL per well and incubated overnight. The next day, the cells were incubated with a Calcium 6 reagent (Molecular Devices) containing 0.5 mM probenecid (Sigma) at 37 °C for 1 h before adding compounds or dimethyl sulfoxide DMSO (positive and negative control; final concentration 0.1%). After incubation at 37 °C for 1 h, 50 μL urocortin (final concentration 2 nM) (Tocris) was dispensed into the well with FLIPR-tetra (Molecular Devices). Urocortin-free buffer was used as a positive control (100% inhibition). Intracellular calcium changes were recorded at excitation and emission wavelengths of 470–495 and 515–575 nm, respectively. Relative fluorescence units (RFU) were calculated as the fluorescence signal maximum minus the signal minimum for 30 s after addition. The graphs and IC_50_ concentrations were calculated using GraphPad Prism 6 software.

### Chemical synthesis

The detailed synthesis and analytical characterization of all the CRF_1_R antagonist compounds Supplementary Data 1.

### CYP inhibition assay

Phenacetin O-demethylase, diclofenac 4-hydroxylase, S-mephenytoin 4-hydroxylase, dextromethorphan O-demethylase, and midazolam 1’-hydroxylase activities were determined as probe activities for CYP1A2, CYP2C9, CYP2C19, CYP2D6, and CYP3A4, respectively, using cocktail incubation. The cocktails were as follows: phenacetin 50 μM (CYP1A2), diclofenac 10 μM (CYP2C9), S-mephenytoin 100 μM (CYP2C19), dextromethorphan 5 μM (CYP2D6), and 2.5 μM midazolam (CYP3A4). The cocktail incubation mixtures consisted of 0.25 mg/mL human liver microsomes, 0.1 M phosphate buffer (pH 7.4), an NADPH regeneration system, and inhibitors (CRF_1_R antagonist compounds, 10 µM) in a total volume of 100 μL. The final volume of the organic solvents in the incubation mixture was 1% (v/v). After a 5 min preincubation at 37 °C, the reactions were initiated by adding the NADPH regeneration system at 37 °C for 15 min and were terminated by adding 50 μL of ice-cold acetonitrile with the internal standard (terfenadine). All incubations were performed in triplicate, and the mean values were used for analysis. The samples were then centrifuged at 15,000 rpm for 5 min at 4 °C. Aliquots of the supernatant were analyzed by liquid chromatography–tandem mass spectrometry (LC‒MS/MS) for the analysis of each metabolite^31^. The CYP-mediated activities in the presence of inhibitors were expressed as percentages of the corresponding control values.

### Microsomal stability assay

The incubation mixtures consisted of 0.5 mg/mL human (Corning, #452117) and rat liver microsomes (Corning, #452701), 0.1 M phosphate buffer (pH 7.4), NADPH regeneration system, and substrates (CRF_1_R antagonist compounds, 1 µM) in a total volume of 100 μL. After a 5 min preincubation at 37 °C, the reactions were initiated by adding the NADPH regeneration system at 37 °C for 30 min and were terminated by adding 40 μL of ice-cold acetonitrile with the internal standard (chlorpropamide). The final volume of the organic solvents in the incubation mixture was 1% (v/v). All incubations were performed in triplicate, and the mean values were used for analysis. Precipitated proteins were removed by centrifugation at 15,000 rpm for 5 min at 4 °C. Aliquots of the supernatant were analyzed by liquid chromatography–tandem mass spectrometry coupled with electrospray ionization (LC‒MS/MS) for the analysis of CRF_1_R antagonist compounds. LC‒MS/MS was performed on a TSQ Vantage (Thermo, USA) coupled with a Nexera XR LC system (Shimadzu, Japan)^32^. The separation was performed on a Kinetex C18 column (2.1 × 100 mm, 2.6 µm, Phenomenex, USA) using a mobile phase of 0.1% formic acid and acetonitrile containing 0.1% formic acid. A gradient program was used for elution at a flow rate of 0.3 mL/min. The data were acquired using multiple-reaction monitoring (MRM) in positive mode. Detection of the ions was performed by monitoring the m/z transition of 494.1 → 336.1 for BMK-I-152, 598.1 → 233.2 for BMK-C203, and 488.1 → 434.1 for BMK-C205.

### Bioluminescence resonance energy transfer assays (BRET)

To measure antagonism, our compounds in CRF_1_R or CRF_2_R (corticotropin-releasing hormone receptor 2) Freestyle^TM^ 293F (Thermo Fisher, R79007) cells were cotransfected in a 1:1 ratio with human CRF_1_R or CRF_2_R containing C-terminal Renilla luciferase (RLuc8) and Venus-tagged N-terminal MiniG_s_^33^. After at least 16 hours, transfected cells were plated in poly-lysine-coated 96-well white clear bottom cell culture plates in plating media (DMEM + 1% dialyzed FBS) at a density of 40,000–50,000 cells in 200 μL per well and incubated overnight. The next day, the medium was decanted, and the cells were washed twice with 60 μL of drug buffer (1 × HBSS, 20 mM HEPES, 0.1% BSA). Before the measurements were performed, 30 μL of the RLuc substrate, coelenterazine h (Promega, 5 μM final concentration), and 30 μL of our compounds (100 μM–10 pM final concentration) in drug buffer were added per well and incubated for an additional 20 min at RT for compound diffusion. Then, 30 μL of UCN1 (1 μM final concentration) per well and plates were immediately read for luminescence at 480 nm and fluorescent eYFP emission at 530 nm for 1 s per well using CLARIOstar® Plus. The ratio of eYFP/RLuc was calculated per well in GraphPad Prism 7.0.

### Docking simulation

Docking simulations, which were based on the X-ray CRF_1_R/BMK-I-152 complex structure, were performed to determine the binding model of the analogs of BMK-I-152 and CRF_1_R. The binding sites of the three analogs (Supplementary Fig. 8 1n, i, and f) for CRF_1_R were defined, and the receptor‒ligand interactions were considered (Supplementary Fig. 8c–e). Docking simulation was performed using a Glide module, and GlideScore was used to determine the priority of the docking model. The CRF_1_R ligand-binding site was defined using a receptor grid-based method, and XP (extra precision) modes were used to minimize the docking model. All simulations were performed using MAESTRO 11.5 (Schrödinger LLC, NY, USA) on a Linux system.

### PK studies in rats

CRF_1_R antagonist PKs were studied in naïve male SD rats following a single i.v. bolus injection or p.o. gavage. The i.v. injections used Antalarmin, BMK-C203, or BMK-C205 at doses of 5 mg/kg and BMK-I-152 at a dose of 1 mg/kg. The antagonist’s doses in the p.o. injection was 10 mg/kg. Three male SD rats weighing between 220 and 260 g were used to evaluate CRF_1_R antagonist PKs. The vehicle used for all dose groups was phenol: TPGS: PEG400 (70%:20%:10%). Blood samples were collected from all treatment groups at 0 h (predose), 0.083, 0.25, 0.5, 1, 2, 4, 6, 8, and 24 h after dosing. Antalarmin, BMK-I-152, BMK-C203, and BMK-C205 concentrations in the plasma samples were determined via LC‒MS/MS (LLOQ was 1 ng/mL for CRF_1_R antagonists). PK parameter estimates were determined using the serial time-course profiles for plasma concentrations of three male rats per group via noncompartmental analysis using commercially available software (Phoenix WinNonlin, version 8.1, Pharsight Corporation, Mountain View, CA).

### Behavior testing

C57BL/6J mice (male, 9–10 weeks old) were used for the behavioral tests. All mice were maintained under a 12:12-h light–dark cycle (lights on at 7:00 A.M.) with access to food and water ad libitum. Animal care and handling were performed according to guidelines by the Institutional Animal Care and Use Committee of Yonsei University (Seoul, Korea). Antalarmin, BMK-C203, and BMK-C205 were dissolved in peceol: TPGS: PEG400 (vehicle) (70%:20%:10%, respectively) and administered orally at a dose of 30 mg/kg for 3 days at 24-h intervals. All behavioral tests were conducted 2 h after the third drug was administered during light periods of the circadian cycle. TSTs were performed to verify the effect of CRF_1_R antagonists on depressive-like behavior. Each mouse was suspended by its tail on a bar 30 cm above the ground for 6 min using adhesive tape. Their movements were recorded by a camera and analyzed using the same parameters via EthoVision XT 8.5 software (Noldus Technologies). Immobility times during the last 4 min of the task were measured. Each mouse was placed in an open field (40 cm × 40 cm × 40 cm chamber) for 1 h to conduct an open field test (OFT) and test locomotion. Their locomotion was recorded and analyzed using EthoVision XT 8.5.

### Measuring serum CORT and ACTH levels

The mice were anesthetized via intraperitoneal injections of 2,2,2-tribromoethanol (300 mg/kg, Sigma-Aldrich) immediately after the TST and before blood from the heart was sampled. Blood samples were collected in plastic tubes and incubated at room temperature for at least 2 h. The sera were extracted after centrifugation at 13,000×*g* for 10 min at 4 °C. CORT levels were determined via LC‒MS analysis. Serum (10 µL) was mixed with 20 μL of an internal standard solution (d_4_-cortisol) to prepare the samples. Subsequently, 100 μL of acetonitrile was added, and the mixture was centrifuged at 16,000×*g* for 10 min for protein precipitation. The supernatants (60 μL) were transferred into an autosampler vial, and 30 μL of water was added for efficient separation via high-performance liquid chromatography (HPLC). LC‒MS was performed with a triple quadrupole mass spectrometer coupled with HPLC using a C18 reversed-phase column. CORT levels were determined using a calibration curve constructed using CORT-spiked mouse serum. Serum samples (20 μL) were analyzed using a commercially available ELISA kit for ACTH (ab263880, Abcam) to measure ACTH serum levels.

### In vitro BBB permeability assay

Human cerebral microvascular endothelial cells (hCMECs/D3, SCC066; Sigma-Aldrich) were used to assess the permeability of CRF_1_R antagonists across the BBB. hCMECs were seeded on Falcon^TM^ cell culture inserts (08-770, Fisher Scientific) with 0.4 μm pore sizes precoated with collagen type I from rat tails (354236, Corning) at a density of 50,000 cells/mL in endothelial cell growth medium-2 (EGM-2, CC-3162; Lonza). The medium was changed with EGM-2 every three to four days for proliferation and with EGM-2 containing 2.5% human serum (SLBX6020, Sigma-Aldrich) for differentiation. Cell culture inserts were transferred into 24-well plates containing 700 μL of EGM-2 in each well (basolateral compartment) for the permeability assay. The medium in the inserts (apical compartment) was then replaced with 300 μL EGM-2 containing 10 μM BMK-C203 or BMK-C205. Apical and basolateral compartments were collected after a 12-h incubation at 37 °C, and drug concentrations were measured using HPLC. For sample preparation, 50 μL of the sample was mixed with 10 μL of an internal standard solution (d4-cortisol). Subsequently, 50 μL of acetonitrile was added, and the mixture was centrifuged at 16,000×*g* for 10 min for protein precipitation. The supernatants were used for LC‒MS analysis with a triple quadrupole mass spectrometer coupled with HPLC using a C18 reversed-phase column. The concentration of the antagonists was determined using a calibration curve. The permeability coefficient (*P*) was calculated according to Pardridge et al.^34^. The permeability-surface area product (PS, in cm^3^/s) was divided by the filter surface area *A* (in cm^2^) using Eq. (1) as follows:

1 $$P\left[{\rm {{cm}/s}}\right]=\frac{{V}_{{\rm{d}}}\times \triangle {M}_{\rm {{a}}}}{A\times {M}_{\rm {{b}}}\times \triangle {t}}$$

where *V*_d_ is the volume in the donor compartment in cm^3^, Δ*M*_a_ is the total amount of the compound in the apical compartment after *t* seconds, *M*_d_ is the donor amount, Δ*t* is the incubation time, and *A* is the filter area in cm^2^.

## Results

### Overall structure of CRF_1_R with BMK-I-152

**Fig. 1:**
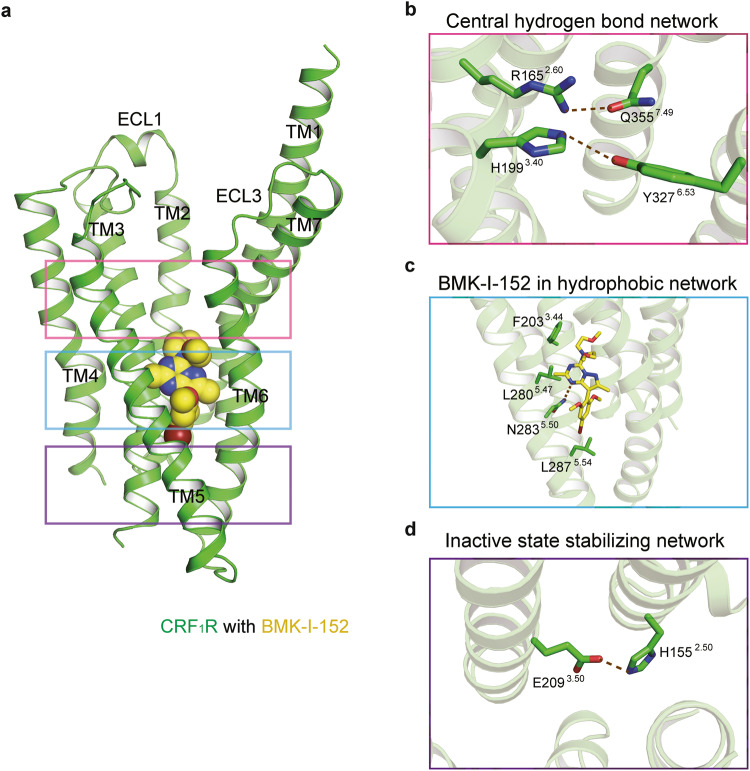
Overall structure of CRF_1_R with BMK-I-152. **a** Cartoon representation of the CRF1R and BMK-I-152 complex. CRF_1_R (residues 104–368) and BMK-I-152 are colored green and yellow, respectively. CRF1R utilizes the following major interaction networks: the **b** central hydrogen bond network, **c** hydrophobic network, and **d** inactive state stabilizing network. BMK-I-152 is in the hydrophobic network.

Our human CRF_1_R (hCRF_1_R) Ca^2+^ antagonism assays showed that BMK-I-152 has a half maximal inhibitory concentration (IC_50_) value ~55-fold and 12-fold lower than that of CP376395^13,35^ and Antalarmin^36^, respectively (Supplementary Fig. 2). We used the same CRF_1_R/CP376395^13^ complex construct, including the T4 lysozyme (T4L) insertion in intracellular loop 2 (ICL2), N- and C-terminal deletion, and 12 thermostabilization mutations, to obtain structural insight into the high-affinity ligand binding mode of CRF_1_R. We observed the crystal structure of the CRF_1_R/BMK-I-152 complex at a resolution of 2.75 Å using an XFEL (Supplementary Table 1). We applied the Ballesteros–Weinstein numbering method to explain conserved motifs in the transmembrane domains of CRF_1_R^37^. Our results showed typical class B GPCR structural inactive state motifs, including the central hydrogen bond, hydrophobic, and inactive state stabilizing networks^38^(Fig. 1a). We identified two hydrogen bonds (R165^2.60^–Q335^7.49^ and H199^3.40^–Y327^6.53^) in the central hydrogen-bond network (Fig. 1b). Among these, only the R165^2.60^–Q335^7.49^ bond was conserved in other class B GPCR structures, such as the glucagon receptor (GCGR) (PDB code: 5XEZ)^39^, parathyroid hormone receptor 1 (PDB code: 6FJ3)^40^, and glucagon-like peptide 1 (GLP1) receptor (PDB code: 5VEW)^41^ (Supplementary Fig. 3c). Most class B GPCRs highly conserved the R165^2.60^ and Q335^7.49^ residues (Supplementary Fig. 3b)^42^. However, the H199^3.40^–Y327^6.53^ hydrogen bond was not identified in GCGR, parathyroid hormone receptor 1, or GLP1 receptor (Supplementary Fig. 3c). The H199^3.40^–Y327^6.53^ hydrogen bond can only be found in CRF_1_R because other class B GPCR members do not conserve these residues (Supplementary Fig. 3b). The two hydrogen bonds are missing in the CRF_1_R active state structure (PDB code: 6P9X)^15^. Therefore, we concluded that these two hydrogen bonds break when the inactive state converts to the active state (Supplementary Fig. 3a). Our structure was determined while BMK-I-152 remained bound to the hydrophobic network and the allosteric antagonist binding pocket (Fig. 1c), which also binds in the same position as the previously determined CRF_1_R/CP376395 complex (Supplementary Fig. 4a). Although three major polar interactions (H^8.47^–E^8.49^–R^6.37^, H^2.50^–E^3.50^, and Y^7.57^–T^6.42^) stabilize inactive class B GPCRs^38^, we only observed one of them (E209^3.50^–H155^2.50^) in our structure (Fig. 1d).

### Comparison of structure to other CRF_1_R structures

Our structure exhibits several features that differ from those of previously determined structures. The first (PDB code: 4K5Y)^13^ and second (PDB code: 4Z9G)^14^ previously established structures comprise orthorhombic and hexagonal crystals, respectively, with three CRF_1_R structures per unit cell. However, our structure exhibits a monoclinic crystal structure and one CRF_1_R structure per unit cell (Supplementary Fig. 5a, b). The TM6-ECL3-TM7 portions in the first and second structures differed slightly for each chain. This part is missing in some structure chains, and TM7 of 4K5Y chain A is tilted ~15° outward (Supplementary Fig. 5c). However, unlike the previously determined structures, the current composition generated a clearer electronic density TM6-ECL3-TM7 map than that of the other CRF_1_R structures and showed a hydrogen bond at R165^2.60^–Q335^7.49^. This hydrogen bond helps stabilize the region. In addition, we found that the three interaction network portions of our structure’s TMD had lower *B*-factor values, indicating structural stability compared to other sections of the structure (Supplementary Fig. 5d). Our structure shows two inactive hydrogen bond motifs (Supplementary Fig. 5e), whereas the previously determined structures have only one inactive hydrogen bond motif because the other hydrogen bond in the central hydrogen bond network is missing (Supplementary Fig. 5f). However, the previously determined compositions contain a weak hydrogen bond between R165^2.60^ and Y195^3.36^, similar to that in the CRF_1_R activation arrangement (Supplementary Fig. 5g). These findings demonstrated that our structure forms a more stable inactive state than the other CRF_1_R structures.

### Dynamic secondary structure of the CRF_1_R stalk region

Recently, the inactive and active states of the structure of class B GPCRs were revealed using crystallography and cryo-EM, respectively^43^. Structural analyses suggested that class B GPCRs contain a stalk region that connects their ECD to their TMD and that this region changes depending on activation status. The following types of inactive state stalks are known thus far: the beta-sheet type (shown in the GCGR1 structure^39^) and the loop type (shown in the GLP1 receptor structure^44^). Our CRF_1_R structure also included the stalk region. The shape of the stalk region in our structure may be affected by crystal packing. However, the stalk region in our structure adopts an alpha-helix form. Structural alignment with the ECD structure of ligand-free CRF_1_R (PDB code: 3EHS)^45^ confirmed that the stalk overlapped well with the over-one-turn alpha helix region (Supplementary Fig. 6a). However, when structural alignment was performed with the CRF_1_R ECD and a bound ligand (PDB code: 3EHT)^45^, the CRF_1_R ligand and stalk region in the alpha-helix form appeared to collide (Supplementary Fig. 6b). This indicated that the inactive CRF_1_R stalk region has an alpha-helical form that changes when CRF_1_R binds to an endogenous ligand (Supplementary Fig. 6c).

### CRF_1_R structure and BMK-I-152 binding mode

**Fig. 2:**
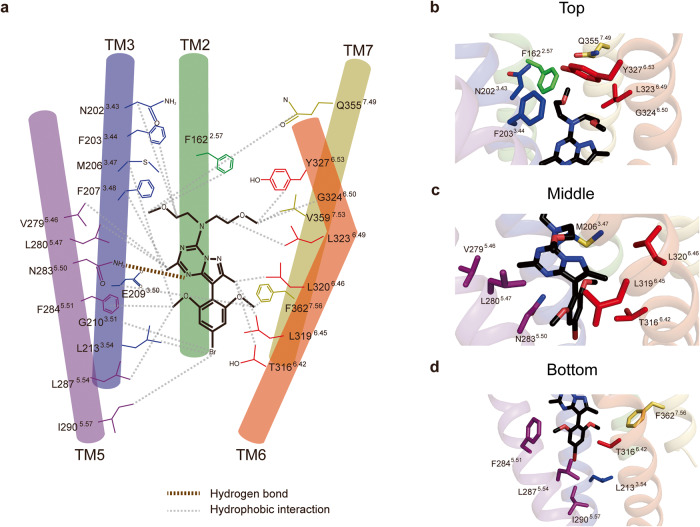
CRF_1_R structure and BMK-I-152 binding mode. **a** Schematic of the binding mode with hydrogen bonds and hydrophobic interactions shown as brown and gray dotted lines, respectively. BMK-I-152 can be divided into the following major groups. **b** An exocyclic alkylamino group, **c** pyrazolo[1,5-α][1,3,5]triazine, and **d** an aryloxy moiety. The binding between each group and the CRF1R residue is presented as a cartoon representation.

BMK-I-152, which is located in the hydrophobic network of the allosteric antagonist-binding site, binds via various hydrophobic interactions and a highly conserved Asn283^5.50^ hydrogen bond. BMK-I-152 may be divided into top, middle, and bottom regions by considering the extracellular region as the top standard (Fig. 2a). The top portion, the exocyclic alkyl amino group, forms hydrophobic interactions with Phe162^2.57^, Asn202^3.43^, Phe203^3.44^, Gly324^6.50^, Tyr327^6.53^, Leu323^6.49^, Gln355^7.49^, and Val359^7.53^ (Fig. 2b). The middle pyrazolo[1,5-α][1,3,5]triazine bicyclic core forms a hydrogen bond with the highly conserved Asn283^5.50^ and hydrophobically interacts with Phe207^3.48^, Val279^5.46^, Leu280^5.47^, Thr316^6.42^, L319^6.45^, and L320^6.46^ (Fig. 2c). The bottom 4-bromo-2,6-dimethoxy-aryl moiety hydrophobically interacts with Met206^3.47^, E209^3.50^, Gly210^3.51^, Leu213^3.54^, Phe284^5.51^, Leu287^5.54^, Ile290^5.57^, and Phe362^7.56^ (Fig. 2d). The BMK-I-152 structure has an ~20% larger ligand surface area than the CRF_1_R/CP376395 complex and a higher binding free energy (−4.06 kcal/mol) than CP376395 (Supplementary Fig. 7a). Specifically, BMK-I-152 and CP376395 have 21 and 12 bond residues and 23 and 22 bond residues within 4 and 5 Å, respectively. Moreover, compared with CP376395, BMK-I-152 forms additional bonds with Val359 (Supplementary Fig. 7b, c). These findings indicate that the different binding affinities of these residues may explain the structural differences between CRF_1_R, BMK-I-152, and CP376395.

### Structure-inspired discovery of a CRF_1_R antagonist

**Fig. 3:**
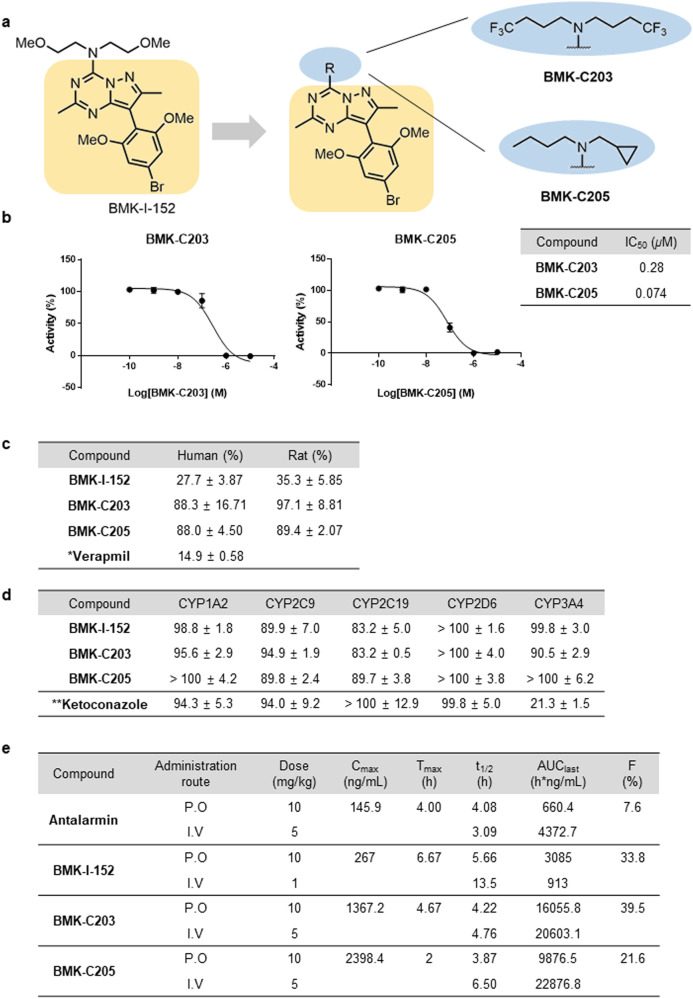
Drug profile of BMK-C203 and BMK-C205. **a** Structure-guided BMK-I-152 analog synthesis that replaced the exocyclic alkylamino group of BMK-I-152. **b** The IC_50_ values of BMK-C203 and BMK-C205 (*n* = 3). **c** Liver microsomal stabilities (percentage remaining after 30 min) (*n* = 3). *Verapamil: reference chemical for liver microsomal stability (1 μM). **d** CYP450 inhibition assay (percentage of control activity at 10 μM). *Ketoconazole: CYP3A4 inhibitor (0.1 μM), reference chemical for the CYP450 inhibition assay (*n* = 3). **e** PK profiles of antalarmin, BMK-I-152, BMK-C203, and BMK-C205 in SD rats (*n* = 3).

Since the 2,6-dimethoxy and pyrazolo[1,5-α][1,3,5]triazine core regions of BMK-I-152 are held in position by very tight binding interactions with the receptor, there is not much room for improvement. Therefore, this part remained unchanged when new antagonists were designed based on the crystal structure of the complex. We focused on modifying the exocyclic alkylamino group because the middle and pendant groups are rigid. Although BMK-I-152 exhibits highly potent binding activity (IC_50_ = 0.026 μM), its metabolic stability is poor because its dimethoxy groups are vulnerable to O-dealkylation^18^. Therefore, we chose to develop antagonists with a binding force similar to that of BMK-I-152 without metabolic stability issues. We performed molecular docking analyses using the crystal structure of the CRF_1_R/BMK-I-152 complex as a model to predict the binding models of various BMK-I-152 analogs to CRF_1_R. We used GlideScore to predict the binding affinities of Compounds 1a to 1s with CRF_1_R (Supplementary Fig. 8a). Based on the docking results, which are shown as docking scores that correspond to binding strengths in decreasing order (Supplementary Fig. 8b), the antagonists that fit the receptor best are presented (Supplementary Fig. 8c–e). An analysis of the docking complex indicated that residue Asn283 formed an essential hydrogen bond with the pyridine nitrogen in the CRF_1_R antagonist and that residue Phe203 was involved in forming a π–π stacking interaction with antagonist 1n (Supplementary Fig. 8a). Our attempts to synthesize antagonist 1n were not successful. Next, we synthesized antagonists 1f and 1i (Supplementary Fig. 8b). These exhibited better docking scores and were used to design several antagonists; these antagonists helped construct amine derivatives possessing similar chain lengths or functional groups in these structures (Supplementary Fig. 9a). In addition, we explored fluoro-substituted isosteres for C–H groups, as in –OCF_3_. However, attempts to synthesize –OCF_3_-substituted antagonists did not yield the desired products^46^. Therefore, we surmised that BMK-C203 with trifluoromethyl groups at both ends of the chain might be a suitable ligand. We successfully created the CRF_1_R antagonist BMK-C201-C210 following the same procedure used to prepare BMK-I-152. Variations in chain length did not significantly affect binding affinity. Supplementary Fig. 9b, c shows the activity and IC_50_ of BMK-C201-C210 measured by a calcium mobilization assay. Among the synthesized derivatives, BMK-C201-C210 exhibited IC_50_ values ranging from 0.28 to 0.074 μM (Fig. 3b). Although their activities were three- to 11-fold less potent than that of BMK-I-152, BMK-C201-C210 were identified as the most effective antagonists.

We selected some compounds and tested them for liver microsomal stability to develop CRF_1_R antagonists as potential drug candidates that can be administered orally and predict the liver first-pass effect. BMK-C207, BMK-C209, and BMK-C210 were not selected because of their high IC_50_ values (Supplementary Fig. 9). The remaining candidates were incubated with human and rat liver microsomes for 30 min and assessed. BMK-I-152 showed low liver microsomal stability (below 35% remaining) in almost all species (Fig. 3c) because of the methoxy groups known for poor metabolic stability^47–49^. For the same reason, BMK-C201 and BMK-C206 were not selected (data not shown). In contrast, introducing trifluoromethyl and cyclopropyl groups improved the metabolic stability of BMK-C203 and BMK-C205 by more than 90% (Fig. 3a, c). We also performed absorption, distribution, metabolism, and excretion (ADME) tests, such as CYP450 inhibition, to predict the possibility of drug‒drug interactions between these BMKs and other selected compounds (Fig. 3d)^50^. BMK-C203 and BMK-C205 exhibited minimal inhibition against five major CYP450 isoforms at a concentration of 10 μM. Additionally, the BRET assay revealed that BMK-I-152, BMK-C203, and BMK-C205 exhibited IC_50_ values that were at least 100 times lower than those of CRF_2_R in CRF_1_R (Supplementary Fig. 10). Therefore, we conducted in vivo pharmacokinetic (PK) studies using BMK-C203 and BMK-C205.

We performed PK studies using male Sprague Dawley (SD) rats and intravenous (i.v.) (BMK-I-152: 1 mg/kg; the other antagonist: 5 mg/kg) or oral (p.o.) (10 mg/kg) gavage administration to address the oral bioavailability of BMK-I-152, BMK-C203, BMK-C205, and antalarmin (Fig. 3e, Supplementary Fig. 11). Peceol oil, Tocofersolan (TPGS), and polyethylene glycol (PEG) 400 were used as treatment vehicles to counter the low aqueous solubility of all the antagonists. The PK profiles of Antalarmin, BMK-I-152, BMK-C203, and BMK-C205 in rats showed *F* values of 7.6%, 33.8%, 39.5%, and 21.6%, respectively (Fig. 3e, Supplementary Fig. 11). BMK-I-152, BMK-C203, and BMK-C205 exhibited improved oral bioavailability compared to Antalarmin. In contrast to BMK-C203 and BMK-C205, BMK-I-152 resulted in a relatively low *C*_max_ value. This demonstrates that the BMK-I-152 level in the blood is significantly low even soon after i.v. injection. These findings suggested that BMK-C203 and BMK-C205 were better suited as effective oral drugs than BMK-I-152 and antalarmin in vitro and in vivo.

### Comparative analysis of the CRF_1_R/BMK-I-152, CRF_1_R/BMK-C203, and CRF_1_R/BMK-C205 structures

**Fig. 4:**
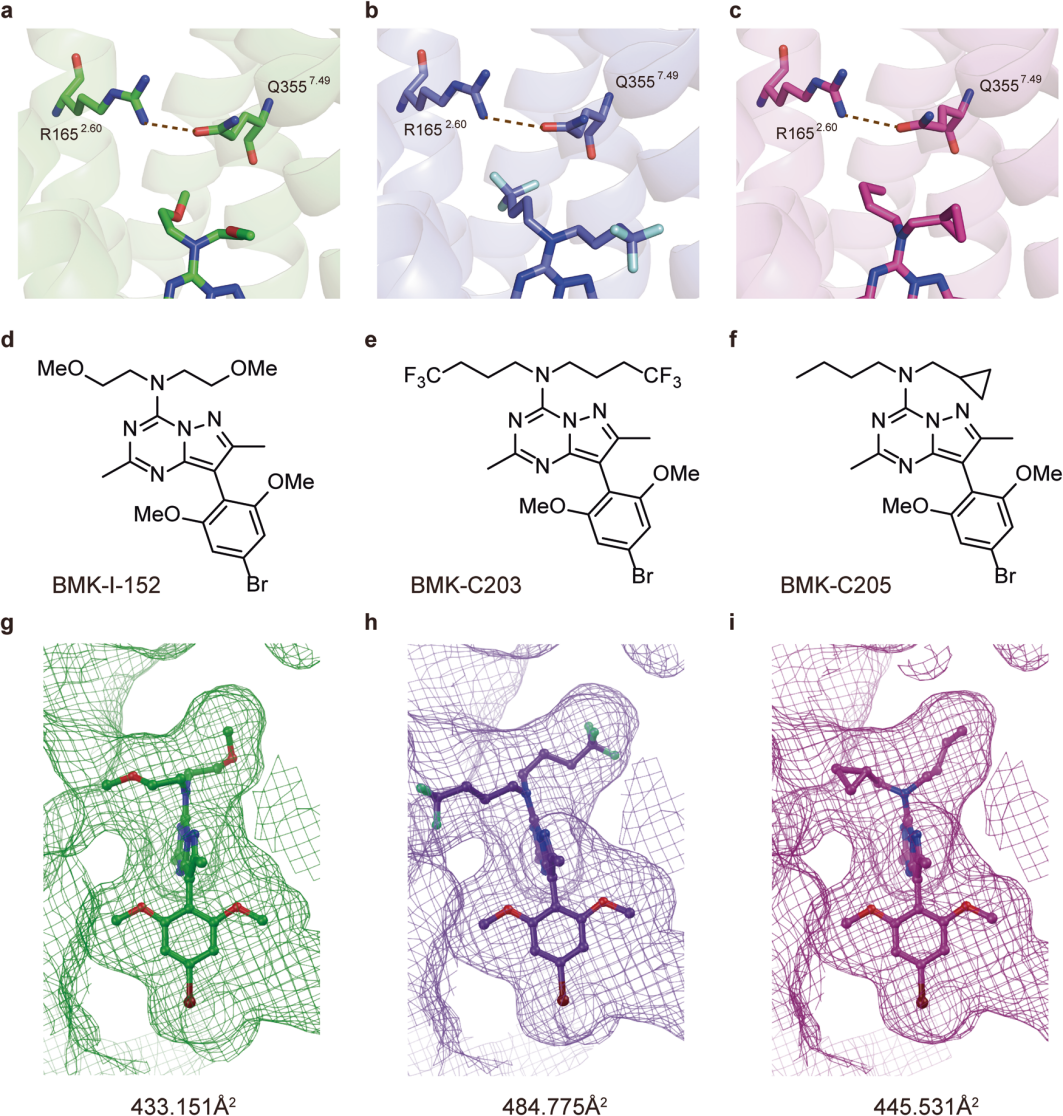
Comparison of the CRF_1_R, BMK-I-152, BMK-C203, and BMK-C205 structures. The hydrogen bond R165^2.60^–Q355^7.49^, the key bond of the hydrogen-central hydrogen bond network, was compared between CRF_1_R and BMK-I-152, BMK-C203, and BMK-C205. Brown dotted lines denote hydrogen bonds. The hydrogen bond distances are **a** 3.0 Å in CRF_1_R with BMK-I-152, **b** 3.4 Å in CRF_1_R with BMK-C203, and **c** 3.3 Å in CRF_1_R with BMK-C205. CRF_1_R antagonist structure of **d** BMK-I-152, **e** BMK-C203, and **f** BMK-C205. Calculating the ligand surface areas of **g** BMK-I-152, **h** BMK-C203, and **i** BMK-C205.

The structures of the receptor-antagonist complexes formed using the newly synthesized BMK-C203 and BMK-C205 compounds were determined via the same method used for the CRF_1_R/BMK-I-152 complex. The antagonists were positioned in a hydrophobic network similar to that of BMK-I-152 (Fig. 4a–c). The root-mean-square deviation (RMSD) values for the Cα of the BMK-C203 and BMK-C205 complex structures were 0.217 and 0.228, respectively, compared to that of BMK-I-152 (Fig. 4d–f). The hydrogen bonds between R165^2.60^ and Q355^7.49^ and H199^3.40^ and Y327^6.53^ in the central hydrogen bond network were similar to those in the CRF_1_R/BMK-I-152 complex. Asn283 formed hydrogen bonds with BMK-C203 and BMK-C205. BMK-C203 and BMK-C205 interacted with 23 residues of CRF_1_R, such as BMK-I-152 (Supplementary Fig. 4b). However, each compound had a different IC_50_ value and chemical structure. We observed structural differences between the complexes formed with each antagonist. First, the ligand surface areas of BMK-C203 and BMK-C205 increased by approximately 12% and 3%, respectively, compared to that of BMK-I-152 (Fig. 4g–i). Second, the bond distances between the conserved inactivation hydrogen bonds in each R165^2.60^ and Q355^7.49^ structure were different, and those of BMK-I-152, BMK-C203, and BMK-C205 were 3.0, 3.4, and 3.3 Å, respectively. In addition, weak hydrogen bonding was observed between –CF3 and Q355^7.49^ in the CRF_1_R complex with BMK-C203.

### Antidepressant effect of CRF_1_R antagonists

**Fig. 5:**
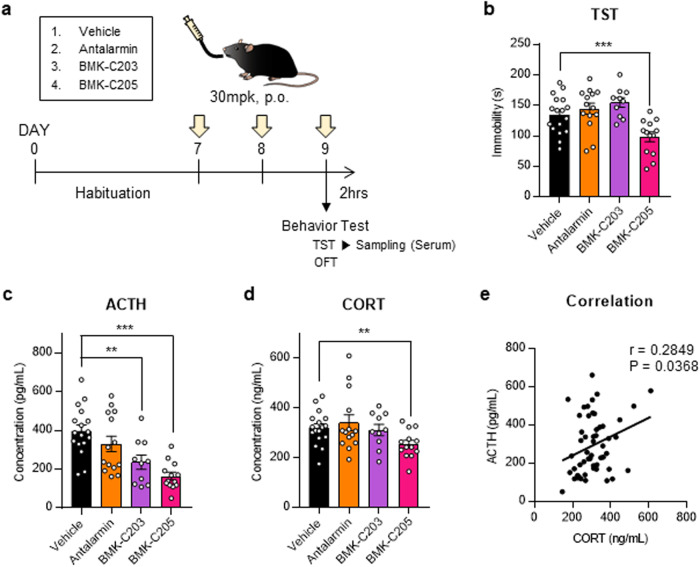
Antidepressant effects of CRF_1_R antagonists in a mouse model. **a** Timeline for behavioral testing was conducted 2 h after three consecutive days of drug treatment. Serum was isolated from the mice immediately after the TST application. The TST activates the HPA axis as a stressor. **b** Immobility times in drug-treated mice showed that only the BMK-C205 group was more mobile than the vehicle group. **c** ACTH concentrations in the serum from BMK-C203- and BMK-C205-treated mice were lower than those in the vehicle group. **d** CORT serum concentrations decreased in BMK-C205-treated mice compared to those in the vehicle group (vehicle *n* = 17, antalarmin *n* = 14, BMK-C203 *n* = 10, and BMK-C205 *n* = 13; **p < 0.01, ***p < 0.001, ANOVA). **e** ACTH serum levels were positively correlated with CORT levels (*N* = 54, Pearson’s *r* = 0.2849).

**Fig. 6:**
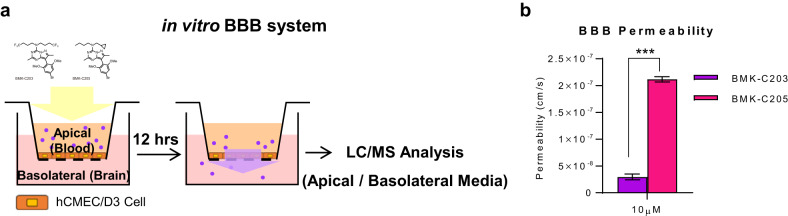
Drug permeability test using an in vitro BBB system. **a** Schematic of the in vitro BBB system used to measure drug permeability through a monolayer of hCMEC/D3 cells. **b** The BBB permeability of BMK-C205 was approximately seven times higher than that of B.

A TST was conducted after a CRF_1_R antagonist drug (30 mg/kg) was orally administered for 3 days to investigate the in vivo behavioral effects of the new CRF_1_R antagonists (Fig. 5a). TSTs were performed 2 h after the third drug treatment because the drug concentration in the blood was found to be highest from 2 to 6 h after p.o. administration (Supplementary Fig. 11). The immobility times of mice treated with the vehicle and CRF_1_R antagonists were measured (Fig. 5b). BMK-C205-treated mice had significantly decreased immobility times compared to vehicle-treated mice, indicating that BMK-C205 exerted an antidepressant effect in vivo. There were no differences between overall moving distances in the open field test (OFT), indicating that decreased immobility time caused by BMK-C205 was not due to hyperactivity in mice (Supplementary Fig. 11). These results suggested that BMK-C205 exerts antidepressant effects without causing hyperactivity. Since the TST is a stressor that activates the HPA axis, we surmised that the ACTH and CORT serum levels in mice, which increased immediately following the TST, were reduced by CRF_1_R antagonists that blocked HPA axis activation. Serum ACTH levels in the BMK-C203 and BMK-C205 groups decreased (Fig. 5c), and the latter also exhibited decreased serum CORT levels (Fig. 5d). In addition, serum ACTH and CORT concentrations were positively correlated (Fig. 5e). We further measured the blood‒brain barrier (BBB) permeability of the antagonists using an in vitro BBB system (Fig. 6a) and observed that the permeability coefficient of BMK-C205 was seven times higher than that of BMK-C203 (Fig. 6b) (BMK-C203, 2.96 × 10^−8^ cm/s; BMK-C205, 2.11 × 10^−7^ cm/s; ***p < 0.001). These results suggested that the higher BBB permeability of BMK-C205 may result in an effective in vivo antidepressant response in mice.

## Discussion

We determined the structures of CRF_1_R/high-affinity antagonist complexes using an XFEL. Three structures were examined at resolutions of 2.75, 2.6, and 2.2 Å, and we observed that these CRF_1_R structures differed from previous CRF_1_R structures and other class B GPCR structures. First, the inactive state of most class B GPCRs carries only one hydrogen bond in the central hydrogen bond network. However, CRF_1_R carries two main hydrogen bonds. Our CRF_1_R structure carried a hydrogen bond at R165^2.60^–Q335^7.49^, unlike previously determined CRF_1_Rs. Despite similarities between our structure and a previously identified CRF_1_R/CP376395 complex structure (RMSD value: 0.766), the R165^2.60^–Q355^7.49^ hydrogen bond in the central hydrogen bond network of the CRF_1_R/CP376395 complex structure was absent. However, the presence of the R165^2.60^–Q355^7.49^ hydrogen bond in the CRF_1_R/BMK-I-152, CRF_1_R/BMK-C203, and CRF_1_R/BMK-C205 complexes were confirmed (Supplementary Fig. 4b). Considering that the R165^2.60^–Q335^7.49^ hydrogen bond is conserved in the inactive compositions of other class B GPCRs, our structure may be more inactivated than CRF_1_R/CP376395. Since our forms were determined using an XFEL at room temperature, the configuration of the side chain may partly differ from that of the other structures determined via a synchrotron. However, this difference may not be due to the diffraction method used because the CRF_1_R/CP376395 structure contains a weak R165^2.60^–Y195^3.36^ hydrogen bond, which is also present in its CRF_1_R active state structure (Supplementary Fig. 5e–g). Thus, compared to the previous CRF_1_R structure, our structure may have been more inactivated. Second, although our structure depicts only the TMD, we determined the structure of its stalk part and confirmed that it assumes an alpha-helix conformation. Changes in the stalk region are crucial in the activation mechanism of class B GPCRs. Furthermore, the stalk of the inactive conformation of each class B GPCR structure was different^44^. Thus, our results offer a structure-based perspective on the CRF_1_R activation mechanism.

We employed a fixed target method using a sample holder made of ketone film for XFEL diffraction experiments, which conferred that structural studies possess a notable advantage. First, much smaller protein crystals were needed than those involved in the lipid cubic phase (LCP) injector method. The LCP injector method forces protein crystals to flow, causing many crystals to be wasted without affecting diffraction; this is problematic because these protein crystals cannot be reused. However, through the fixed target method, the same sample holder and crystals can be used once more for diffraction. Second, the fixed target method is user-friendly, whereas the LCP injector method requires another lipid to be mixed to achieve a suitable stickiness for the XFEL sample. However, mixing protein crystals is potentially damaging and requires a suitable type of lipid and mixing ratio to be determined. However, no crystal mix-up is needed in our fixed target method using ketone films, making sample preparation straightforward. Due to these advantages, high-resolution CRF_1_R structures could be uncovered without the associated technical challenges regarding the types and amounts of protein crystals being used.

We conducted structure-based drug discovery studies on CRF_1_R, a class B GPCR that mediates the stress response. CRF_1_R is a drug target for anxiety, depression, and many other stress-related disorders. We designed and synthesized various CRF_1_R antagonists based on insights obtained from the crystal structure of the CRF_1_R/BMK-I-152 complex. Several antagonists were synthesized by changing alkyl chain lengths or introducing functional groups, such as CF_3_. Examination of CYP450 inhibition propensity indicated that none of our selective compounds (BMK-C203 and BMK-C205) significantly inhibited the five representative CYP enzymes CYP1A2, CYP2C9, CYP2C19, CYP2D6, and CYP3A4. In human and rat liver microsomal stability tests, BMK-C203 and BMK-C205 showed much lower metabolic instability tendencies than that of BMK-I-152 following 30 min of incubation. In addition, PK studies of BMK-C203 and BMK-C205 showed acceptable levels of oral bioavailability. Although not selected by us, BMK-C202, BMK-C204, and BMK-C208 possess an exocyclic alkylamino group with similar characteristics to BMK-C203 and BMK-C205, indicating the potential for higher microsomal stability and lower drug‒drug interactions. However, due to their similar properties to BMK-C203, BMK-C204, and C208 are expected to have low BBB drug permeability. In contrast, BMK-C202 may yield promising results in animal experiments, similar to those of BMK-C205.

Conventional antidepressants based on serotonin or noradrenaline system modulation are ineffective for some depressive patients and only relieve depressive symptoms after weeks or months. Therefore, it has been suggested that HPA axis regulation may be a new antidepressant target. Since increased HPA axis activation is commonly observed in patients with depression, we aimed to downregulate the HPA axis via CRF_1_R antagonists. In our study, antalarmin, a known CRF_1_R antagonist, showed no significant difference in a behavioral test and produced ACTH and CORT serum levels. Our findings are consistent with a previous study in which no alteration in serum ACTH and CORT levels was observed after antalarmin was administered for 1–8 weeks following immobilization stress^51^. However, administration of BMK-C205 orally reduced HPA axis activation caused by a stressor, as evidenced by a reduction in ACTH and CORT serum concentrations. Thus, we suggest that BMK-C205, our new CRF_1_R antagonist, may be used as a novel candidate compound for downregulating the HPA axis and alleviating depressive behavior in vivo. To assess the antidepressant effects of chemicals, we employed a stressed mouse model immediately after exposure to stressors to gauge the effects of candidate chemicals in suppressing hyperactivity of the HPA axis. While many studies have used nonstressed mice to test potential antidepressants^52–54^, using depression models, such as chronic unpredictable, restraint and social defeat stressed mice, may be more appropriate. Therefore, it would be desirable to examine the effects of BMK-C205 in these depression models using prolonged stressors.

BMK-C205 displayed a better antidepressant effect than that of BMK-C203. We showed that BMK-C205 has an IC_50_ value approximately fourfold lower than that of BMK-C203 in our hCRF_1_R Ca^2+^ antagonism assays (Fig. 3b). The serum concentration of BMK-C205 was approximately twice as high as that of BMK-C203 2 h after oral administration in the PK test (Fig. 3e, Supplementary Fig. 12). However, those differences could not verify that BMK-C205 exhibited a better antidepressant effect. Therefore, we tried to find another difference between these antagonists. We confirmed that BMK-C205 exhibits a higher BBB permeability than that of BMK-C203, as supported by our in vitro BBB assay (Fig. 6). BBB permeability is a vital factor when determining the efficacy of CNS-targeting drugs. The BBB, composed of brain endothelial cells and supported by astrocytes and pericytes, is crucial in maintaining CNS homeostasis by restricting molecular transport from blood to the brain. Out of 7000 drugs, only 5% can cross the BBB and directly affect brain function^55^. Our results showed that after the same concentration of the drugs was orally administered, BMK-C205 in the blood more effectively passed through the BBB than BMK-C203. Therefore, we suggest that BMK-C205 may induce more effective antidepressant responses, including less HPA axis activation (lower ACTH and CORT levels) and behavioral changes (less immobility in the TST), as it can better pass into the brain due to its BBB-related properties. Although BMK-C203 did not significantly affect the TST and CORT levels, it significantly affected ACTH levels, which correspond to a direct CRF_1_R downstream signal. Therefore, compared to BMK-C205, it was presumed that BMK-C203 at a lower concentration does not sufficiently inhibit CRF_1_R.

In summary, we determined the crystalline structure of CRF_1_R using antagonists and an XFEL. We then used docking simulations based on our CRF_1_R structure to develop new antagonists and selected two via calcium mobilization, CYP inhibition, microsomal stability, and PK studies in mice. We then conducted a depressive behavioral test using our newly developed antagonists (BMK-C203 and BMK-C205). The results showed a significant difference between the two, indicating that BMK-C205 was superior to BMK-C203. Furthermore, we found that ACTH and CORT levels were significantly decreased in mice treated with BMK-C205 compared to other mice. Thus, these results provide insights into techniques used to discover new types of drugs that target CRF_1_R, thereby alleviating certain stress-related diseases.

### Supplementary information

Supplementary

structure file_8GTM

structure file_8GTI

structure file_8GTG

validation report_8GTM

validation report_8GTI

validation report_8GTG

## Data Availability

Density maps and structural coordinates were deposited in the Protein Data Bank (PDB) under the following accession numbers: 8GTG for the CRFR_BMK-I-152 complex, 8GTM for the CRF1R_BMK-C203 complex, and 8GTI for the CRFR_BMK-C205 complex. All data are available in the main text or the supplementary materials.
