# Supplementary material for: Structure-based drug discovery of a corticotropin-releasing hormone receptor 1 antagonist using an X-ray free-electron laser
Source: Exp Mol Med. 2023 Sep 1;55(9):2039–50. doi: 10.1038/s12276-023-01082-1 (PMC10545732; doi:10.1038/s12276-023-01082-1)
Supplement: Supplementary file 1 — Supplementary [file 12276_2023_1082_MOESM1_ESM.docx]

**Supplementary Table 1. Data collection and refinement statistics**

|  | CRF_1_R_BMK-I-152 | CRF_1_R _BMK-C203 | CRF_1_R _BMK-C205 |
| --- | --- | --- | --- |
| **Data collection** |  |  |  |
| Space group | C 1 2 1 | C 1 2 1 | C 1 2 1 |
| Cell dimensions |  |  |  |
| *a*, *b*, *c* (Å) | 95.66, 70.65, 86.75 | 95.66, 70.65, 86.75 | 95.66, 70.65, 86.75 |
| α, β, γ (°) | 90.00, 97.82, 90.00 | 90.00, 97.82, 90.00 | 90.00, 97.82, 90.00 |
| Resolution (Å) | 44.11–2.75 (2.848–2.75)* | 56.64–2.60 (2.86–2.60)* | 56.64–2.20 (2.27–2.20)* |
| *R_split_* | 0.1221 (1.1069) | 0.1428 (0.9335) | 0.0736 (1.0750) |
| *I* / σ*I* | 5.50 (1.17) | 4.87 (1.36) | 7.40 (1.10) |
| Completeness (%) | 100 (100) | 100 (100) | 100 (100) |
| Redundancy | 264.7 (180.9) | 113.8 (75.9) | 429.2 (287.1) |
|  |  |  |  |
| **Refinement** |  |  |  |
| Resolution (Å) | 44.11–2.75 | 56.64–2.60 | 56.64–2.20 |
| No. reflections | 15048 | 17776 | 29223 |
| *R*_work_ / *R*_free_ | 0.2354/0.2630 | 0.2193/0.2602 | 0.2108/0.2407 |
| No. atoms |  |  |  |
| Protein | 3214 | 3266 | 3287 |
| Ligand/ion | 59 | 64 | 116 |
| Solvent | 0 | 0 | 0 |
| *B*-factors |  |  |  |
| Protein | 86.75 | 66.60 | 70.92 |
| Ligand/ion | 69.55 | 55.40 | 79.70 |
| Solvent |  |  |  |
| RMSDs |  |  |  |
| Bond lengths (Å) | 0.009 | 0.012 | 0.011 |
| Bond angles (°) | 1.02 | 0.94 | 1.05 |

*Values in parentheses are for the highest resolution shell. CRF_1_R with BMK-I-152, BMK-C203, and BMK-C205 used 13689, 14485, and 74311 crystals, respectively.


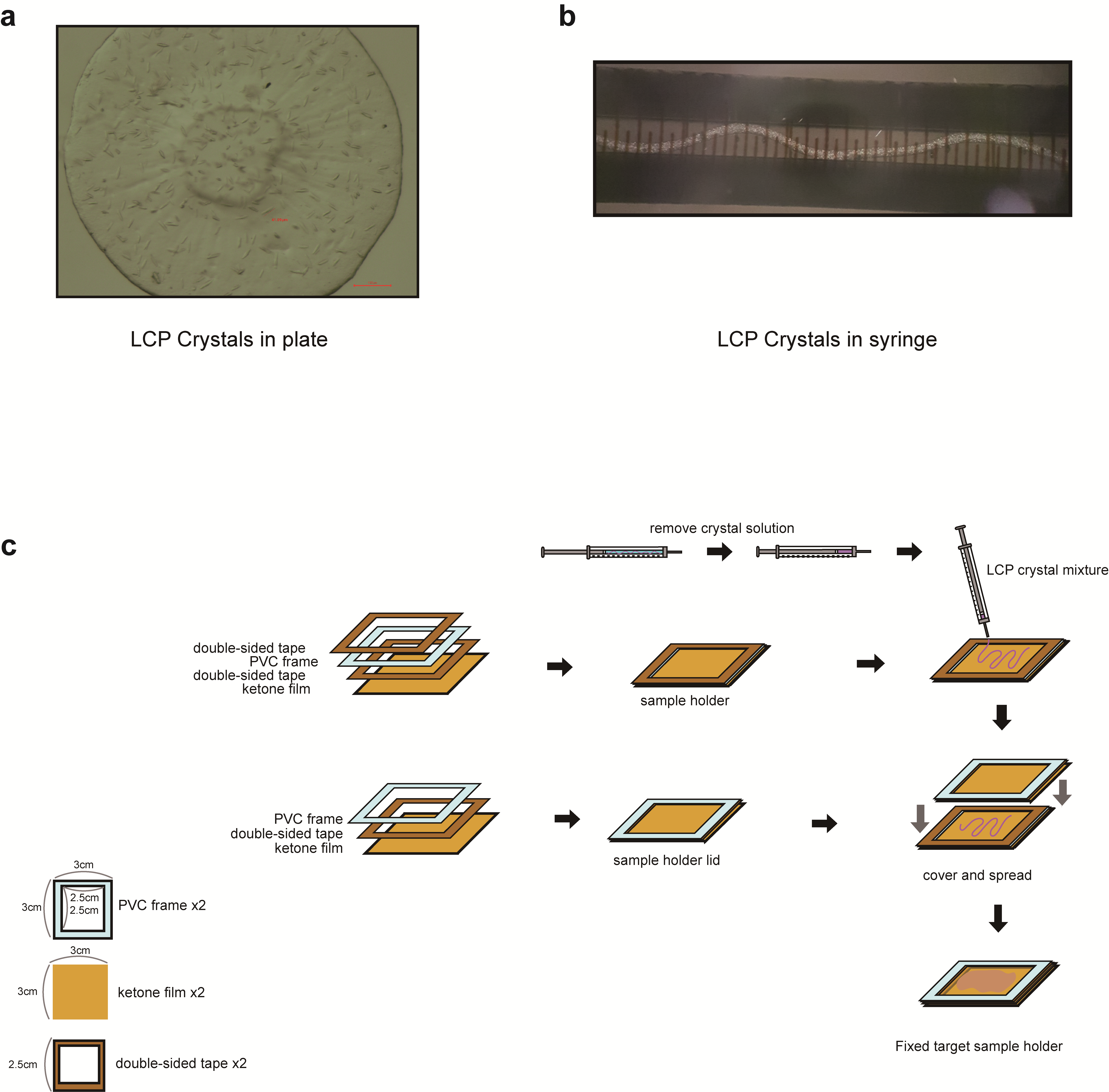


**Supplementary Fig. 1. (a)** CRF_1_R with antagonist crystals using a synchrotron radiation beamline and **(b)** XFEL. **(c)** A schematic showing how to produce a fixed target sample holder for the fixed target method.


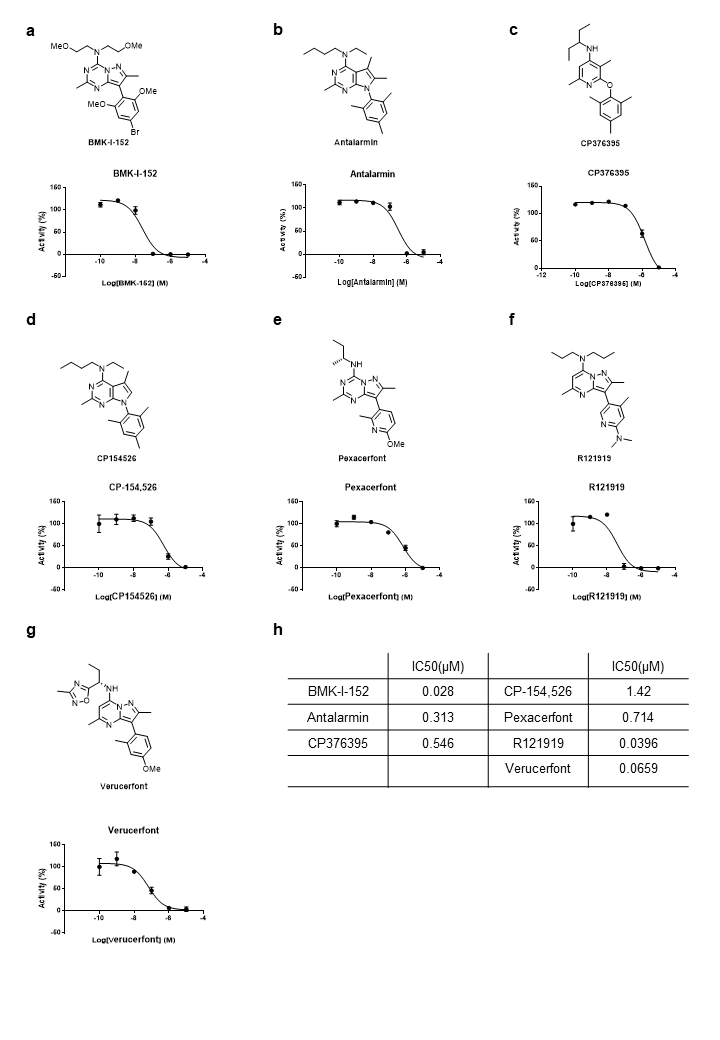


**Supplementary Fig. 2. Comparison between the IC_50_ values of BMK-I-152 and commercially available antagonists.** **(a)** BMK-I-152, **(b)** Antalarmin, **(c)** CP376395, **(d)** CP-154,526, **(e)** Pexacerfont, **(f)** R121919, and **(g)** Verucerfont calcium mobilization assay profiles, normalized to activity percentages. Error bars, standard error of the mean (SEM)(n = 3 independent experiments, biological replicates). **(h)** Antagonist IC_50_ values.


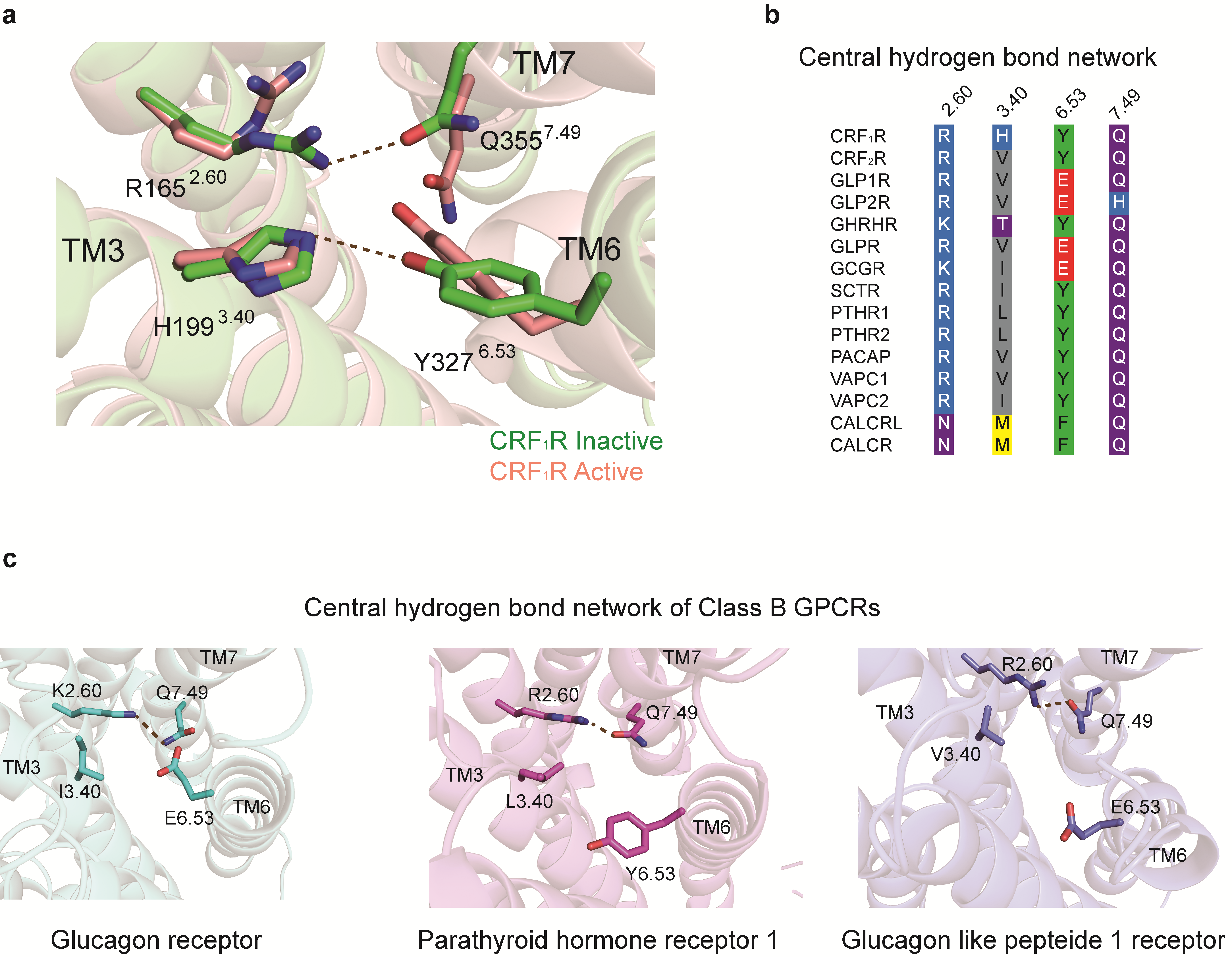


**Supplementary Fig. 3. (a)** Comparing the CRF_1_R inactive and activate state structures (PDB code: 6P9X). **(b)** Sequence alignment of the central hydrogen bond networks in all class B GPCRs. **(c)** Comparison between central hydrogen bond networks in GCGR (PDB code: 5XEZ), parathyroid hormone receptor 1 (PDB code: 6FJ3), and GLP1 receptor (PDB code: 5VEW), which inactivates state structures.





**Supplementary Fig. 4. (a)** Alignment of the inactivated CRF_1_R structure with BMK-I-152, BMK-C203, BMK-C205, and CP376395. All antagonists are in the orthosteric ligand-binding site of the hydrophobic network. **(b)** Comparison between CRF_1_R with BMK-C203, BMK-C205, and CP376395 in inactive state motif networks.


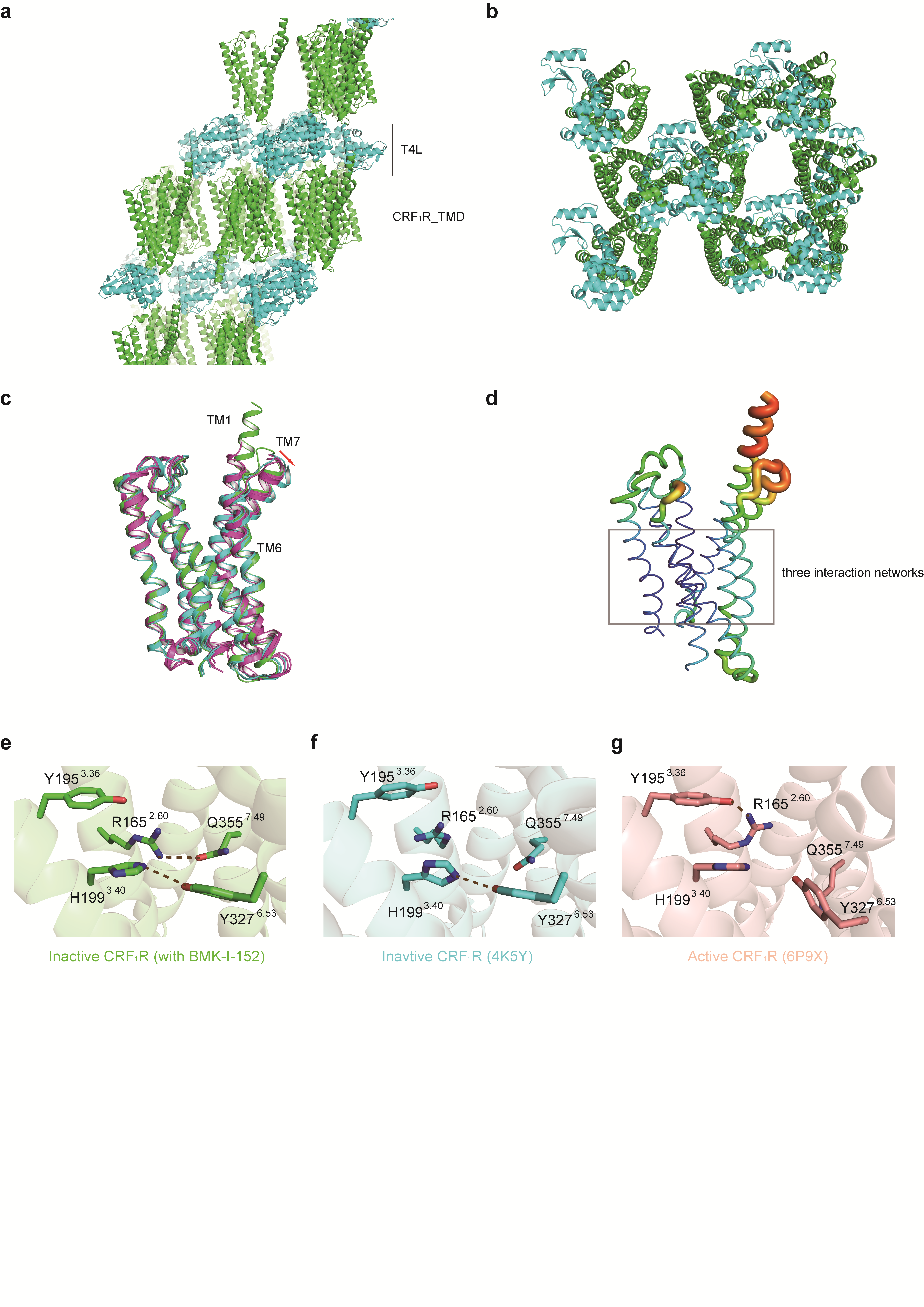


**Supplementary Fig. 5. (a)** Crystal packing of CRF_1_R with BMK-I-152 in the monoclinic setting view along a unique a axis, and **(b)** unique c axis. CRF_1_R and T4L are in ribbon representations and colored green and cyan, respectively. **(c)** Structural alignment between our CRF_1_R structure and previous CRF_1_R structures. All CRF_1_R structures are shown as ribbon representations. The CRF_1_R structure with BMK-I-152, the A, B, and C chain structure of CRF_1_R with CP376395 (PDB code: 4K5Y), and the A, B, and C chain structure of CRF_1_R with CP376395 (PDB code: 4Z9G) are colored in green, cyan, and magenta, respectively. **(d)** CRF_1_R with BMK-I-152, as sausage representations, colored using a relative B-factor spectrum (blue=low; red=high). The gray box denotes the position of three interaction networks. Comparison between the inactivated structure with BMK-I-152, CP376395, and the activated state structure. **(e)** The CRF_1_R structure with BMK-I-152 has two inactivation hydrogen bond motifs (R165^2.60^-Q355^7.49^ and H199^3.40^-Y327^6.53^). **(f)** The CRF_1_R structure with CP376395 has one inactivation hydrogen bond motif (H199^3.40^-Y327^6.53^) and one activation hydrogen bond motif (R165^2.60^-Y195^3.36^). **(g)** The CRF_1_R structure has one activation hydrogen bond motif (R165^2.60^-Y195^3.36^).


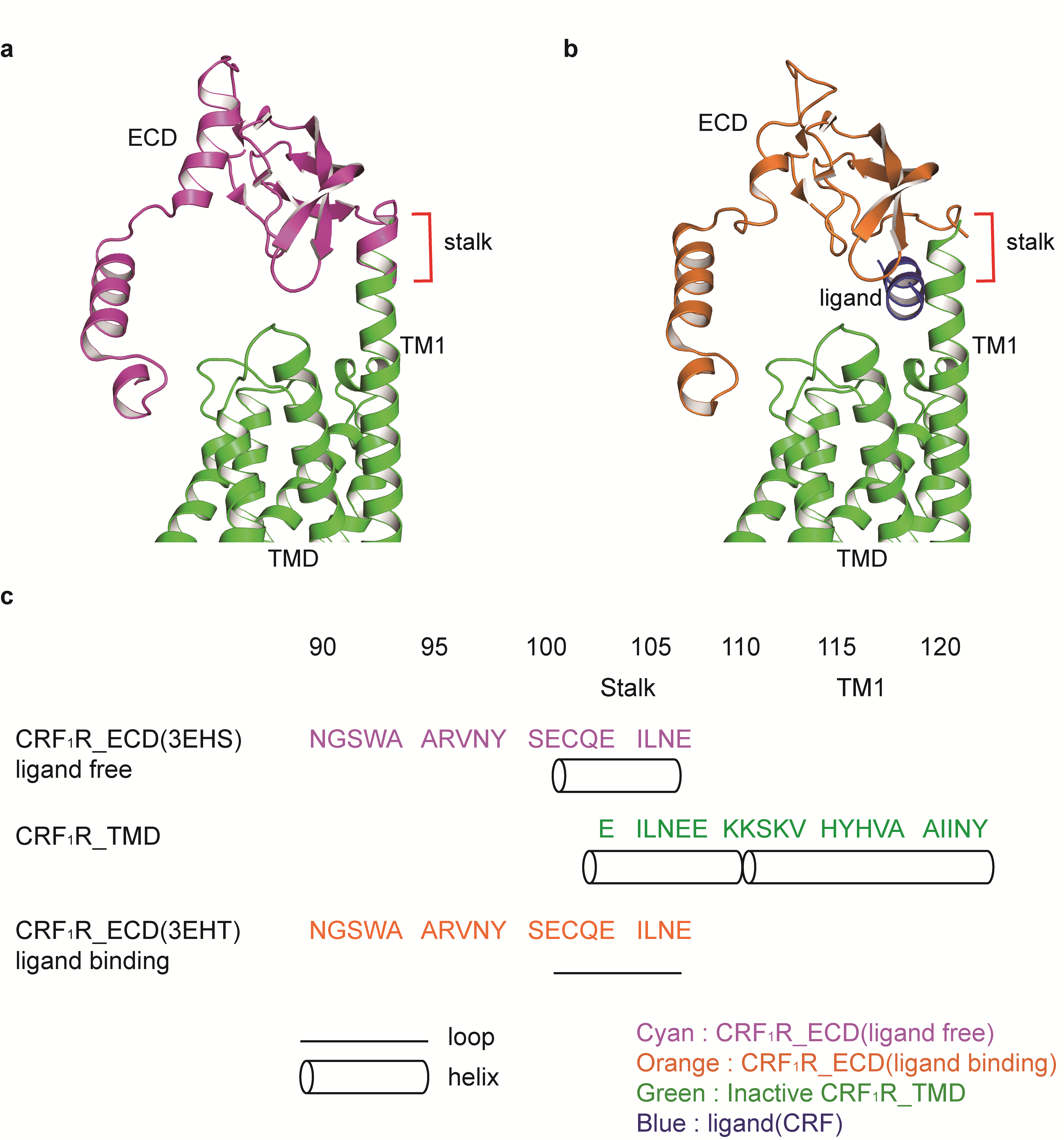


**Supplementary Fig. 6.** **(a)** Structural alignment based on the stalk, using our CRF_1_R structure and the ligand-free CRF_1_R ECD structure (PDB code: 3EHS). **(b)** Structural alignment based on the stalk using our CRF_1_R structure and the ligand-binding CRF_1_R ECD structure (PDB code: 3EHT). **(c)** Amino acid sequence alignment of the nearby stalk domain of **(a)** and **(b)**.


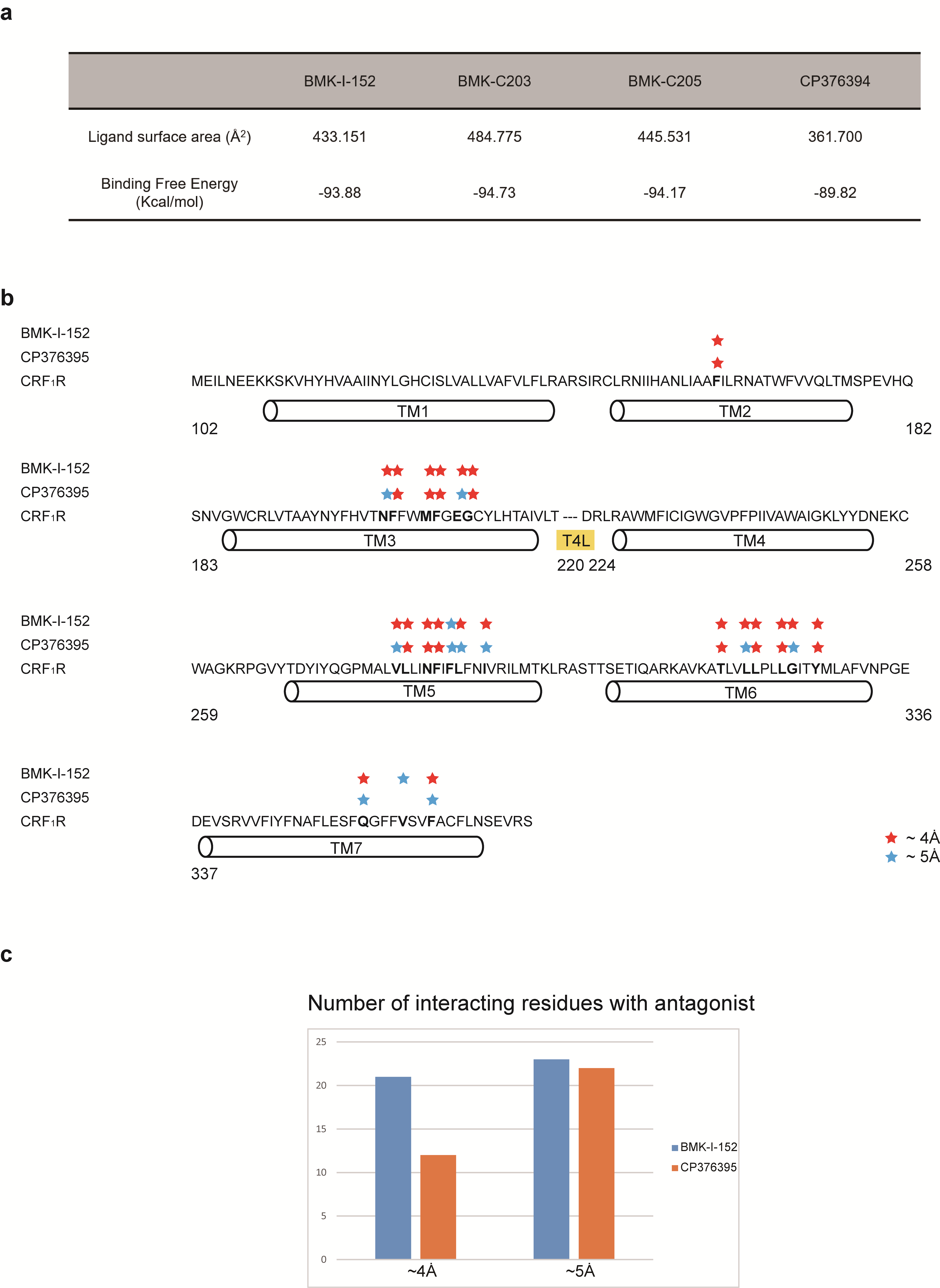


**Supplementary Fig. 7. (a)** Calculating the binding free energy of each ligand in CRF_1_R. Maestro 11.5 (Schrodinger LCC, New York) was used to calculate the binding site area and binding free energy. **(b)** The CRF_1_R structures with BMK-I-152 or CP376395, showing the residues within 4 Å of antagonists, are marked with a red star. Residues within 5 Å are marked with a blue star. **(c)** A graph indicating the number of red and blue stars in **(c)**.

**Supplementary Fig. 8. (a)** The 2D structure of BMK-I-152 analogs. **(b)** Molecular docking score from 1a to 1s. The 3D structure of CRF_1_R with **(c)** 1n, **(d)** 1i, and **(e)** 1f.


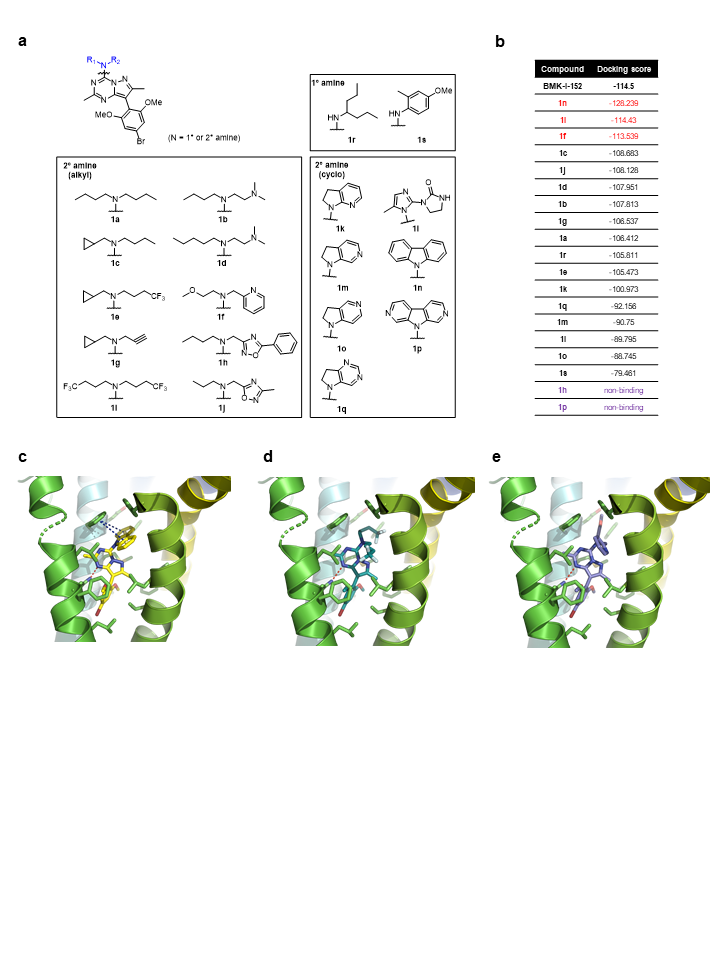

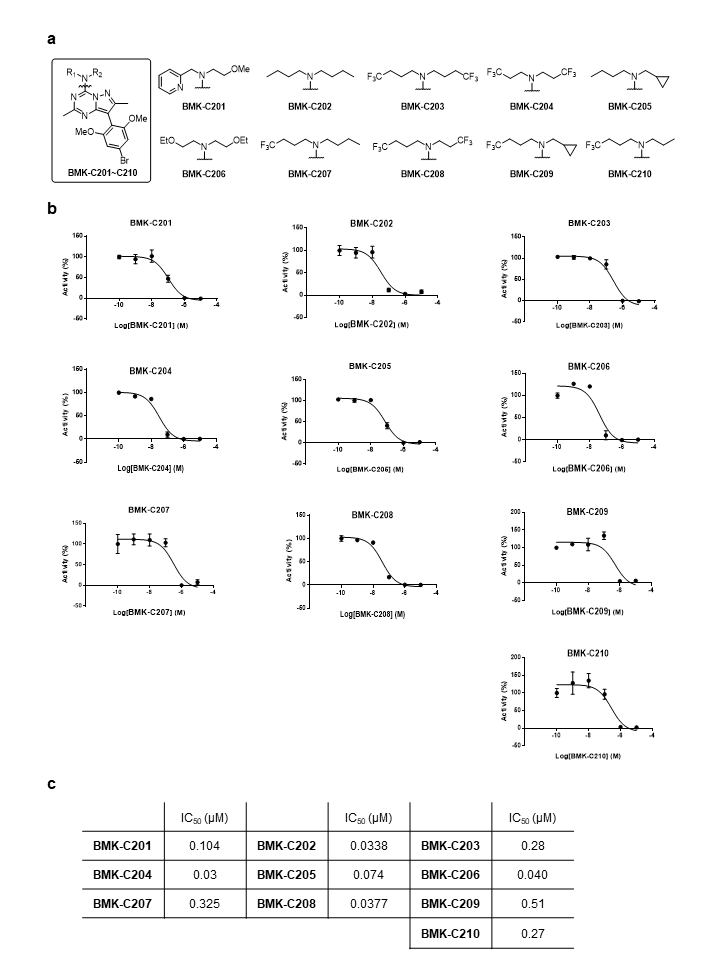


**Supplementary Fig. 9. (a)** Structure-guided synthesis of BMK-I-152 analogs (BMK-C201 to C210) replaced the exocyclic alkylamino group of BMK-I-152. **(b)** The IC_50_ values of BMK-C201 to C210 via a calcium mobilization assay normalized to activity percentages. Error bars, SEM (n = 3 independent experiments, biological replicates). **(c)** IC_50_ values of BMK-C201 to C210.

**Supplementary Fig. 10.** Measurement of IC50 values ​​in CRF_1_R and CRF_2_R of BMK-I-152, BMK-C203 and BMK-C205 through BRET assay (n = 3 independent experiments, biological replicates).


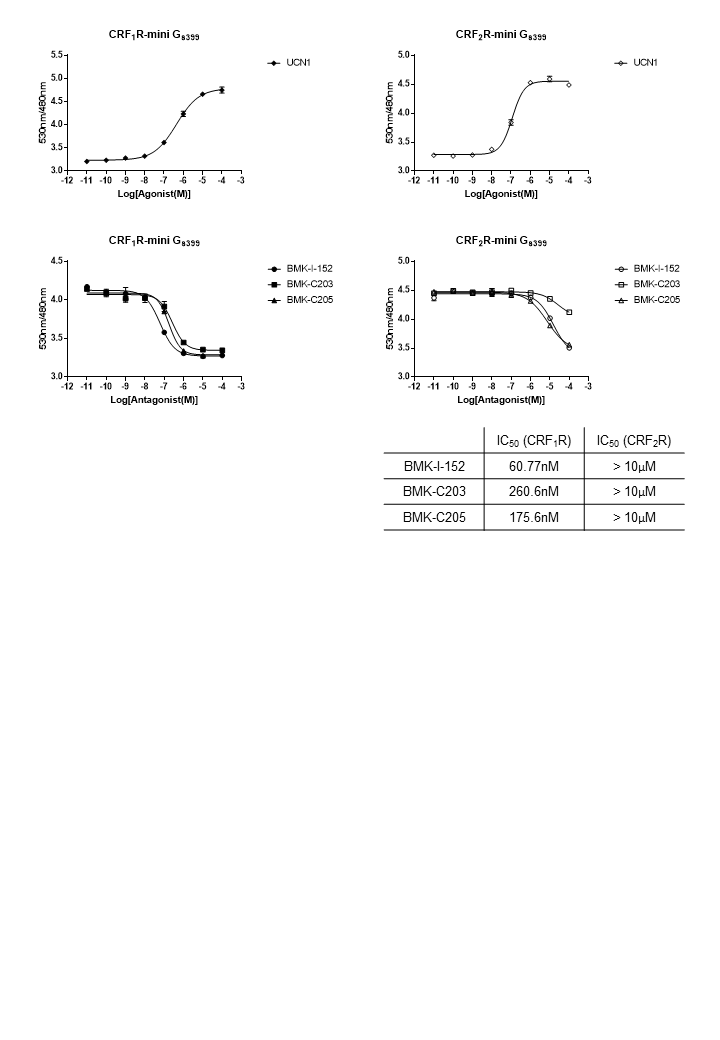


**Supplementary Fig. 11.** Plasma concentrations after p.o. and i.v. administration of Antalarmin, BMK-C203, BMK-C205, and BMK-I-152 in SD rats (n = 3).


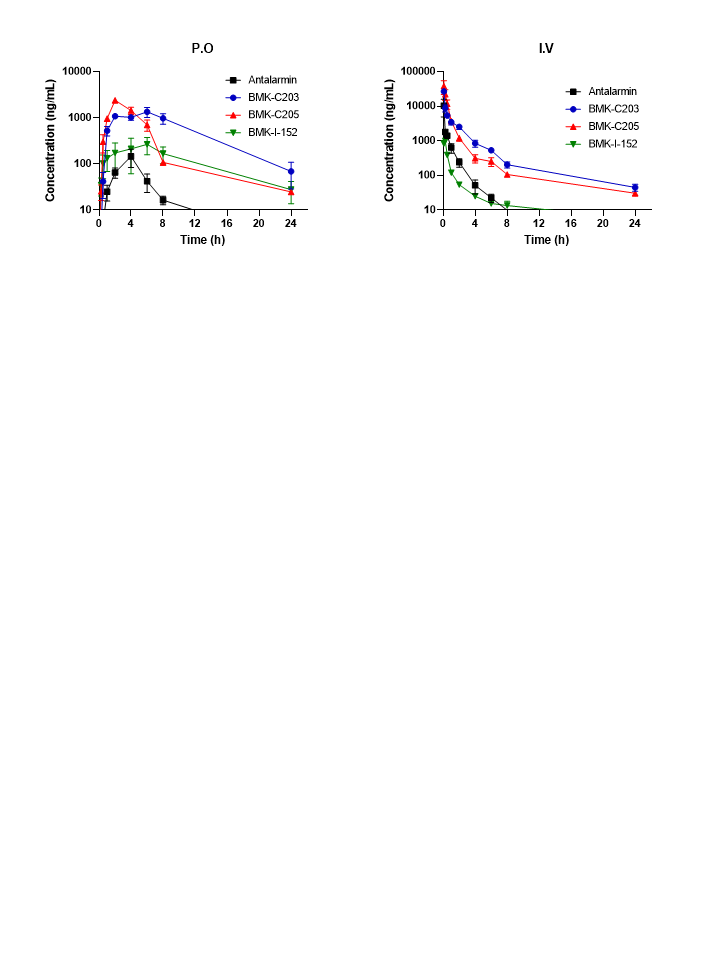

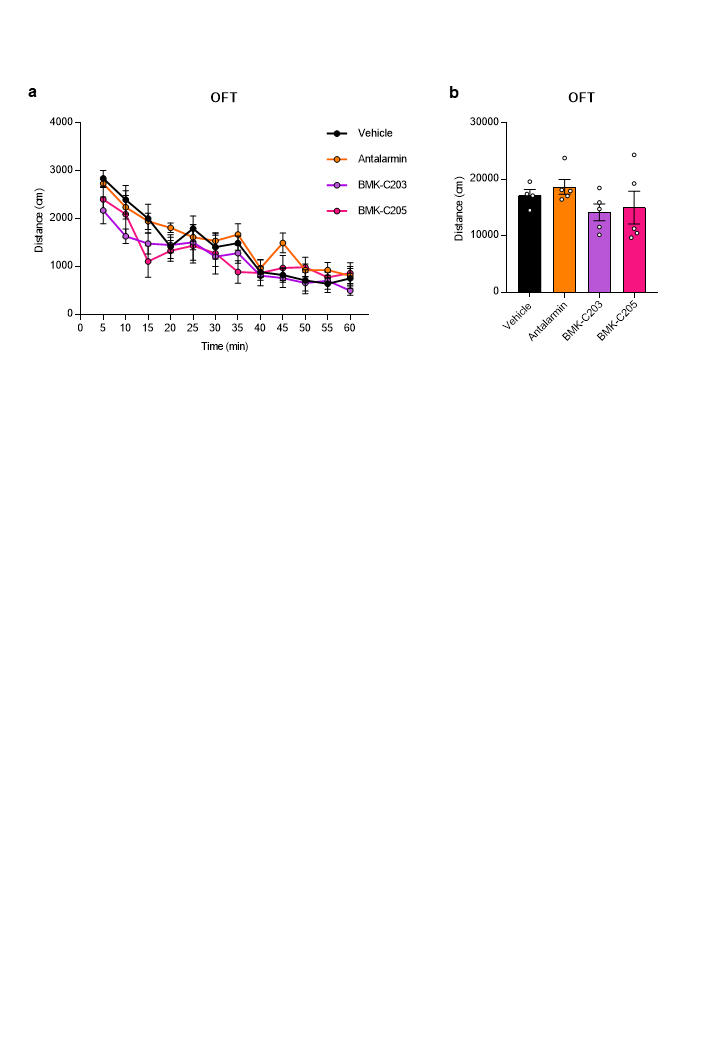


**Supplementary Fig. 12. (a)** Locomotion in CRF_1_R antagonist-treated mouse models. Locomotion during the OFT was scored in 5-min intervals and did not differ among groups. **(b)** Total movements also did not differ among groups (vehicle n = 4, Antalarmin n = 5, BMK-C203 n = 5, and BMK-C205 n = 5).

**Supplementary Data. 1.**

Synthetic Procedures: General Information

All commercial reagents were used without further purification. BMK-I-152 and compound 1 were synthesized per the literature procedure^1^. Thin layer chromatography (TLC) was performed using Merck silica gel 60 F_254_, and spots were visualized under a 254 nm UV light and/or stained by ceric ammonium molybdate solutions. Column chromatography was performed on silica gel (Merck 9385 Kiesel gel 60) using hexane/ethyl acetate as an eluent. ^1^H, ^13^C NMR, and ^19^F spectra were recorded on an Agilent 400–MR DD2 (^1^H, 400 MHz; ^13^C, 101 MHz; ^19^F, 376 MHz) or ^1^H and ^13^C NMR spectra were recorded on a Varian/Oxford As-500 spectrophotometer (^1^H, 500 MHz; ^13^C, 126 MHz). Chemical shift values were recorded as parts per million (δ) relative to tetramethylsilane as an internal standard with coupling constants in Hertz (Hz). The following abbreviations were used to explain the multiplicities: s = singlet, d = doublet, t = triplet, q = quartet, m = multiplet, and br = broad. High-resolution mass spectra (HRMS) were recorded on an AB SCIEX Quadrupole-TOF Mass Spectrometer.

*^a^* Reaction conditions: (i) Dialkylamine (NHR_1_R_2_), DIPEA, THF, room temperature, or dialkylamine·HCl, TEA, MeOH. (ii) 10% Pd/C, H_2_, room temperature. (iii) Tf_2_O, pyridine, DCM, 0°C to room temperature. (iv) Bu_3_SnSnBu_3_, Pd(PPh_3_)_4_, LiCl, BHT, 1,4-Dioxane, 100°C. (v) NBS, THF, room temperature.

Synthesis and Characterization of BMK-C201~C210

**8-(4-(Benzyloxy)-2,6-dimethoxyphenyl)-4-chloro-2,7-dimethylpyrazolo[1,5-α][1,3,5]triazine (1).** 8-(4-benzyloxy-2,6-dimethoxyphenyl)-2,7-dimethyl-3H-pyrazolo[1,5-α][1,3,5]triazin-4-one (0.50 g, 1.23 mmol) was added to a two-neck round bottom flask equipped with a reflux condenser. The flask was evacuated and backfilled with inert gas three times. Dry toluene (12.3 mL), dry *N*,*N*-diisopropylethylamine (0.64 mL, 3.67 mmol), and distilled POCl_3_ (0.23 mL, 2.47 mmol) were added via a syringe in sequence. The reaction mixture was heated to reflux for 24 h under inert gas. Then the mixture was cooled to room temperature and partitioned between ethyl acetate (7 mL) and ice water (7 mL) quickly. The organic layer was dried over MgSO_4_, filtered, and concentrated under reduced pressure. The residue was quickly purified on a short silica gel chromatographic column (n-Hex:EtOAc, 4:1 v/v). Yellow solids were collected (390 mg, 75% yield). ^1^H NMR (400 MHz, CDCl_3_): δ 7.42 (m, 5H), 6.33 (s, 2H), 5.11 (s, 2H), 3.71 (s, 6H), 2.61 (s, 3H), and 2.36 (s, 3H); ^13^C NMR (101 MHz, CDCl_3_): δ 161.1, 160.7, 159.6, 159.5, 147.7, 146.9, 136.8, 128.8, 128.3, 127.7, 104.3, 100.2, 92.1, 70.4, 55.9, 25.4, and 13.8; HRMS (m/z): ([M]^+^ calculated for C_22_H_21_ClN_4_O_3_ = 425.1302) 425.1365 (found).

**8-(4-(Benzyloxy)-2,6-dimethoxyphenyl)-*N*-(2-methoxyethyl)-2,7-dimethyl-*N*-(pyridin-2-ylmethyl)pyrazolo[1,5-α][1,3,5]triazin-4-amine (2a).** 8-(4-Benzyloxy-2,6-dimethoxyphenyl)-4-chloro-2,7-dimethylpyrazolo-[1,5-α][1,3,5]triazine (1) (424 mg, 1.0 mmol) was dissolved in dry tetrahydrofuran (THF)(5 mL). 2-methoxy-*N*-(pyridin-2-ylmethyl)ethan-1-amine (249 mg, 1.2 mmol) and dry *N*,*N*-diisopropylethylamine (0.35 mL, 2.0 mmol) were added via a syringe. The reaction mixture was stirred at room temperature for 3 h. The reaction mixture was treated with water, and the resulting mixture was extracted with ethyl acetate (3 × 10 mL), dried over MgSO_4_, filtered, and concentrated in vacuo. The crude product was purified by silica gel flash chromatography to produce a white solid (338 mg, 61% yield). TLC (n-Hex:EtOAc, 1:1 v/v): *R*_f_ = 0.14; ^1^H NMR (500 MHz, CDCl_3_): δ 8.57 (s, 1H), 7.62 (t, *J* = 6.6 Hz, 1H), 7.47 (d, *J* = 6.2 Hz, 2H), 7.40 (s, 3H), 7.34 (d, *J* = 6.3 Hz, 1H), 7.16 (s, 1H), 6.33 (s, 2H), 5.59 (s, 2H), 5.09 (s, 2H), 4.33 (s, 2H), 3.81 (s, 2H), 3.71 (s, 6H), 3.35 (s, 3H), 2.38 (s, 3H), and 2.19 (s, 3H); ^13^C NMR (126 MHz, CDCl_3_): δ 161.8, 160.3, 159.5, 158.1, 154.7, 150.0, 149.2 (d, *J* = 4.4 Hz), 137.0, 136.5, 128.6, 128.0, 127.6, 122.1, 101.9, 99.6, 92.2, 70.2, 58.8, 56.8–55.5 (m), 49.5, 25.7, and 13.5.

**3,5-Dimethoxy-4-(4-((2-methoxyethyl)(pyridin-2-ylmethyl)amino)-2,7-dimethylpyrazolo[1,5-α][1,3,5]triazin-8-yl)phenol (3a).** 10% Pd/C (de Gussa type, Aldrich) was added to a solution of 8-(4-(benzyloxy)-2,6-dimethoxyphenyl)-*N*-(2-methoxyethyl)-2,7-dimethyl-*N*-(pyridin-2-ylmethyl)pyrazolo[1,5-α][1,3,5]triazin-4-amine (2a) (544 mg, 1.0 mmol) in methanol (14 mL). The mixture was stirred in a hydrogen atmosphere at room temperature. When TLC showed no starting material, the mixture was diluted with ethyl acetate and filtered through a celite bed. The filtrate was concentrated under reduced pressure to produce a white solid (432 mg, 93% yield). TLC (n-Hex:EtOAc, 1:4 v/v): *R*_f_ = 0.18; ^1^H NMR (500 MHz, CDCl_3_): δ 8.57 (s, 1H), 7.62 (t, *J* = 6.6 Hz, 1H), 7.47 (d, *J* = 6.2 Hz, 2H), 7.40 (d, 3H), 7.34 (d, *J* = 6.3 Hz, 1H), 7.16 (s, 1H), 6.33 (s, 2H), 5.59 (s, 2H), 5.09 (s, 2H), 4.33 (s, 2H), 3.81 (s, 2H), 3.71 (s, 6H), 3.35 (s, 3H), 2.38 (s, 3H), and 2.19 (s, 3H); ^13^C NMR (126 MHz, CDCl_3_): δ 162.0, 159.6, 159.0, 155.7, 149.4, 149.3 (d, *J* = 2.2 Hz), 148.5, 136.7, 122.4, 100.6, 97.9, 93.0, 59.0 (d, *J* = 2.8 Hz), 55.9, 55.3 (d, *J* = 5.3 Hz), 49.8, 24.5 (d, *J* = 3.4 Hz), and 13.4 (d, *J* = 1.9 Hz).

**3,5-Dimethoxy-4-(4-((2-methoxyethyl)(pyridin-2-ylmethyl)amino)-2,7-dimethylpyrazolo[1,5-α][1,3,5]triazin-8-yl)phenyl trifluoromethanesulfonate (4a).** 3,5-Dimethoxy-4-(4-((2-methoxyethyl)(pyridin-2-ylmethyl)amino)-2,7-dimethylpyrazolo[1,5-α][1,3,5]triazin-8-yl)phenol (3a) (465 mg, 1.0 mmol) was dissolved in dry dichloromethane (DCM)(2.5 mL). The solution was cooled to 0℃, and dry pyridine (1.4 mL) and trifluoromethanesulfonic anhydride (0.18 mL, 1.1 mmol) were slowly added via a syringe at 0℃. The reaction mixture was stirred at 0℃ for 30 min and then at room temperature overnight. The mixture was quenched by water and extracted with DCM (3 × 10 mL). Combined organic layers were dried over anhydrous MgSO_4_, filtered, and concentrated. The crude product was purified on a silica gel chromatographic column to produce a white solid (322 mg, 54% yield). TLC (n-Hex:EtOAc, 1:4 v/v): *R*_f_ = 0.17; ^1^H NMR (400 MHz, CDCl_3_): δ 8.56 (d, *J* = 4.2 Hz, 1H), 7.64 (td, *J* = 7.7, 1.7 Hz, 1H), 7.39 (d, *J* = 7.9 Hz, 1H), 7.18 (dd, *J* = 6.7, 5.1 Hz, 1H), 6.53 (s, 2H), 5.57 (s, 2H), 4.32 (s, 2H), 3.80 (t, *J* = 5.5 Hz, 2H), 3.75 (s, 6H), 3.35 (s, 3H), 2.36 (s, 3H), and 2.14 (s, 3H); ^13^C NMR (126 MHz, CDCl_3_): δ 162.5, 159.5, 158.0, 154.4, 150.3, 150.1, 149.3 (d, *J* = 1.7 Hz), 149.3, 136.7, 122.3, 122.2, 120.1, 117.6, 109.5, 98.5–97.9 (m), 59.0 (d, *J* = 2.5 Hz), 56.6–56.1 (m, *J* = 9.9, 4.9 Hz), 55.9, 49.7, 25.8 (d, *J* = 2.6 Hz), and 13.6 (d, *J* = 1.5 Hz); ^19^F NMR (376 MHz, CDCl_3_): δ -72.9.

**8-(2,6-Dimethoxy-4-(tributylstannyl)phenyl)-*N*-(2-methoxyethyl)-2,7-dimethyl-*N*-(pyridin-2-ylmethyl)pyrazolo[1,5-α][1,3,5]triazin-4-amine (5a).** Lithium chloride (63.6 mg, 1.5 mmol), tetrakis(triphenylphosphine)palladium (2.9 mg, 5 mol%), one 2,6-di-tert-butyl-4-methylphenol crystal, and hexabutylditin (0.51 mL, 1.0 mmol) under argon were added to a trifluoromethanesulfonic acid 3,5-dimethoxy-4-(4-((2-methoxyethyl)(pyridin-2-ylmethyl)amino)-2,7-dimethylpyrazolo[1,5-α][1,3,5]triazin-8-yl)phenyl trifluoromethanesulfonate (4a) (298 mg, 0.5 mmol) in dioxane (2.5 mL) solution. The mixture was heated to 100℃ for 5 h then cooled to room temperature. It was diluted with ethyl acetate and washed with 10% aqueous ammonium hydroxide solution. The organic layer was filtered through a celite bed. The filtrate was dried over anhydrous MgSO_4_ and concentrated. The crude product was purified on a silica gel chromatographic column to produce a white solid (166 mg, 45% yield). TLC (n-Hex:EtOAc, 1:1 v/v): *R*_f_ = 0.13; ^1^H NMR (500 MHz, CDCl_3_): δ 8.57 (d, *J* = 4.8 Hz, 1H), 7.65 (t, *J* = 7.7 Hz, 1H), 7.41 (d, *J* = 7.9 Hz, 1H), 7.21–7.15 (m, 1H), 6.72 (d, *J* = 0.7 Hz, 2H), 5.57 (s, 4H), 4.33 (s, 4H), 3.81 (s, 2H), 3.77 (s, 6H), 3.36 (s, 3H), 2.37 (s, 3H), 2.19 (s, 3H), 1.67–1.50 (m, 6H), 1.42–1.32 (m, 6H), 1.16–1.00 (m, 6H), and 0.95–0.88 (m, *J* = 7.2, 0.7 Hz, 9H); ^13^C NMR (126 MHz, CDCl_3_): δ 162.0, 158.2, 154.8, 150.0, 149.3, 149.3, 143.2, 136.6, 122.2, 112.3 (d, *J* = 18.0 Hz), 109.3, 99.9, 59.0 (d, *J* = 3.0 Hz), 56.2 (d, *J* = 7.8 Hz), 55.9, 49.6, 29.3, 27.6, 25.9 (d, *J* = 4.0 Hz), 13.9, and 9.9.

**8-(4-Bromo-2,6-dimethoxyphenyl)-*N*-(2-methoxyethyl)-2,7-dimethyl-*N*-(pyridin-2-ylmethyl)pyrazolo[1,5-α][1,3,5]triazin-4-amine (BMK-C201).** 8-(2,6-Dimethoxy-4-(tributylstannyl)phenyl)-*N*-(2-methoxyethyl)-2,7-dimethyl-*N*-(pyridin-2-ylmethyl)pyrazolo[1,5-α][1,3,5]triazin-4-amine (5a) (148 mg, 0.2 mmol) was dissolved in dry THF (1 mL). N-BromcoscinimideNBS (34 mg, 0.19 mmol) was added to the solution. When TLC showed no starting material, the solvent was removed in vacuo. The residue was purified on a silica gel chromatographic column to produce white solids (63 mg, 60% yield). TLC (n-Hex:EtOAc, 1:1 v/v): *R*_f_ = 0.18; ^1^H NMR (500 MHz, CDCl_3_): δ 8.58 (s, 1H), 7.65 (t, *J* = 7.1 Hz, 1H), 7.39 (d, *J* = 7.6 Hz, 1H), 7.19 (s, 1H), 6.76 (s, 2H), 5.57 (s, 2H), 4.33 (s, 2H), 3.80 (s, 2H), 3.73 (s, 6H), 3.35 (s, 3H), 2.35 (s, 3H), and 2.15 (s, 3H); ^13^C NMR (126 MHz, CDCl_3_): δ 177.7, 162.3, 159.4, 158.1, 154.5, 149.9, 149.3, 136.7, 122.5, 108.2, 98.8, 59.0 (d, *J* = 3.2 Hz), 56.2 (d, *J* = 9.0 Hz), 55.9, 49.7, 29.7, 25.6, and 13.6; HRMS (m/z): ([M]^+^ calculated for C_24_H_27_BrN_6_O_3_ = 527.1401) 527.1392 (found).

**8-(4-(Benzyloxy)-2,6-dimethoxyphenyl)-*N*,*N*-dibutyl-2,7-dimethylpyrazolo[1,5-α][1,3,5]triazin-4-amine (2b).** Compound 2b was prepared per the procedure described for synthesizing 2a. The title compound was obtained (393 mg, 76% yield) as a white solid. TLC (n-Hex:EtOAc, 2:1 v/v): *R*_f_ = 0.48; ^1^H NMR (400 MHz, CDCl_3_): δ 7.48 (d, *J* = 7.3 Hz, 2H), 7.42 (t, *J* = 7.4 Hz, 2H), 7.38–7.31 (m, *J* = 7.2 Hz, 1H), 6.32 (s, 2H), 5.10 (s, 2H), 3.99 (s, 4H), 3.73 (s, 6H), 2.37 (s, 3H), 2.21 (s, 3H), 1.80–1.68 (m, 4H), 1.47–1.37 (m, *J* = 14.7, 7.4 Hz, 4H), and 0.99 (t, *J* = 7.4 Hz, 6H); ^13^C NMR (101 MHz, CDCl_3_): δ 161.9, 160.4, 159.6, 154.3, 150.1, 148.9, 137.1, 128.8, 128.2, 127.7, 102.5, 99.1, 92.4, 70.4, 56.1 (d, *J* = 2.4 Hz), 50.2, 46.2, 30.9, 25.9, 20.4, 20.2, 14.0, and 13.6.

**4-(4-(Dibutylamino)-2,7-dimethylpyrazolo[1,5-α][1,3,5]triazin-8-yl)-3,5-dimethoxyphenol (3b).** Compound 3b was prepared per the procedure described for synthesizing 3a. The title compound was obtained (393 mg, 92% yield) as a pale yellow solid. TLC (n-Hex:EtOAc, 2:1 v/v): *R*_f_ = 0.18; ^1^H NMR (400 MHz, CDCl_3_): δ 5.84 (s, 2H), 4.01 (s, 4H), 3.59 (s, 6H), 2.47 (s, 3H), 2.18 (s, 3H), 1.78 (s, 4H), 1.42 (q, *J* = 14.6, 7.3 Hz, 4H), and 1.00 (t, *J* = 7.3 Hz, 6H); ^13^C NMR (126 MHz, CDCl_3_): δ 162.0, 159.7, 159.0, 155.3, 149.0, 148.5, 100.1, 98.1, 93.1, 55.4 (d, *J* = 5.1 Hz), 50.5, 29.9, 24.6, 20.2, 14.0, and 13.5.

**4-(4-(Dibutylamino)-2,7-dimethylpyrazolo[1,5-α][1,3,5]triazin-8-yl)-3,5-dimethoxyphenyl trifluoromethanesulfonate (4b).** Compound 4b was prepared per the procedure described for synthesizing 4a. The title compound was obtained (313 mg, 56% yield) as a white solid. TLC (n-Hex:EtOAc, 7:1 v/v): *R*_f_ = 0.28; ^1^H NMR (500 MHz, CDCl_3_): δ 6.54 (s, 2H), 3.99 (s, 4H), 3.76 (s, 6H), 2.38 (s, 3H), 2.18 (s, 3H), 1.80–1.70 (m, *J* = 15.1, 7.6 Hz, 4H), 1.45–1.36 (m, 4H), and 0.99 (t, *J* = 7.4 Hz, 6H); ^13^C NMR (126 MHz, CDCl_3_): δ 162.5, 159.5, 153.8, 150.2, 148.9, 120.2, 117.6, 110.0, 98.3, 97.6, 60.5, 56.3, 50.3, 25.8, 20.2, 14.3, 14.0, and 13.6; ^19^F NMR (376 MHz, CDCl_3_): δ -73.0.

***N,N*-Dibutyl-8-(2,6-dimethoxy-4-(tributylstannyl)phenyl)-2,7-dimethylpyrazolo[1,5-α][1,3,5]triazin-4-amine (5b).** Compound 5b was prepared per the procedure described for synthesizing 5a. The title compound was obtained (147 mg, 42% yield) as a white solid. TLC (n-Hex:EtOAc, 7:1 v/v): *R*_f_ = 0.48; ^1^H NMR (500 MHz, CDCl_3_): δ 6.73 (s, 2H), 3.99 (s, 4H), 3.78 (s, 6H), 2.38 (s, 3H), 2.23 (s, 3H), 1.82–1.71 (m, 4H), 1.68–1.53 (m, 6H), 1.45–1.34 (m, 10H), 1.16–1.03 (m, 6H), 0.99 (t, *J* = 7.4 Hz, 6H), and 0.93 (t, *J* = 7.3 Hz, 9H); ^13^C NMR (126 MHz, CDCl_3_): δ 161.9, 158.2, 154.2, 150.1, 148.9, 142.9, 112.3, 109.7, 99.3, 56.2 (d, *J* = 6.5 Hz), 50.2, 30.9, 29.3, 27.6, 25.9 (d, *J* = 3.3 Hz), 20.2, 13.9 (d, *J* = 19.7 Hz), 11.2 (d, *J* = 7.4 Hz), 9.9, and 8.6 (d, *J* = 7.6 Hz).

**8-(4-Bromo-2,6-dimethoxyphenyl)-*N,N*-dibutyl-2,7-dimethylpyrazolo[1,5-α][1,3,5]triazin-4-amine (BMK-C202).** Compound BMK-C202 was prepared per the procedure described for synthesizing BMK-C201. The title compound was obtained (58.9 mg, 60% yield) as a white solid. TLC (n-Hex:EtOAc, 7:1 v/v): *R*_f_ = 0.24; ^1^H NMR (500 MHz, CDCl_3_): δ 6.79 (s, 2H), 3.98 (s, 4H), 3.74 (s, 6H), 2.37 (s, 3H), 2.18 (s, 3H), 1.74 (s, 4H), 1.40 (q, *J* = 14.1, 6.9 Hz, 4H), and 0.98 (t, *J* = 7.2 Hz, 6H); ^13^C NMR (126 MHz, CDCl_3_): δ 162.3, 159.3, 153.8, 150.1, 148.8, 122.3, 108.8, 108.4, 98.2, 56.3 (dd, *J* = 17.3, 8.0 Hz), 50.2, 30.9, 25.9 (d, *J* = 5.1 Hz), 20.2, 14.0, and 13.6 (d, *J* = 3.8 Hz); HRMS (m/z): ([M]^+^ calculated for C_23_H_32_BrN_5_O_2_ = 490.1812) 490.1804 (found).

**8-(4-(Benzyloxy)-2,6-dimethoxyphenyl)-2,7-dimethyl-*N,N*-bis(4,4,4-trifluorobutyl)pyrazolo[1,5-α][1,3,5]triazin-4-amine (2c).** Compound 2c was prepared per the procedure described for synthesizing 2a. The title compound was obtained (393 mg, 63% yield) as a white solid. TLC (n-Hex:EtOAc, 2:1 v/v): *R*_f_ = 0.32; ^1^H NMR (400 MHz, CDCl_3_): δ 7.49 (d, *J* = 7.4 Hz, 2H), 7.43 (t, *J* = 7.4 Hz, 2H), 7.36 (t, *J* = 7.1 Hz, 1H), 6.34 (s, 2H), 5.12 (s, 2H), 4.05 (s, 4H), 3.74 (s, 6H), 2.40 (s, 3H), 2.35–2.15 (m, 7H), and 2.14–2.03 (m, 4H); ^13^C NMR (101 MHz, CDCl_3_): δ 161.9, 160.5, 159.6, 155.2, 149.9, 149.0, 137.1, 131.2, 128.8, 128.5, 128.2, 127.7, 125.8, 101.9, 99.8, 92.3, 70.3 (d, *J* = 4.2 Hz), 56.0 (d, *J* = 2.5 Hz), 49.0, 31.3 (q, *J* = 29.1 Hz), 25.8, 21.6, and 13.6; ^19^F NMR (376 MHz, CDCl_3_): δ -66.1 (t, *J* = 10.8 Hz).

**4-(4-(Bis(4,4,4-trifluorobutyl)amino)-2,7-dimethylpyrazolo[1,5-α][1,3,5]triazin-8-yl)-3,5-dimethoxyphenol (3c).** Compound 3c was prepared per the procedure described for synthesizing 3a. The title compound was obtained (482 mg, 90% yield) as a white solid. TLC (n-Hex:EtOAc, 1:1 v/v): *R*_f_ = 0.27; ^1^H NMR (400 MHz, CDCl_3_): δ 5.86 (s, 2H), 4.32–3.85 (m, 4H), 3.61 (s, 6H), 2.51 (s, 3H), 2.34–2.22 (m, *J* = 17.1, 8.8 Hz, 4H), 2.20 (s, 3H), and 2.13 (d, *J* = 6.1 Hz, 4H); ^13^C NMR (101 MHz, CDCl_3_): δ 171.3, 162.2, 159.7, 159.0, 156.1, 149.1, 148.4, 131.2, 128.5, 125.9, 125.7, 123.0, 100.9, 97.8, 93.1, 60.5, 55.4 (d, *J* = 2.1 Hz), 49.3, 31.3 (q, *J* = 29.6 Hz), 24.6, 14.3, and 13.4; ^19^F NMR (376 MHz, CDCl_3_): δ -66.0 (t, *J* = 10.5 Hz).

**4-(4-(Bis(4,4,4-trifluorobutyl)amino)-2,7-dimethylpyrazolo[1,5-α][1,3,5]triazin-8-yl)-3,5-dimethoxyphenyl trifluoromethanesulfonate (4c).** Compound 4c was prepared per the procedure described for synthesizing 4a. The title compound was obtained (367 mg, 55% yield) as a white solid. TLC (n-Hex:EtOAc, 5:1 v/v): *R*_f_ = 0.17; ^1^H NMR (500 MHz, CDCl_3_): δ 6.56 (s, 2H), 4.05 (s, 4H), 3.77 (s, 6H), 2.39 (s, 3H), 2.30–2.20 (m, 4H), 2.19 (s, 3H), and 2.14–2.05 (m, 4H); ^13^C NMR (126 MHz, CDCl_3_): δ 162.5, 159.5, 154.7, 150.4, 150.1, 149.0, 130.4, 128.2, 126.0, 123.8, 122.7, 120.2, 117.6, 109.4, 98.4, 98.3 (d, *J* = 7.8 Hz), 56.4 (d, *J* = 10.1 Hz), 49.2, 31.3 (q, *J* = 29.2 Hz), 25.8 (d, *J* = 5.8 Hz), 21.7, and 13.6 (d, *J* = 4.0 Hz); ^19^F NMR (376 MHz, CDCl_3_): δ -66.1 (t, *J* = 10.7 Hz), -72.9.

**8-(2,6-Dimethoxy-4-(tributylstannyl)phenyl)-2,7-dimethyl-*N,N*-bis(4,4,4-trifluorobutyl)pyrazolo[1,5-α][1,3,5]triazin-4-amine (5c).** Compound 5c was prepared per the procedure described for synthesizing 5a. The title compound was obtained (186 mg, 46% yield) as a white solid. TLC (n-Hex:EtOAc, 7:1 v/v): *R*_f_ = 0.24; ^1^H NMR (499 MHz, CDCl_3_): δ 6.73 (s, 2H), 4.05 (s, 4H), 3.78 (s, 6H), 2.38 (s, 3H), 2.30–2.18 (m, 7H), 2.13–2.04 (m, 4H), 1.67–1.52 (m, 6H), 1.43–1.33 (m, 6H), 1.17–1.01 (m, 6H), and 0.93 (t, *J* = 7.3 Hz, 9H); ^13^C NMR (126 MHz, CDCl_3_): δ 162.0, 158.3, 155.1, 150.0, 149.1, 143.5, 130.5, 128.3, 126.1, 123.9, 112.4, 109.3, 100.3, 56.2, 49.1, 31.4 (q, *J* = 29.0 Hz), 29.3, 27.6, 25.8, 21.7, 13.8, 13.7, 10.0, and 8.7 (d, *J* = 8.4 Hz); ^19^F NMR (376 MHz, CDCl_3_): δ -66.1 (t, *J* = 10.8 Hz).

**8-(4-Bromo-2,6-dimethoxyphenyl)-2,7-dimethyl-*N,N*-bis(4,4,4-trifluorobutyl)pyrazolo[1,5-α][1,3,5]triazin-4-amine (BMK-C203).** Compound BMK-C203 was prepared per the procedure described for synthesizing BMK-C201. The title compound was obtained (47.9 mg, 40% yield) as a pale yellow solid. TLC (n-Hex:EtOAc, 7:1 v/v): *R*_f_ = 0.12; ^1^H NMR (400 MHz, CDCl_3_): δ 6.80 (s, 2H), 4.04 (s, 4H), 3.75 (s, 6H), 2.38 (s, 3H), 2.29–2.19 (m, 4H), 2.18 (d, *J* = 5.7 Hz, 3H), and 2.12–2.02 (m, *J* = 14.8, 7.5 Hz, 4H); ^13^C NMR (126 MHz, CDCl_3_): δ 162.3, 159.3, 154.8, 149.9, 149.0, 130.4, 128.2, 126.0, 123.8, 122.6, 108.4, 108.2, 107.9, 99.0, 67.7, 60.5, 56.2, 49.1, 31.3 (q, *J* = 29.2 Hz), 29.3, 25.8, 24.0, 21.6, 14.3, and 13.6; ^19^F NMR (376 MHz, CDCl_3_): δ -66.1 (t, *J* = 10.8 Hz). HRMS (m/z): ([M]+ calculated for C_23_H_26_BrF_6_N_5_O_2_ = 598.1247) 598.1240 (found).

**8-(4-(Benzyloxy)-2,6-dimethoxyphenyl)-2,7-dimethyl-*N,N*-bis(3,3,3-trifluoropropyl)pyrazolo[1,5-α][1,3,5]triazin-4-amine (2d).** Compound 2d was prepared per the procedure described for synthesizing 2a. The title compound was obtained (490 mg, 82% yield) as a white solid. TLC (n-Hex:EtOAc, 4:1 v/v): *R*_f_ = 0.25; ^1^H NMR (400 MHz, CDCl_3_): δ 7.49–7.45 (m, 2H), 7.44–7.39 (m, 2H), 7.38–7.32 (m, 1H), 6.32 (s, 2H), 5.10 (s, 2H), 4.15 (m, 4H), 3.73 (s, 6H), 2.74 (m, 4H), 2.41 (s, 3H), and 2.22 (s, 3H); ^13^C NMR (101 MHz, CDCl_3_): δ 161.9, 160.7, 159.6, 155.9, 149.7, 148.7, 137.0, 128.8, 128.3, 127.8, 127.6, 124.8, 101.6, 100.3, 92.3, 70.4, 56.1, 45.0, 25.8, and 13.6; ^19^F NMR (376 MHz, CDCl_3_): δ -65.2 (t, *J* = 10.8 Hz).

**4-(4-(Bis(3,3,3-trifluoropropyl)amino)-2,7-dimethylpyrazolo[1,5-α][1,3,5]triazin-8-yl)-3,5-dimethoxyphenol (3d).** Compound 3d was prepared per the procedure described for synthesizing 3a. The title compound was obtained (447 mg, 88% yield) as a white solid. The filtrate was concentrated under reduced pressure to produce a white solid (1.57 g, 88% yield). TLC (n-Hex:EtOAc, 4:1 v/v): *R*_f_ = 0.28; ^1^H NMR (400 MHz, CDCl_3_): δ 10.15 (br, s, 1H), 5.84 (s, 2H), 4.21 (br, s, 4H), 3.60 (s, 6H), 2.77 (m, 4H), 2.52 (s, 3H), and 2.19 (s, 3H); ^13^C NMR (101 MHz, CDCl_3_): δ 162.2, 159.6, 159.1, 156.8, 148.9, 148.3, 127.5, 124.8, 101.3, 97.7, 93.0, 55.5, 45.2, 24.7, and 13.5; ^19^F NMR (376 MHz, CDCl­_3_): δ -65.1 (t, *J* = 10.8 Hz).

**4-(4-(Bis(3,3,3-trifluoropropyl)amino)-2,7-dimethylpyrazolo[1,5-α][1,3,5]triazin-8-yl)-3,5-dimethoxyphenyl trifluoromethanesulfonate (4d).** Compound 4d was prepared per the procedure described for synthesizing 4a. The title compound was obtained (435 mg, 68% yield) as a white solid. TLC (n-Hex:EtOAc, 7:1 v/v): *R*_f_ = 0.36; ^1^H NMR (400 MHz, CDCl_3_): δ 6.55 (s, 2H), 4.18 (br, s, 4H), 3.77 (s, 6H), 2.74 (m, 4H), 2.42 (s, 3H), and 2.20 (s, 3H); ^13^C NMR (101 MHz, CDCl_3_): δ 162.5, 159.5, 155.5, 150.5, 148.6, 130.3, 127.5, 124.8, 120.5, 117.3, 109.1, 98.9, 98.3, 56.4, 45.0 (d, *J* = 3.6 Hz), 25.8, and 13.6; ^19^F NMR (376 MHz, CDCl_3_): δ -65.2 (t, *J* = 10.8 Hz), -72.9.

**8-(2,6-Dimethoxy-4-(tributylstannyl)phenyl)-2,7-dimethyl-*N,N*-bis(3,3,3-trifluoropropyl)pyrazolo[1,5-α][1,3,5]triazin-4-amine (5d).** Compound 5d was prepared per the procedure described for synthesizing 5a. The title compound was obtained (211 mg, 54% yield) as a white solid. TLC (n-Hex:EtOAc, 7:1 v/v): *R*_f_ = 0.57; ^1^H NMR (400 MHz, CDCl_3_): δ 6.73 (s, 2H), 4.17 (br, s, 4H), 3.77 (s, 6H), 2.74 (m, 4H), 2.41 (s, 3H), 2.23 (s, 3H), 1.58 (m, 6H), 1.38 (m, 6H), 1.08 (m, 6H), and 0.94 (m, 9H); ^13^C NMR (101 MHz, CDCl_3_): δ 162.0, 158.1, 155.8, 149.7, 148.7, 143.7, 132.5, 130.4, 127.6, 124.9, 112.2, 108.8, 100.6, 56.2, 45.0 (d, *J* = 3.6 Hz), 29.5–29.1 (m), 27.6, 25.9, 13.8 (d, *J* = 12.1 Hz), 11.6, 10.0, and 8.3 (d, *J* = 7.6 Hz); ^19^F NMR (376 MHz, CDCl_3_): δ -65.2 (t, *J* = 10.8 Hz).

**8-(4-Bromo-2,6-dimethoxyphenyl)-2,7-dimethyl-*N*,*N*-bis(3,3,3-trifluoropropyl)pyrazolo[1,5-α][1,3,5]triazin-4-amine (BMK-C204).** Compound BMK-C204 was prepared per the procedure described for synthesizing BMK-C201. The title compound was obtained (105 mg, 92% yield) as a white solid. TLC (n-Hex:EtOAc, 7:1 v/v): *R*_f_ = 0.14; ^1^H NMR (400 MHz, CDCl_3_): δ 6.80 (s, 2H), 4.17 (br, s, 4H), 3.75 (s, 6H), 2.74 (m, 4H), 2.41 (s, 3H), and 2.20 (s, 3H); ^13^C NMR (101 MHz, CDCl_3_): δ 162.3, 159.3, 155.5, 149.8, 148.6, 130.3, 127.6, 124.8, 122.8, 108.5, 108.0, 99.5, 56.3, 45.0 (d, *J* = 3.8 Hz), 25.8, and 13.6; ^19^F NMR (376 MHz, CDCl_3_): δ -65.2 (t, *J* = 10.8 Hz). HRMS (m/z): ([M]^+^ calculated for C_21_H_22_BrF_6_N_5_O_2_ = 570.0934) 570.0931 (found).

**8-(4-(Benzyloxy)-2,6-dimethoxyphenyl)-*N*-butyl-*N*-(cyclopropylmethyl)-2,7-dimethylpyrazolo[1,5-α][1,3,5]triazin-4-amine (2e).** 8-(4-Benzyloxy-2,6-dimethoxyphenyl)-4-chloro-2,7-dimethylpyrazolo-[1,5-α][1,3,5]triazine (1) (424 mg, 1.0 mmol) was dissolved in dry MeOH (5 mL). *N*-(cyclopropylmethyl)butan-1-aminium chloride (196 mg, 1.2 mmol) and dry triethylamine (0.28 mL, 2.0 mmol) were added via a syringe. The reaction mixture was heated to the reflux temperature under argon for 3 h. The reaction mixture was cooled to room temperature and extracted with ethyl acetate (3 × 10 mL), dried over MgSO_4_, filtered and concentrated in vacuo. The crude product was purified via silica gel flash chromatography. The title compound 2e was obtained (371 mg, 72% yield) as a white solid. TLC (n-Hex:EtOAc, 2:1 v/v): *R*_f_ = 0.45; ^1^H NMR (400 MHz, CDCl_3_): δ 7.48 (d, *J* = 7.4 Hz, 2H), 7.42 (t, *J* = 7.4 Hz, 2H), 7.35 (t, *J* = 7.1 Hz, 1H), 6.33 (s, 2H), 5.11 (s, 2H), 4.14 (s, 2H), 3.95 (s, 2H), 3.73 (s, 6H), 2.39 (s, 3H), 2.22 (s, 3H), 1.85–1.72 (m, 2H), 1.50–1.35 (m, 2H), 1.27 (t, *J* = 6.6 Hz, 1H), 0.99 (t, *J* = 7.3 Hz, 3H), 0.55 (d, *J* = 7.7 Hz, 2H), and 0.39 (d, *J* = 4.7 Hz, 2H); ^13^C NMR (126 MHz, CDCl_3_): δ 161.8, 160.4, 159.7, 154.3, 150.2, 149.1, 137.2, 128.7, 128.1, 127.7, 102.6, 99.3, 92.6, 70.4, 56.1, 54.0, 50.0, 31.0, 25.9, 20.2, 14.0, 13.6, 10.5, and 3.8.

**4-(4-(Butyl(cyclopropylmethyl)amino)-2,7-dimethylpyrazolo[1,5-α][1,3,5]triazin-8-yl)-3,5-dimethoxyphenol (3e).** Compound 3e was prepared per the procedure described for synthesizing 3a. The title compound was obtained (374 mg, 88% yield) as a white solid. TLC (n-Hex:EtOAc, 1:1 v/v): *R*_f_ = 0.24; ^1^H NMR (400 MHz, CDCl_3_): δ 5.85 (s, 2H), 4.30–3.81 (m, 4H), 3.60 (s, 6H), 2.49 (s, 3H), 2.20 (s, 3H), 1.88–1.74 (m, 2H), 1.49–1.39 (m, *J* = 14.7, 7.4 Hz, 2H), 1.34–1.26 (m, 1H), 1.01 (t, *J* = 7.3 Hz, 3H), 0.58 (d, *J* = 7.6 Hz, 2H), and 0.43 (d, *J* = 5.0 Hz, 2H); ^13^C NMR (101 MHz, CDCl_3_): δ 161.9, 159.7, 159.0, 155.2, 149.1, 148.6, 100.3, 98.1, 93.1, 55.4 (d, *J* = 2.1 Hz), 54.3, 50.3, 44.9, 39.6, 32.0, 24.5, 20.2, 14.8, 14.0, 13.5, 10.4, 7.1, and 3.8.

**4-(4-(Butyl(cyclopropylmethyl)amino)-2,7-dimethylpyrazolo[1,5-α][1,3,5]triazin-8-yl)-3,5-dimethoxyphenyl trifluoromethanesulfonate (4e).** Compound 4e was prepared per the procedure described for synthesizing 4a. The title compound was obtained (307 mg, 55% yield) as a white solid. TLC (n-Hex:EtOAc, 7:1 v/v): *R*_f_ = 0.16; ^1^H NMR (400 MHz, CDCl_3_): δ 6.54 (s, 2H), 4.12 (d, *J* = 7.1 Hz, 2H), 3.94 (s, 2H), 3.76 (s, 6H), 2.38 (s, 3H), 2.19 (s, 3H), 1.83–1.72 (m, 3H), 1.46–1.35 (m, 2H), 1.32–1.21 (m, 1H), 0.99 (t, *J* = 7.4 Hz, 3H), 0.59–0.51 (m, 2H), and 0.43–0.36 (m, *J* = 5.0 Hz, 2H); ^13^C NMR (126 MHz, CDCl_3_): δ 162.4, 159.5, 153.9, 150.3, 149.0, 120.2, 117.6, 115.1, 109.9, 104.2, 98.3, 97.8, 56.4, 54.2, 50.2, 25.9, 20.2, 14.3, 14.0, 13.6, 10.4, and 3.8; ^19^F NMR (376 MHz, CDCl_3_): δ -72.9.

***N*-Butyl-*N*-(cyclopropylmethyl)-8-(2,6-dimethoxy-4-(tributylstannyl)phenyl)-2,7-dimethylpyrazolo[1,5-α][1,3,5]triazin-4-amine (5e).** Compound 5e was prepared per the procedure described for synthesizing 5a. The title compound was obtained (168 mg, 48% yield) as a white solid. TLC (n-Hex:EtOAc, 7:1 v/v): *R*_f_ = 0.48; ^1^H NMR (500 MHz, CDCl_3_): δ 6.73 (s, 2H), 4.04 (d, *J* = 96.4 Hz, 4H), 3.77 (s, 6H), 2.38 (s, 3H), 2.23 (s, 3H), 1.83–1.73 (m, 2H), 1.69–1.52 (m, *J* = 15.2, 7.6 Hz, 6H), 1.45–1.33 (m, *J* = 14.1, 6.9 Hz, 8H), 1.18–1.03 (m, 6H), 0.99 (t, *J* = 7.1 Hz, 3H), and 0.93 (t, *J* = 7.0 Hz, 9H); ^13^C NMR (126 MHz, CDCl_3_): δ 161.9, 158.2, 154.2, 150.1, 149.1, 143.0, 112.3, 109.7, 99.5, 56.2 (d, *J* = 5.5 Hz), 54.1, 50.0, 30.9, 29.2, 27.6, 25.9 (d, *J* = 2.8 Hz), 20.2, 14.0, 13.9, 11.2 (d, *J* = 7.5 Hz), 10.5, 9.9, 8.6 (d, *J* = 7.5 Hz), and 3.8.

**8-(4-Bromo-2,6-dimethoxyphenyl)-*N*-butyl-*N*-(cyclopropylmethyl)-2,7-dimethylpyrazolo[1,5-α][1,3,5]triazin-4-amine (BMK-C205).** Compound BMK-C205 was prepared per the procedure described for synthesizing BMK-C201. The title compound was obtained (60.6 mg, 62% yield) as a white solid. TLC (n-Hex:EtOAc, 7:1 v/v): *R*_f_ = 0.20; ^1^H NMR (400 MHz, CDCl_3_): δ 6.79 (s, 2H), 4.19–4.08 (m, *J* = 13.9, 6.9 Hz, 2H), 4.00–3.87 (m, *J* = 7.3 Hz, 1H), 2.37 (s, 3H), 2.19 (s, 3H), 1.82–1.71 (m, 2H), 1.45–1.35 (m, 2H), 1.30–1.21 (m, 1H), 0.98 (t, *J* = 7.4 Hz, 3H), 0.58–0.50 (m, 2H), and 0.43–0.35 (m, *J* = 4.9 Hz, 2H); ^13^C NMR (126 MHz, CDCl_3_): δ 162.2, 159.5, 153.9, 150.3, 149.1, 122.4, 108.9, 108.6, 98.4, 56.3 (d, *J* = 2.4 Hz), 54.1, 50.1, 31.0, 25.9, 20.2, 14.3, 14.0, 13.6, 10.5, and 3.8; HRMS (m/z): ([M]^+^ calculated for C_23_H_30_BrN_5_O_2_ = 488.1656) 488.1651 (found).

**8-(4-(Benzyloxy)-2,6-dimethoxyphenyl)-*N,N*-bis(2-ethoxyethyl)-2,7-dimethylpyrazolo[1,5-α][1,3,5]triazin-4-amine (2f).** Compound 2f was prepared per the procedure described for synthesizing 2a. The title compound was obtained (434 mg, 79% yield) as a white solid. TLC (n-Hex:EtOAc, 2:1 v/v): *R*_f_ = 0.28; ^1^H NMR (500 MHz, CDCl_3_): δ 7.48 (d, *J* = 6.5 Hz, 2H), 7.42 (t, *J* = 6.8 Hz, 2H), 7.36 (d, *J* = 6.6 Hz, 1H), 6.33 (s, 2H), 5.11 (s, 2H), 4.31 (s, 4H), 3.81 (s, 4H), 3.73 (s, 6H), 3.55 (d, *J* = 6.7 Hz, 4H), 2.39 (s, 3H), 2.21 (s, 3H), and 1.22 (t, *J* = 6.6 Hz, 6H); ^13^C NMR (126 MHz, CDCl_3_): δ 161.8, 160.4, 159.6, 154.6, 149.9, 149.1, 137.1, 128.7, 128.1, 127.7, 102.2, 99.4, 92.3, 70.3, 69.7, 66.6, 56.0 (d, *J* = 4.3 Hz), 51.2, 29.8, 25.8, 15.4, and 13.6.

**4-(4-(Bis(2-ethoxyethyl)amino)-2,7-dimethylpyrazolo[1,5-α][1,3,5]triazin-8-yl)-3,5-dimethoxyphenol (3f).** Compound 3f was prepared per the procedure described for synthesizing 3a. The title compound was obtained (413 mg, 90% yield) as a white solid. TLC (n-Hex:EtOAc, 1:1 v/v): *R*_f_ = 0.19; ^1^H NMR (500 MHz, CDCl_3_): δ 5.84 (s, 2H), 4.35 (s, 4H), 3.83 (s, 4H), 3.59 (s, 6H), 3.55 (d, *J* = 6.9 Hz, 4H), 2.48 (s, 3H), 2.17 (s, 3H), and 1.21 (t, *J* = 6.8 Hz, 6H); ^13^C NMR (126 MHz, CDCl_3_): δ 162.0, 159.7, 159.0, 155.5, 149.2, 148.4, 100.4, 97.9, 93.1, 66.7, 55.4 (d, *J* = 5.3 Hz), 51.5, 24.6 (d, *J* = 3.2 Hz), 15.4, and 13.5.

**4-(4-(Bis(2-ethoxyethyl)amino)-2,7-dimethylpyrazolo[1,5-α][1,3,5]triazin-8-yl)-3,5-dimethoxyphenyl trifluoromethanesulfonate (4f).** Compound 4f was prepared per the procedure described for synthesizing 4a. The title compound was obtained (343 mg, 68% yield) as a white solid. TLC (n-Hex:EtOAc, 2:1 v/v): *R*_f_ = 0.38; ^1^H NMR (500 MHz, CDCl_3_): δ 6.54 (s, 2H), 4.30 (s, 4H), 3.80 (s, 4H), 3.76 (s, 6H), 3.54 (q, *J* = 6.7 Hz, 4H), 2.38 (s, 3H), 2.17 (s, 3H), and 1.20 (t, *J* = 6.8 Hz, 6H); ^13^C NMR (126 MHz, CDCl_3_): δ 162.4, 159.5, 154.2, 150.3, 150.1, 149.1, 120.2, 117.6, 109.7, 98.3, 98.0, 69.7, 66.6, 56.4 (d, *J* = 4.6 Hz), 51.3, 29.8, 25.8, 15.4, and 13.6; ^19^F NMR (376 MHz, CDCl_3_): δ -72.9.

**8-(2,6-Dimethoxy-4-(tributylstannyl)phenyl)-*N*,*N*-bis(2-ethoxyethyl)-2,7-dimethylpyrazolo[1,5-α][1,3,5]triazin-4-amine (5f).** Compound 5f was prepared per the procedure described for synthesizing 5a. The title compound was obtained (187 mg, 51% yield) as a white solid. TLC (n-Hex:EtOAc, 5:1 v/v): *R*_f_ = 0.14; ^1^H NMR (500 MHz, CDCl_3_): δ 6.72 (s, 2H), 4.29 (s, 4H), 3.80 (s, 4H), 3.76 (s, 6H), 3.54 (d, *J* = 5.9 Hz, 4H), 2.37 (s, 3H), 2.21 (s, 3H), 1.68–1.49 (m, *J* = 14.9, 7.7 Hz, 6H), 1.37 (d, *J* = 6.6 Hz, 6H), 1.20 (s, 6H), 1.08 (t, *J* = 6.5 Hz, 6H), and 0.96–0.85 (m, 9H); ^13^C NMR (126 MHz, CDCl_3_): δ 161.9, 158.2, 154.5, 150.0, 149.1, 143.2, 112.3, 109.4, 99.7, 69.8, 66.6, 56.2 (d, *J* = 4.8 Hz), 51.3, 29.3 (d, *J* = 10.0 Hz), 27.6, 27.4, 25.9, 15.4, 13.9, 13.8, 11.3, 9.9, and 8.6.

**8-(4-Bromo-2,6-dimethoxyphenyl)-*N*,*N*-bis(2-ethoxyethyl)-2,7-dimethylpyrazolo[1,5-α][1,3,5]triazin-4-amine (BMK-C206).** Compound BMK-C206 was prepared per the procedure described for synthesizing BMK-C201. The title compound was obtained (65.8 mg, 63% yield) as a white solid. TLC (n-Hex:EtOAc, 5:1 v/v): *R*_f_ = 0.12; ^1^H NMR (500 MHz, CDCl_3_): δ 6.79 (s, 2H), 4.29 (s, 4H), 3.79 (s, 4H), 3.73 (s, 6H), 3.53 (q, *J* = 6.6 Hz, 4H), 2.37 (s, 3H), 2.17 (s, 3H), and 1.19 (t, *J* = 6.7 Hz, 6H); ^13^C NMR (126 MHz, CDCl_3_): δ 162.1, 159.3, 154.1, 150.0, 149.0, 122.4, 108.5, 108.4, 98.5, 69.6, 66.5, 56.2 (d, *J* = 4.2 Hz), 51.2, 25.8, 15.3, and 13.5; HRMS (m/z): ([M]^+^ calculated for C_23_H_32_BrN_5_O_4_ = 522.1710) 522.1707 (found).

**8-(4-(Benzyloxy)-2,6-dimethoxyphenyl)-*N*-butyl-2,7-dimethyl-*N*-(4,4,4-trifluorobutyl)pyrazolo[1,5-α][1,3,5]triazin-4-amine (2g).** Compound 2g was prepared per the procedure described for synthesizing 2a. The title compound was obtained (434 mg, 76% yield) as a white solid. TLC (n-Hex:EtOAc, 2:1 v/v): *R*_f_ = 0.46; ^1^H NMR (500 MHz, CDCl_3_): δ 7.47 (d, *J* = 7.3 Hz, 2H), 7.41 (t, *J* = 7.0 Hz, 2H), 7.35 (d, *J* = 7.0 Hz, 1H), 6.31 (s, 2H), 5.10 (s, 2H), 4.00 (d, *J* = 58.5 Hz, 4H), 3.72 (s, 6H), 3.48 (s, 4H), 2.36 (s, 3H), 2.28–2.16 (m, 5H), 2.13–2.05 (m, 2H), 1.78–1.71 (m, *J* = 9.1, 6.0 Hz, 2H), 1.44–1.35 (m, *J* = 14.1, 7.6 Hz, 2H), and 0.98 (t, *J* = 7.4 Hz, 3H); ^13^C NMR (126 MHz, CDCl_3_): δ 171.3, 162.0, 160.5, 159.6, 154.8, 150.0, 149.0, 137.1, 128.8, 128.3 (d, *J* = 19.6 Hz), 127.8, 126.2, 102.2, 99.4, 92.4, 70.4, 60.6, 56.1 (d, *J* = 3.9 Hz), 50.3, 49.0, 31.5, 31.3, 29.9, 25.8, 21.2, 20.2, 14.3, 14.0, and 13.6; ^19^F NMR (376 MHz, CDCl_3_): δ -66.1 (t, *J* = 10.8 Hz).

**4-(4-(Butyl(4,4,4-trifluorobutyl)amino)-2,7-dimethylpyrazolo[1,5-α][1,3,5]triazin-8-yl)-3,5-dimethoxyphenol (3g).** Compound 3g was prepared per the procedure described for synthesizing 3a. The title compound was obtained (419 mg, 87% yield) as a white solid. TLC (n-Hex:EtOAc, 2:1 v/v): *R*_f_ = 0.11; ^1^H NMR (400 MHz, CDCl_3_): δ 5.86 (s, 2H), 3.60 (s, 6H), 2.48 (s, 3H), 2.31–2.20 (m, 2H), 2.18 (s, 3H), 2.16–2.03 (m, 2H), 1.86–1.69 (m, 2H), 1.50–1.37 (m, 2H), and 1.01 (t, *J* = 7.3 Hz, 3H); ^13^C NMR (126 MHz, CDCl_3_): δ 162.1, 159.7, 159.0, 155.7, 149.0, 148.4, 130.5, 128.3, 126.1, 123.9, 100.5, 97.9, 93.1 (d, *J* = 6.9 Hz), 60.5, 55.4 (d, *J* = 7.8 Hz), 50.6, 49.2, 31.4 (q), 24.6 (d, *J* = 5.6 Hz), 20.2, 14.0, and 13.5 (d, *J* = 3.4 Hz); ^19^F NMR (376 MHz, CDCl_3_): δ -66.0 (t, *J* = 10.5 Hz) and -75.6.

**4-(4-(Butyl(4,4,4-trifluorobutyl)amino)-2,7-dimethylpyrazolo[1,5-α][1,3,5]triazin-8-yl)-3,5-dimethoxyphenyl trifluoromethanesulfonate (4g).** Compound 4g was prepared per the procedure described for synthesizing **4a**. The title compound was obtained (344 mg, 56% yield) as a white solid. TLC (n-Hex:EtOAc, 5:1 v/v): *R*_f_ = 0.12; ^1^H NMR (400 MHz, CDCl_3_): δ 6.57 (s, 2H), 4.16–3.89 (m, *J* = 56.5, 12.5 Hz, 4H), 3.76 (s, 6H), 2.39 (s, 3H), 2.31–2.22 (m, *J* = 9.4, 6.8 Hz, 2H), 2.20 (s, 3H), 2.16–2.04 (m, 2H), 1.82–1.72 (m, 2H), 1.48–1.36 (m, 2H), and 1.00 (t, *J* = 7.4 Hz, 3H); ^13^C NMR (101 MHz, CDCl_3_): δ 162.4, 159.5, 154.2, 150.3, 150.1, 148.9, 131.3, 128.6, 125.8, 123.6, 123.1, 120.4, 117.2, 114.1, 109.7, 98.2, 98.0, 56.2, 50.3, 49.0, 31.2 (q, *J* = 29.2 Hz), 25.6, 20.0, 13.8, and 13.4; ^19^F NMR (376 MHz, CDCl_3_): δ -66.2 (t, *J* = 10.7 Hz) and -73.1.

***N*-Butyl-8-(2,6-dimethoxy-4-(tributylstannyl)phenyl)-2,7-dimethyl-*N*-(4,4,4-trifluorobutyl)pyrazolo[1,5-α][1,3,5]triazin-4-amine (5g).** Compound 5g was prepared per the procedure described for synthesizing 5a. The title compound was obtained (181 mg, 48% yield) as a white solid. TLC (n-Hex:EtOAc, 7:1 v/v): *R*_f_ = 0.45; ^1^H NMR (400 MHz, CDCl_3_): δ 6.73 (s, 2H), 4.01 (d, *J* = 48.3 Hz, 4H), 3.78 (s, 6H), 2.38 (s, 3H), 2.31–2.16 (m, 5H), 2.14–2.03 (m, *J* = 14.4, 7.5 Hz, 2H), 1.81–1.70 (m, 2H), 1.68–1.53 (m, 6H), 1.47–1.32 (m, 8H), 1.17–1.04 (m, 6H), 1.00 (t, *J* = 7.4 Hz, 3H), and 0.93 (t, *J* = 7.3 Hz, 9H); ^13^C NMR (126 MHz, CDCl_3_): δ 162.0, 158.2, 154.6, 150.0, 149.0, 143.2, 128.4, 126.2, 112.3, 109.4, 99.7, 56.2 (d, *J* = 4.2 Hz), 50.3, 49.0, 31.4 (q, *J* = 29.3 Hz), 29.3, 27.6, 25.9, 21.9, 20.2, 13.9 (t, *J* = 13.3 Hz), 11.3 (d, *J* = 7.3 Hz), 9.9, and 8.6 (d, *J* = 7.8 Hz); ^19^F NMR (376 MHz, CDCl_3_): δ -66.1 (t, *J* = 10.8 Hz).

**8-(4-Bromo-2,6-dimethoxyphenyl)-*N*-butyl-2,7-dimethyl-*N*-(4,4,4-trifluorobutyl)pyrazolo[1,5-α][1,3,5]triazin-4-amine (BMK-C207).** Compound BMK-C207 was prepared per the procedure described for synthesizing BMK-C201. The title compound was obtained (68.6 mg, 69% yield) as a white solid. TLC (n-Hex:EtOAc, 7:1 v/v): *R*_f_ = 0.14; ^1^H NMR (400 MHz, CDCl_3_): δ 6.79 (s, 2H), 4.25–3.87 (m, *J* = 59.4, 16.6 Hz, 4H), 3.74 (s, 6H), 2.38 (d, *J* = 4.7 Hz, 3H), 2.30–2.15 (m, 5H), 2.13–2.02 (m, 2H), 1.79–1.69 (m, 2H), 1.46–1.36 (m, 2H), and 0.99 (t, *J* = 7.4 Hz, 3H); ^13^C NMR (126 MHz, CDCl_3_): δ 162.3, 159.4, 154.3, 150.0, 148.9, 130.5, 128.3, 126.1, 122.5, 108.4, 98.6, 67.8, 56.5–55.4 (m), 50.4, 49.1, 31.4 (q, *J* = 28.9 Hz), 25.8, 20.1, 14.0, and 13.6; ^19^F NMR (376 MHz, CDCl_3_): δ -66.1 (t, *J* = 10.8 Hz); HRMS (m/z): ([M]^+^ calculated for C_23_H_29_BrF_3_N_5_O_2_ = 544.1530) 544.1537 (found).

**8-(4-(Benzyloxy)-2,6-dimethoxyphenyl)-2,7-dimethyl-*N*-(4,4,4-trifluorobutyl)-*N*-(3,3,3-trifluoropropyl)pyrazolo[1,5-α][1,3,5]triazin-4-amine (2h).** Compound 2h was prepared per the procedure described for synthesizing 2a. The title compound was obtained (404 mg, 66% yield) as a white solid. TLC (n-Hex:EtOAc, 5:1 v/v): *R*_f_ = 0.32; ^1^H NMR (500 MHz, CDCl_3_): δ 7.48 (d, *J* = 7.2 Hz, 2H), 7.42 (t, *J* = 7.4 Hz, 2H), 7.37 (d, *J* = 7.2 Hz, 1H), 6.34 (s, 2H), 5.11 (s, 2H), 4.20–4.00 (m, 4H), 3.74 (s, 6H), 2.79–2.64 (m, *J* = 20.5, 10.4 Hz, 2H), 2.41 (s, 3H), 2.29–2.20 (m, 5H), and 2.16–2.07 (m, 2H); ^13^C NMR (126 MHz, CDCl_3_): δ 161.9, 160.6, 159.6, 155.5, 149.8, 148.8, 137.1, 130.4, 129.5, 128.8, 128.2, 127.7, 127.3, 126.0, 125.1, 123.8, 122.9, 101.7, 100.1, 92.8–91.7 (m), 70.7–70.0 (m), 56.0 (td, *J* = 9.9, 3.5 Hz), 49.5, 44.6, 44.2, 31.3 (q, *J* = 29.4 Hz), 25.8 (d, *J* = 3.6 Hz), 21.7, and 13.6 (d, *J* = 2.7 Hz); ^19^F NMR (376 MHz, CDCl_3_): δ -65.2 (t, *J* = 10.9 Hz) and -66.1 (t, *J* = 10.7 Hz).

**4-(2,7-Dimethyl-4-((4,4,4-trifluorobutyl)(3,3,3-trifluoropropyl)amino)pyrazolo[1,5-α][1,3,5]triazin-8-yl)-3,5-dimethoxyphenol (3h).** Compound 3h was prepared per the procedure described for synthesizing 3a. The title compound was obtained (454 mg, 87% yield) as a white solid. TLC (n-Hex:EtOAc, 5:1 v/v): *R*_f_ = 0.14; ^1^H NMR (400 MHz, CDCl_3_): δ 5.84 (s, 2H), 4.19–4.03 (m, *J* = 14.3, 7.1 Hz, 4H), 3.60 (s, 6H), 2.83–2.66 (m, 2H), 2.51 (s, 3H), 2.36–2.22 (m, *J* = 17.3, 8.7 Hz, 2H), 2.19 (s, 3H), and 2.17–2.08 (m, 2H); ^13^C NMR (126 MHz, CDCl_3_): δ 162.2, 159.7, 159.0, 156.5, 149.0, 148.3, 130.4, 129.5, 128.2, 127.3, 126.0, 125.1, 123.8, 122.9, 101.1, 97.7, 93.1, 60.6, 55.4 (d, *J* = 4.7 Hz), 49.8, 44.5, 31.3 (q, *J* = 29.4 Hz), 29.8, 26.0, 24.6 (d, *J* = 3.0 Hz), 21.2, 14.3, and 13.4; ^19^F NMR (376 MHz, CDCl_3_): δ -65.2 (t, *J* = 10.8 Hz) and -66.1 (t, *J* = 10.6 Hz).

**4-(2,7-Dimethyl-4-((4,4,4-trifluorobutyl)(3,3,3-trifluoropropyl)amino)pyrazolo[1,5-α][1,3,5]triazin-8-yl)-3,5-dimethoxyphenyl trifluoromethanesulfonate (4h).** Compound 4h was prepared per the procedure described for synthesizing 4a. The title compound was obtained (346 mg, 53% yield) as a white solid. TLC (n-Hex:EtOAc, 5:1 v/v): *R*_f_ = 0.14; ^1^H NMR (400 MHz, CDCl_3_): δ 6.55 (s, 2H), 4.21–4.01 (m, 4H), 3.77 (s, 6H), 2.71 (dq, *J* = 20.8, 10.5 Hz, 2H), 2.40 (s, 3H), 2.32–2.17 (m, 5H), and 2.15–2.06 (m, *J* = 14.9, 7.6 Hz, 2H); ^13^C NMR (126 MHz, CDCl_3_): δ 162.5, 159.5, 155.1, 150.5, 150.0, 148.8, 128.2, 127.3, 126.0, 125.1, 120.2, 117.6, 109.2, 98.7, 98.3, 56.4, 49.7, 44.4, 31.3 (q, *J* = 58.9, 29.6 Hz), 25.8, and 13.6; ^19^F NMR (376 MHz, CDCl_3_): δ -65.3 (t, *J* = 10.8 Hz), -66.1 (t, *J* = 10.7 Hz), and -72.9.

**8-(2,6-Dimethoxy-4-(tributylstannyl)phenyl)-2,7-dimethyl-*N*-(4,4,4-trifluorobutyl)-*N*-(3,3,3-trifluoropropyl)pyrazolo[1,5-α][1,3,5]triazin-4-amine (5h).** Compound 5h was prepared per the procedure described for synthesizing 5a. The title compound was obtained (175 mg, 44% yield) as a white solid. TLC (n-Hex:EtOAc, 7:1 v/v): *R*_f_ = 0.31; ^1^H NMR (500 MHz, CDCl_3_): δ 6.74 (s, 2H), 4.11 (d, *J* = 26.0 Hz, 4H), 3.78 (s, 6H), 2.79–2.65 (m, *J* = 15.6, 10.1 Hz, 2H), 2.40 (s, 3H), 2.30–2.21 (m, 5H), 2.15–2.06 (m, 2H), 1.67–1.55 (m, 6H), 1.43–1.34 (m, 6H), 1.15–1.01 (m, 6H), and 0.93 (t, *J* = 7.3 Hz, 9H); ^13^C NMR (126 MHz, CDCl_3_): δ 162.0, 158.1, 155.4, 149.8, 148.8, 143.5, 130.4, 129.5, 128.2, 127.3, 126.0, 125.1, 112.2, 108.9, 100.3, 56.2 (dd, *J* = 16.8, 7.7 Hz), 49.5, 44.3, 31.3 (q, *J* = 29.4 Hz), 29.3, 27.6, 25.9 (d, *J* = 5.2 Hz), 21.7, 13.9, 13.8 (d, *J* = 3.8 Hz), 11.2 (d, *J* = 7.4 Hz), 9.9, and 8.6 (d, *J* = 7.6 Hz); ^19^F NMR (376 MHz, CDCl_3_): δ -65.3 (t, *J* = 10.8 Hz) and -66.1 (t, *J* = 10.7 Hz).

**8-(4-Bromo-2,6-dimethoxyphenyl)-2,7-dimethyl-*N*-(4,4,4-trifluorobutyl)-*N*-(3,3,3-trifluoropropyl)pyrazolo[1,5-α][1,3,5]triazin-4-amine (BMK-C208).** Compound BMK-C208 was prepared per the procedure described for synthesizing BMK-C201. The title compound was obtained (67.8 mg, 58% yield) as a white solid. TLC (n-Hex:EtOAc, 7:1 v/v): *R*_f_ = 0.14; ^1^H NMR (400 MHz, CDCl_3_): δ 6.80 (s, 2H), 4.20–4.01 (m, *J* = 14.3, 7.1 Hz, 4H), 3.75 (s, 6H), 2.81–2.63 (m, 2H), 2.39 (s, 3H), 2.32–2.17 (m, 5H), and 2.15–2.06 (m, *J* = 15.0, 8.0 Hz, 2H); ^13^C NMR (101 MHz, CDCl_3_): δ 162.3, 159.3, 155.1, 149.9, 148.8, 128.5, 127.6, 125.7, 124.8, 122.7, 108.5, 108.1, 99.3, 56.3 (d, *J* = 2.4 Hz), 49.6, 44.3, 31.3 (q, *J* = 29.3 Hz), 29.9, 25.8, and 13.6; ^19^F NMR (376 MHz, CDCl_3_): δ -65.2 (t, *J* = 10.8 Hz) and -66.1; HRMS (m/z): ([M]^+^ calculated for C_23_H_29_BrF_3_N_5_O_2_ = 584.1090) 584.1090 (found).

**8-(4-(Benzyloxy)-2,6-dimethoxyphenyl)-*N*-(cyclopropylmethyl)-2,7-dimethyl-*N*-(4,4,4-trifluorobutyl)pyrazolo[1,5-α][1,3,5]triazin-4-amine (2i).** Compound 2i was prepared per the procedure described for synthesizing 2a. The title compound was obtained (399 mg, 70% yield) as a white solid. TLC (n-Hex:EtOAc, 2:1 v/v): *R*_f_ = 0.38; ^1^H NMR (500 MHz, CDCl_3_): δ 7.48 (d, *J* = 7.4 Hz, 2H), 7.42 (t, *J* = 7.5 Hz, 2H), 7.36 (t, *J* = 7.3 Hz, 1H), 6.33 (s, 2H), 5.11 (s, 2H), 4.21 (s, 2H), 3.92 (s, 2H), 3.73 (s, 6H), 2.40 (s, 3H), 2.23 (s, 5H), 2.17–2.07 (m, *J* = 14.8, 7.6 Hz, 2H), 1.30–1.22 (m, 1H), 0.63–0.53 (m, 2H), and 0.40 (q, *J* = 5.0 Hz, 2H); ^13^C NMR (126 MHz, CDCl_3_): δ 161.9, 160.5, 159.6, 154.7, 150.0, 149.1, 137.1, 128.7, 128.2, 127.7, 102.1, 99.6, 92.3, 70.4, 56.0, 54.3, 48.9, 31.4 (q, *J* = 29.0 Hz), 25.8, 13.6, 10.3, 7.0, and 3.7 (d, *J* = 19.3 Hz); ^19^F NMR (376 MHz, CDCl_3_): δ -66.0 (t, *J* = 10.8 Hz).

**4-(4-((Cyclopropylmethyl)(4,4,4-trifluorobutyl)amino)-2,7-dimethylpyrazolo[1,5-α][1,3,5]triazin-8-yl)-3,5-dimethoxyphenol (3i).** Compound 3i was prepared per the procedure described for synthesizing 3a. The title compound was obtained (388 mg, 81% yield) as a white solid. TLC (n-Hex:EtOAc, 2:1 v/v): *R*_f_ = 0.11; ^1^H NMR (400 MHz, CDCl_3_): δ 5.85 (s, 2H), 4.25 (s, 2H), 3.96 (s, 2H), 3.60 (s, 6H), 2.50 (s, 3H), 2.34–2.08 (m, 7H), 1.28 (dd, *J* = 7.3, 3.5 Hz, 1H), 0.66–0.55 (m, 2H), and 0.43 (q, *J* = 4.9 Hz, 2H); ^13^C NMR (101 MHz, CDCl_3_): δ 162.1, 159.7, 159.1, 155.7, 149.2, 148.5, 128.6, 125.9, 100.6, 98.0, 93.1, 55.4 (d, *J* = 2.1 Hz), 54.6, 49.217 (s), 31.41 (q, *J* = 29.1 Hz), 24.57 (s), 13.45 (s), 10.35 (s), and 3.90 (s); ^19^F NMR (376 MHz, CDCl_3_): δ -65.96 (t, *J* = 10.6 Hz).

**4-(4-((Cyclopropylmethyl)(4,4,4-trifluorobutyl)amino)-2,7-dimethylpyrazolo[1,5-α][1,3,5]triazin-8-yl)-3,5-dimethoxyphenyl trifluoromethanesulfonate (4i).** Compound 4i was prepared per the procedure described for synthesizing 4a. The title compound was obtained (300 mg, 49% yield) as a white solid. TLC (n-Hex:EtOAc, 5:1 v/v): *R*_f_ = 0.26; ^1^H NMR (400 MHz, CDCl_3_): δ 6.55 (s, 2H), 4.20 (s, 2H), 3.92 (s, 2H), 3.76 (s, 6H), 2.39 (s, 3H), 2.33–2.18 (m, 5H), 2.13 (dd, *J* = 14.8, 7.6 Hz, 2H), 1.26 (t, *J* = 7.1 Hz, 1H), 0.58 (q, *J* = 5.5 Hz, 2H), and 0.40 (q, *J* = 4.9 Hz, 2H); ^13^C NMR (101 MHz, CDCl_3_): δ 162.3, 159.4, 154.1, 150.2, 150.0, 148.9, 128.5, 125.7, 120.3, 117.1, 109.5, 98.1, 98.0, 56.2 (d, *J* = 2.6 Hz), 54.2, 48.8, 31.2 (q, *J* = 29.2 Hz), 25.7, 21.8, 14.1, 13.4, 10.2, and 3.7; ^19^F NMR (376 MHz, CDCl_3_): δ -66.0 (t, *J* = 10.8 Hz) and -72.9.

***N*-(Cyclopropylmethyl)-8-(2,6-dimethoxy-4-(tributylstannyl)phenyl)-2,7-dimethyl-*N*-(4,4,4-trifluorobutyl)pyrazolo[1,5-α][1,3,5]triazin-4-amine (5i).** Compound 5i was prepared per the procedure described for synthesizing 5a. The title compound was obtained (188 mg, 50% yield) as a white solid. TLC (n-Hex:EtOAc, 7:1 v/v): *R*_f_ = 0.45; ^1^H NMR (500 MHz, CDCl_3_): δ 6.73 (s, 2H), 4.20 (s, 2H), 3.91 (s, 2H), 3.77 (s, 6H), 2.38 (s, 3H), 2.30–2.19 (m, 5H), 2.16–2.07 (m, *J* = 15.0, 7.5 Hz, 2H), 1.65–1.54 (m, 6H), 1.44–1.33 (m, 6H), 1.28–1.23 (m, 1H), 1.16–1.00 (m, 6H), 0.93 (t, *J* = 7.3 Hz, 9H), 0.60–0.54 (m, 2H), and 0.40 (q, *J* = 4.9 Hz, 2H); ^13^C NMR (126 MHz, CDCl_3_): δ 162.0, 158.2, 154.7, 150.0, 149.2, 143.3, 130.6, 128.4, 126.2, 112.3, 109.4, 99.9, 56.2, 54.3, 48.9, 31.4 (q, *J* = 29.0 Hz), 29.5–29.1 (m), 28.0–27.2 (m), 25.9, 13.9, 13.8, 11.3 (d, *J* = 7.3 Hz), 10.4, 9.9, 8.6 (d, *J* = 7.6 Hz), and 3.9; ^19^F NMR (376 MHz, CDCl_3_): δ -66.0 (t, *J* = 10.8 Hz).

**8-(4-Bromo-2,6-dimethoxyphenyl)-*N*-(cyclopropylmethyl)-2,7-dimethyl-*N*-(4,4,4-trifluorobutyl)pyrazolo[1,5-α][1,3,5]triazin-4-amine (BMK-C209).** Compound BMK-C209 was prepared per the procedure described for synthesizing BMK-C201. The title compound was obtained (57.5 mg, 53% yield) as a white solid. TLC (n-Hex:EtOAc, 7:1 v/v): *R*_f_ = 0.11; ^1^H NMR (499 MHz, CDCl_3_): δ 6.79 (s, 2H), 4.20 (s, 2H), 3.92 (s, 2H), 3.74 (s, 6H), 2.38 (s, 3H), 2.28–2.20 (m, 2H), 2.19 (s, 3H), 2.16–2.07 (m, *J* = 15.3, 7.7 Hz, 2H), 1.22 (s, 1H), 0.57 (q, *J* = 5.7 Hz, 2H), and 0.39 (q, *J* = 5.0 Hz, 2H); ^13^C NMR (126 MHz, CDCl_3_): δ 162.3, 159.3, 154.4, 150.0, 149.1, 130.6, 128.4, 126.2, 122.5, 108.4, 98.7, 56.3 (d, *J* = 6.0 Hz), 54.4, 49.0, 31.4 (q, *J* = 29.0 Hz), 25.8 (d, *J* = 3.1 Hz), 13.6 (d, *J* = 2.1 Hz), 10.3, and 3.9; ^19^F NMR (376 MHz, CDCl_3_): δ -66.0 (t, *J* = 10.8 Hz); HRMS (m/z): ([M]^+^ calculated for C_23_H_27_BrF_3_N_5_O_2_ = 542.1373) 542.1365 (found).

**8-(4-(Benzyloxy)-2,6-dimethoxyphenyl)-2,7-dimethyl-*N*-propyl-*N*-(4,4,4-trifluorobutyl)pyrazolo[1,5-α][1,3,5]triazin-4-amine (2j).** Compound 2j was prepared per the procedure described for synthesizing 2a. The title compound was obtained (435 mg, 78% yield) as a white solid. TLC (n-Hex:EtOAc, 2:1 v/v): *R*_f_ = 0.42; ^1^H NMR (500 MHz, CDCl_3_): δ 7.48 (d, *J* = 7.3 Hz, 2H), 7.42 (t, *J* = 7.5 Hz, 2H), 7.35 (t, *J* = 7.3 Hz, 1H), 6.32 (s, 2H), 5.10 (s, 2H), 3.99 (d, *J* = 81.5 Hz, 4H), 3.73 (s, 6H), 2.38 (s, 3H), 2.28–2.17 (m, 5H), 2.12–2.02 (m, 2H), 1.84–1.73 (m, 2H), and 0.98 (t, *J* = 7.4 Hz, 3H); ^13^C NMR (126 MHz, CDCl_3_): δ 162.0, 160.5, 159.6, 154.8, 150.0, 149.0, 137.1, 128.8, 128.4, 128.2, 127.7, 126.2, 102.1, 99.4, 92.8–91.7 (m), 70.7–70.0 (m), 56.2–55.9 (m), 52.2, 49.1, 31.4 (q, *J* = 29.1 Hz), 25.8 (d, *J* = 2.6 Hz), 21.8, 13.6 (d, *J* = 1.9 Hz), and 11.3; ^19^F NMR (376 MHz, CDCl_3_): δ -66.1 (t, *J* = 10.8 Hz).

**4-(2,7-Dimethyl-4-(propyl(4,4,4-trifluorobutyl)amino)pyrazolo[1,5-α][1,3,5]triazin-8-yl)-3,5-dimethoxyphenol (3j).** Compound 3j was prepared per the procedure described for synthesizing 3a. The title compound was obtained (388 mg, 83% yield) as a white solid. TLC (n-Hex:EtOAc, 1:1 v/v): *R*_f_ = 0.33; ^1^H NMR (500 MHz, CDCl_3_): δ 5.86 (s, 2H), 4.71–3.70 (m, 4H), 3.61 (s, 6H), 2.50 (s, 3H), 2.30–2.22 (m, 2H), 2.20 (s, 3H), 2.13 (dd, *J* = 17.5, 11.7 Hz, 2H), 1.90–1.79 (m, *J* = 15.0, 7.4 Hz, 2H), and 1.01 (t, *J* = 7.4 Hz, 3H); ^13^C NMR (126 MHz, CDCl_3_): δ 162.1, 159.7, 159.0, 155.7, 149.1, 148.4, 130.5, 128.3, 126.1, 123.9, 100.5, 97.9, 93.06 (d, *J* = 4.2 Hz), 55.4 (t, *J* = 5.0 Hz), 52.4, 49.3, 31.4 (q, *J* = 29.4 Hz), 24.6 (d, *J* = 3.4 Hz), 13.5 (d, *J* = 2.2 Hz), and 11.3; ^19^F NMR (376 MHz, CDCl_3_): δ -66.0 (t, *J* = 10.5 Hz).

**4-(2,7-Dimethyl-4-(propyl(4,4,4-trifluorobutyl)amino)pyrazolo[1,5-α][1,3,5]triazin-8-yl)-3,5-dimethoxyphenyl trifluoromethanesulfonate (4j).** Compound 4j was prepared per the procedure described for synthesizing 4a. The title compound was obtained (330 mg, 55% yield) as a white solid. TLC (n-Hex:EtOAc, 7:1 v/v): *R*_f_ = 0.17; ^1^H NMR (500 MHz, CDCl_3_): δ 6.55 (s, 2H), 4.10 (s, 2H), 3.91 (s, 2H), 3.77 (s, 6H), 2.39 (s, 3H), 2.28–2.20 (m, 2H), 2.19 (s, 3H), 2.12–2.05 (m, 2H), 1.80 (dq, 1H), and 0.99 (t, *J* = 7.4 Hz, 3H); ^13^C NMR (126 MHz, CDCl_3_): δ 162.5, 159.5, 154.4, 150.4, 150.0, 149.0, 130.5, 128.3, 126.1, 123.9, 122.7, 120.2, 117.6, 115.1, 109.6, 98.3, 98.1, 56.4, 52.3, 49.3, 31.4 (q, *J* = 29.1 Hz), 25.8, 21.9, 13.6, and 11.3; ^19^F NMR (376 MHz, CDCl_3_): δ -66.1 (t, *J* = 10.8 Hz) and -72.9.

**8-(2,6-Dimethoxy-4-(tributylstannyl)phenyl)-2,7-dimethyl-*N*-propyl-*N*-(4,4,4-trifluorobutyl)pyrazolo[1,5-α][1,3,5]triazin-4-amine (5j).** Compound 5j was prepared per the procedure described for synthesizing 5a. The title compound was obtained (185 mg, 50% yield) as a white solid. TLC (n-Hex:EtOAc, 7:1 v/v): *R*_f_ = 0.11; ^1^H NMR (500 MHz, CDCl_3_): δ 6.73 (s, 2H), 4.00 (d, *J* = 80.5 Hz, 4H), 3.78 (s, 6H), 2.38 (s, 3H), 2.29–2.20 (m, 5H), 2.13–2.04 (m, *J* = 15.2, 7.7 Hz, 2H), 1.84–1.74 (m, *J* = 14.9, 7.4 Hz, 2H), 1.67–1.52 (m, 6H), 1.43–1.33 (m, 6H), 1.17–1.02 (m, 6H), 0.98 (t, *J* = 7.4 Hz, 3H), and 0.93 (t, *J* = 7.3 Hz, 9H); ^13^C NMR (126 MHz, CDCl_3_): δ 162.0, 158.2, 154.6, 150.0, 149.0, 143.2, 130.6, 128.4, 126.2, 124.0, 112.3, 109.3, 99.7, 56.2 (dd, *J* = 16.6, 7.9 Hz), 52.2, 49.1, 31.4 (q, *J* = 28.7 Hz), 29.3, 27.6, 25.9 (d, *J* = 4.7 Hz), 21.9, 13.9, 13.8 (d, *J* = 3.7 Hz), 11.3, 9.9, and 8.6; ^19^F NMR (376 MHz, CDCl_3_): δ -66.1 (t, *J* = 10.8 Hz).

**8-(4-Bromo-2,6-dimethoxyphenyl)-2,7-dimethyl-*N*-propyl-*N*-(4,4,4-trifluorobutyl)pyrazolo[1,5-α][1,3,5]triazin-4-amine (BMK-C210).** Compound BMK-C210 was prepared per the procedure described for synthesizing BMK-C201. The title compound was obtained (70.0 mg, 66% yield) as a white solid. TLC (n-Hex:EtOAc, 7:1 v/v): *R*_f_ = 0.13; ^1^H NMR (400 MHz, CDCl_3_): δ 6.79 (s, 2H), 4.07 (s, 2H), 3.92 (s, 2H), 3.74 (s, 6H), 2.37 (s, 3H), 2.29–2.20 (m, *J* = 10.4, 7.5 Hz, 2H), 2.18 (s, 3H), 2.12–2.03 (m, *J* = 16.1, 9.3 Hz, 2H), 1.78 (dq, *J* = 14.9, 7.4 Hz, 2H), and 0.98 (t, *J* = 7.4 Hz, 3H); ^13^C NMR (126 MHz, CDCl_3_): δ 162.3, 159.3, 154.3, 150.0, 149.0, 130.5, 128.3, 126.1, 123.9, 122.5, 108.4, 98.6, 60.5, 56.5–55.7 (m, *J* = 16.8, 8.3 Hz), 52.2, 49.1, 31.4 (q, *J* = 29.2 Hz), 25.8 (d, *J* = 4.6 Hz), 13.6 (d, *J* = 3.4 Hz), and 11.3; ^19^F NMR (376 MHz, CDCl_3_): δ -66.1 (t, *J* = 10.8 Hz); HRMS (m/z): ([M]^+^ calculated for C_22_H_27_BrF_3_N_5_O_2_ = 530.1373) 530.1374 (found).

**Reference**

1. L. Lang, Y. Ma, B. M. Kim, E. M. Jagoda, K. C. Rice, L. P. Szajek, C. Contoreggi, P. W. Gold, G. P. Chrousos, W. C. Eckelman, D. O. Kiesewetter, [76Br]BMK-I-152, a non-peptide analogue for PET imaging of corticotropin-releasing hormone type 1 receptor (CRHR1). *Journal of Labelled Compounds and Radiopharmaceuticals* **52**, 394-400 (2009).
