# Supplementary material for: Structure-based drug discovery of a corticotropin-releasing hormone receptor 1 antagonist using an X-ray free-electron laser
Source: Exp Mol Med. 2023 Sep 1;55(9):2039–50. doi: 10.1038/s12276-023-01082-1 (PMC10545732; doi:10.1038/s12276-023-01082-1)
Supplement: Supplementary file 5 — validation report_8GTM [file 12276_2023_1082_MOESM5_ESM.pdf]

Full wwPDB X-ray Structure Validation Report (i)

Sep 13, 2022 – 03:16 PM JST

PDB ID	:	8GTM
Title	:	GPCR with compound 3
Deposited on	:	2022-09-08
Resolution	:	2.60 Å(reported)

This wwPDB validation report is for manuscript review

For Manuscript Review

This is a Full wwPDB X-ray Structure Validation Report.

This report is produced by the wwPDB biocuration pipeline after annotation of the structure.

We welcome your comments at *validation@mail.wwpdb.org*
A user guide is available at
https://www.wwpdb.org/validation/2017/XrayValidationReportHelp
with specific help available everywhere you see the (i) symbol.

The types of validation reports are described at http://www.wwpdb.org/validation/2017/FAQs#types.

The following versions of software and data (see references (i)) were used in the production of this report:

MolProbity	:	4.02b-467
Mogul	:	1.8.5 (274361), CSD as541be (2020)
Xtriage (Phenix)	:	1.13
EDS	:	2.30
buster-report	:	1.1.7 (2018)
Percentile statistics	:	20191225.v01 (using entries in the PDB archive December 25th 2019)
Refmac	:	5.8.0158
CCP4	:	7.0.044 (Gargrove)
Ideal geometry (proteins)	:	Engh & Huber (2001)
Ideal geometry (DNA, RNA)	:	Parkinson et al. (1996)
Validation Pipeline (wwPDB-VP)	:	2.30

1 Overall quality at a glance (i)

The following experimental techniques were used to determine the structure:
X-RAY DIFFRACTION

The reported resolution of this entry is 2.60 Å.

Percentile scores (ranging between 0-100) for global validation metrics of the entry are shown in the following graphic. The table shows the number of entries on which the scores are based.

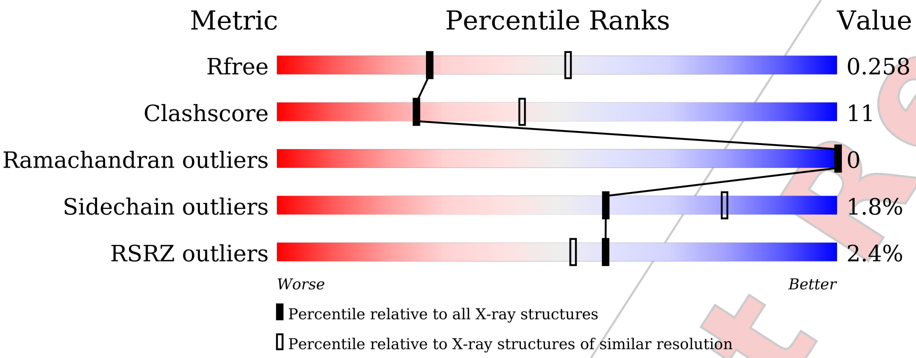

Metric	Whole archive (#Entries)	Similar resolution (#Entries, resolution range(Å))
R_{free}	130704	3163 (2.60-2.60)
Clashscore	141614	3518 (2.60-2.60)
Ramachandran outliers	138981	3455 (2.60-2.60)
Sidechain outliers	138945	3455 (2.60-2.60)
RSRZ outliers	127900	3104 (2.60-2.60)

The table below summarises the geometric issues observed across the polymeric chains and their fit to the electron density. The red, orange, yellow and green segments of the lower bar indicate the fraction of residues that contain outliers for >=3, 2, 1 and 0 types of geometric quality criteria respectively. A grey segment represents the fraction of residues that are not modelled. The numeric value for each fraction is indicated below the corresponding segment, with a dot representing fractions <=5% The upper red bar (where present) indicates the fraction of residues that have poor fit to the electron density. The numeric value is given above the bar.

Mol	Chain	Length	Quality of chain
1	AA	284	2% / 75% 17% • 7%
2	BA	160	3% / 74% 25% •

For Manuscript Review

2 Entry composition (i)

There are 3 unique types of molecules in this entry. The entry contains 3330 atoms, of which 27 are hydrogens and 0 are deuteriums.

In the tables below, the ZeroOcc column contains the number of atoms modelled with zero occupancy, the AltConf column contains the number of residues with at least one atom in alternate conformation and the Trace column contains the number of residues modelled with at most 2 atoms.

- Molecule 1 is a protein called Isoform CRF-R2 of Corticotropin-releasing factor receptor 1.

Mol	Chain	Residues	Atoms					ZeroOcc	AltConf	Trace
			Total	C	N	O	S			
1	AA	263	2046	1372	321	339	14	0	0	0

There are 25 discrepancies between the modelled and reference sequences:

Chain	Residue	Modelled	Actual	Comment	Reference
AA	103	MET	-	initiating methionine	UNP P34998-2
AA	120	ALA	VAL	engineered mutation	UNP P34998-2
AA	144	ALA	LEU	engineered mutation	UNP P34998-2
AA	156	ALA	TRP	engineered mutation	UNP P34998-2
AA	160	ALA	SER	engineered mutation	UNP P34998-2
AA	228	ALA	LYS	engineered mutation	UNP P34998-2
AA	260	ALA	PHE	engineered mutation	UNP P34998-2
AA	277	ALA	ILE	engineered mutation	UNP P34998-2
AA	309	ALA	TYR	engineered mutation	UNP P34998-2
AA	330	ALA	PHE	engineered mutation	UNP P34998-2
AA	349	ALA	SER	engineered mutation	UNP P34998-2
AA	363	ALA	TYR	engineered mutation	UNP P34998-2
AA	374	ALA	-	expression tag	UNP P34998-2
AA	375	ALA	-	expression tag	UNP P34998-2
AA	376	ALA	-	expression tag	UNP P34998-2
AA	377	HIS	-	expression tag	UNP P34998-2
AA	378	HIS	-	expression tag	UNP P34998-2
AA	379	HIS	-	expression tag	UNP P34998-2
AA	380	HIS	-	expression tag	UNP P34998-2
AA	381	HIS	-	expression tag	UNP P34998-2
AA	382	HIS	-	expression tag	UNP P34998-2
AA	383	HIS	-	expression tag	UNP P34998-2
AA	384	HIS	-	expression tag	UNP P34998-2
AA	385	HIS	-	expression tag	UNP P34998-2
AA	386	HIS	-	expression tag	UNP P34998-2

- Molecule 2 is a protein called Endolysin.

For Manuscript Review

Mol	Chain	Residues	Atoms					ZeroOcc	AltConf	Trace
			Total	C	N	O	S			
2	BA	160	1220	770	216	230	4	0	0	0

There are 2 discrepancies between the modelled and reference sequences:

Chain	Residue	Modelled	Actual	Comment	Reference
BA	1052	SER	CYS	engineered mutation	UNP A0A346FJK3
BA	1095	SER	CYS	engineered mutation	UNP A0A346FJK3

- Molecule 3 is 7-(4-bromanyl-2,6-dimethoxy-phenyl)-4,8-dimethyl- {N}, {N}-bis[4,4,4-tris(fluoranyl)butyl]-1$l^{4},3,5,9-tetrazabicyclo[4.3.0]nona-1(6),2,4,8-tetraen-2-amine (three-letter code: 0VI) (formula: $C_{23}H_{27}BrF_6N_5O_2$) (labeled as "Ligand of Interest" by depositor).

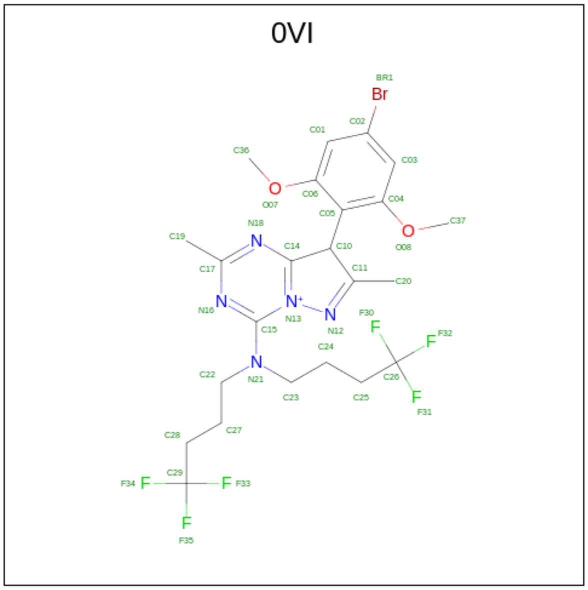

Mol	Chain	Residues	Atoms							ZeroOcc	AltConf
			Total	Br	C	F	H	N	O		
3	AA	1	64	1	23	6	27	5	2	0	0

For Manuscript Review

3 Residue-property plots (i)

These plots are drawn for all protein, RNA, DNA and oligosaccharide chains in the entry. The first graphic for a chain summarises the proportions of the various outlier classes displayed in the second graphic. The second graphic shows the sequence view annotated by issues in geometry and electron density. Residues are color-coded according to the number of geometric quality criteria for which they contain at least one outlier: green = 0, yellow = 1, orange = 2 and red = 3 or more. A red dot above a residue indicates a poor fit to the electron density (RSRZ > 2). Stretches of 2 or more consecutive residues without any outlier are shown as a green connector. Residues present in the sample, but not in the model, are shown in grey.

- Molecule 1: Isoform CRF-R2 of Corticotropin-releasing factor receptor 1

Chain AA:

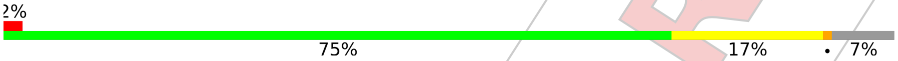

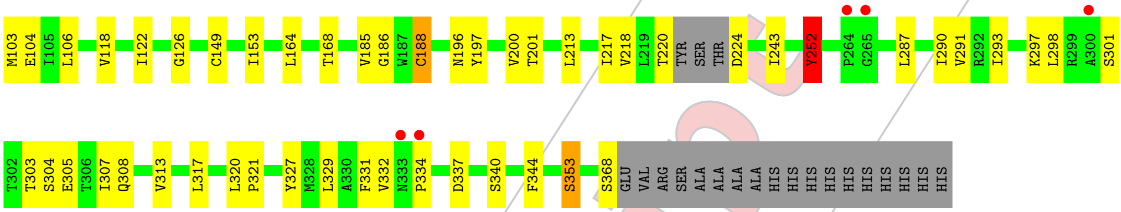

- Molecule 2: Endolysin

Chain BA:

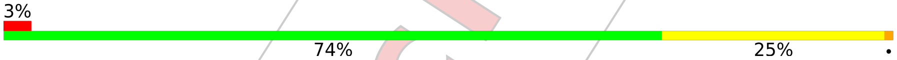

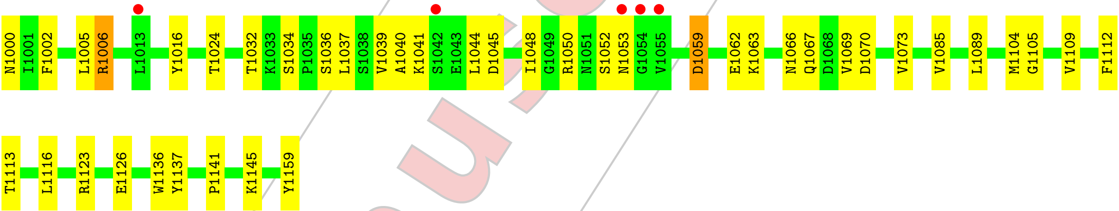

For Manuscript Review

4 Data and refinement statistics (i)

Property	Value	Source
Space group	C 1 2 1	Depositor
Cell constants<br>a, b, c, α, β, γ	95.66Å 70.65Å 86.75Å<br>90.00° 97.82° 90.00°	Depositor
Resolution (Å)	56.64 – 2.60<br>56.64 – 2.60	Depositor<br>EDS
% Data completeness<br>(in resolution range)	100.0 (56.64-2.60)<br>99.9 (56.64-2.60)	Depositor<br>EDS
R_{merge}	(Not available)	Depositor
R_{sym}	(Not available)	Depositor
$< I/\sigma(I) >$ [1]	1.67 (at 2.61Å)	Xtriage
Refinement program	PHENIX 1.19.2_4158	Depositor
R, R_{free}	0.219 , 0.260<br>0.214 , 0.258	Depositor<br>DCC
R_{free} test set	521 reflections (2.93%)	wwPDB-VP
Wilson B-factor (Å^2)	63.3	Xtriage
Anisotropy	0.343	Xtriage
Bulk solvent k_{sol}(e/Å^3), B_{sol}(Å^2)	0.30 , 78.3	EDS
L-test for twinning[2]	$< \|L\| >$ = 0.46, $< L^2 >$ = 0.28	Xtriage
Estimated twinning fraction	No twinning to report.	Xtriage
F_o,F_c correlation	0.94	EDS
Total number of atoms	3330	wwPDB-VP
Average B, all atoms (Å^2)	66.0	wwPDB-VP

Xtriage's analysis on translational NCS is as follows: *The largest off-origin peak in the Patterson function is 4.80% of the height of the origin peak. No significant pseudotranslation is detected.*

[1] Intensities estimated from amplitudes.
[2] Theoretical values of $< |L| >$, $< L^2 >$ for acentric reflections are 0.5, 0.333 respectively for untwinned datasets, and 0.375, 0.2 for perfectly twinned datasets.

For Manuscript Review

5 Model quality (i)

5.1 Standard geometry (i)

Bond lengths and bond angles in the following residue types are not validated in this section: 0VI

The Z score for a bond length (or angle) is the number of standard deviations the observed value is removed from the expected value. A bond length (or angle) with $|Z| > 5$ is considered an outlier worth inspection. RMSZ is the root-mean-square of all Z scores of the bond lengths (or angles).

Mol	Chain	Bond lengths		Bond angles	
		RMSZ	#|Z| >5	RMSZ	#|Z| >5
1	AA	0.63	4/2106 (0.2%)	0.57	1/2882 (0.0%)
2	BA	0.68	5/1240 (0.4%)	0.77	2/1683 (0.1%)
All	All	0.65	9/3346 (0.3%)	0.65	3/4565 (0.1%)

Chiral center outliers are detected by calculating the chiral volume of a chiral center and verifying if the center is modelled as a planar moiety or with the opposite hand.A planarity outlier is detected by checking planarity of atoms in a peptide group, atoms in a mainchain group or atoms of a sidechain that are expected to be planar.

Mol	Chain	#Chirality outliers	#Planarity outliers
1	AA	0	1

All (9) bond length outliers are listed below:

Mol	Chain	Res	Type	Atoms	Z	Observed(Å)	Ideal(Å)
1	AA	252	TYR	CD1-CE1	-9.78	1.24	1.39
1	AA	252	TYR	CE2-CZ	-9.62	1.26	1.38
1	AA	252	TYR	CE1-CZ	9.40	1.50	1.38
1	AA	252	TYR	CZ-OH	8.15	1.51	1.37
2	BA	1006	ARG	CZ-NH1	7.50	1.42	1.33
2	BA	1006	ARG	CB-CG	-7.42	1.32	1.52
2	BA	1006	ARG	CD-NE	-7.38	1.33	1.46
2	BA	1006	ARG	CG-CD	-6.99	1.34	1.51
2	BA	1006	ARG	NE-CZ	-6.09	1.25	1.33

All (3) bond angle outliers are listed below:

Mol	Chain	Res	Type	Atoms	Z	Observed(°)	Ideal(°)
2	BA	1006	ARG	NE-CZ-NH2	-15.98	112.31	120.30
2	BA	1006	ARG	NE-CZ-NH1	5.83	123.21	120.30

Continued on next page...

For Manuscript Review

Continued from previous page...

Mol	Chain	Res	Type	Atoms	Z	Observed(°)	Ideal(°)
1	AA	252	TYR	CZ-CE2-CD2	-5.60	114.76	119.80

There are no chirality outliers.

All (1) planarity outliers are listed below:

Mol	Chain	Res	Type	Group
1	AA	252	TYR	Sidechain

5.2 Too-close contacts (i)

In the following table, the Non-H and H(model) columns list the number of non-hydrogen atoms and hydrogen atoms in the chain respectively. The H(added) column lists the number of hydrogen atoms added and optimized by MolProbity. The Clashes column lists the number of clashes within the asymmetric unit, whereas Symm-Clashes lists symmetry-related clashes.

Mol	Chain	Non-H	H(model)	H(added)	Clashes	Symm-Clashes
1	AA	2046	0	1999	38	1
2	BA	1220	0	1184	38	1
3	AA	37	27	0	0	0
All	All	3303	27	3183	70	1

The all-atom clashscore is defined as the number of clashes found per 1000 atoms (including hydrogen atoms). The all-atom clashscore for this structure is 11.

All (70) close contacts within the same asymmetric unit are listed below, sorted by their clash magnitude.

Atom-1	Atom-2	Interatomic distance (Å)	Clash overlap (Å)
2:BA:1032:THR:HG21	2:BA:1039:VAL:HG23	1.38	1.05
1:AA:103:MET:HG2	1:AA:104:GLU:H	1.31	0.95
2:BA:1123:ARG:NH2	2:BA:1126:GLU:OE1	2.09	0.85
1:AA:213:LEU:HD23	1:AA:290:ILE:HD13	1.69	0.74
2:BA:1037:LEU:HD22	2:BA:1037:LEU:O	1.89	0.73
2:BA:1034:SER:HB3	2:BA:1039:VAL:HG21	1.77	0.66
1:AA:337:ASP:HB2	1:AA:340:SER:HB3	1.79	0.65
2:BA:1041:LYS:O	2:BA:1044:LEU:HB3	1.98	0.64
1:AA:103:MET:HG2	1:AA:104:GLU:N	2.11	0.62
1:AA:334:PRO:HG3	1:AA:344:PHE:CD2	2.36	0.60
1:AA:220:THR:C	2:BA:1000:ASN:N	2.55	0.60
1:AA:224:ASP:N	2:BA:1159:TYR:O	2.34	0.59

Continued on next page...

For Manuscript Review

Continued from previous page...

Atom-1	Atom-2	Interatomic distance (Å)	Clash overlap (Å)
2:BA:1048:ILE:HG23	2:BA:1050:ARG:H	1.66	0.59
1:AA:329:LEU:HA	1:AA:332:VAL:HG23	1.84	0.58
1:AA:298:LEU:HA	1:AA:301:SER:HB2	1.85	0.58
2:BA:1037:LEU:HD22	2:BA:1040:ALA:HB3	1.88	0.56
1:AA:164:LEU:O	1:AA:168:THR:HG23	2.06	0.56
1:AA:220:THR:C	2:BA:1000:ASN:HA	2.25	0.56
1:AA:224:ASP:N	2:BA:1159:TYR:C	2.59	0.56
2:BA:1112:PHE:O	2:BA:1116:LEU:HG	2.05	0.56
1:AA:149:CYS:O	1:AA:153:ILE:HG13	2.06	0.56
2:BA:1045:ASP:CB	2:BA:1052:SER:HB2	2.35	0.56
2:BA:1066:ASN:O	2:BA:1070:ASP:OD2	2.25	0.55
1:AA:220:THR:C	2:BA:1000:ASN:CA	2.75	0.54
2:BA:1141:PRO:O	2:BA:1145:LYS:HG3	2.08	0.54
1:AA:126:GLY:HA3	1:AA:353:SER:OG	2.08	0.54
2:BA:1037:LEU:C	2:BA:1037:LEU:HD13	2.30	0.52
1:AA:303:THR:HG22	1:AA:305:GLU:H	1.74	0.52
1:AA:327:TYR:O	1:AA:331:PHE:CE2	2.63	0.52
2:BA:1036:SER:O	2:BA:1039:VAL:HG22	2.10	0.52
1:AA:334:PRO:HG3	1:AA:344:PHE:CE2	2.46	0.51
2:BA:1002:PHE:CD1	2:BA:1062:GLU:HG3	2.47	0.50
2:BA:1137:TYR:OH	2:BA:1145:LYS:HE2	2.11	0.50
2:BA:1048:ILE:HG23	2:BA:1050:ARG:N	2.27	0.48
1:AA:304:SER:O	1:AA:307:ILE:HG13	2.14	0.48
1:AA:217:ILE:HG13	1:AA:218:VAL:HG23	1.96	0.47
2:BA:1034:SER:CB	2:BA:1039:VAL:HG21	2.44	0.47
1:AA:243:ILE:HD13	1:AA:243:ILE:HA	1.79	0.47
1:AA:197:TYR:O	1:AA:201:THR:HG23	2.16	0.46
1:AA:307:ILE:HG13	1:AA:308:GLN:N	2.32	0.45
2:BA:1105:GLY:O	2:BA:1109:VAL:HG23	2.16	0.45
1:AA:196:ASN:O	1:AA:200:VAL:HG23	2.16	0.45
2:BA:1016:TYR:CE1	2:BA:1024:THR:OG1	2.69	0.45
2:BA:1059:ASP:N	2:BA:1059:ASP:OD1	2.50	0.45
1:AA:185:VAL:HG13	1:AA:188:CYS:HB2	1.99	0.45
1:AA:220:THR:O	2:BA:1000:ASN:HA	2.17	0.45
1:AA:307:ILE:HG13	1:AA:308:GLN:H	1.82	0.44
1:AA:287:LEU:O	1:AA:291:VAL:HG23	2.18	0.44
2:BA:1104:MET:HE1	2:BA:1136:TRP:CD1	2.53	0.43
2:BA:1044:LEU:HD23	2:BA:1044:LEU:O	2.17	0.43
2:BA:1063:LYS:O	2:BA:1067:GLN:HG3	2.18	0.43
1:AA:320:LEU:HB3	1:AA:321:PRO:HD3	2.00	0.43
2:BA:1044:LEU:HD23	2:BA:1044:LEU:C	2.39	0.43

Continued on next page...

For Manuscript Review

Continued from previous page...

Atom-1	Atom-2	Interatomic distance (Å)	Clash overlap (Å)
1:AA:298:LEU:N	1:AA:298:LEU:HD23	2.34	0.43
2:BA:1085:VAL:O	2:BA:1089:LEU:HG	2.18	0.42
2:BA:1069:VAL:O	2:BA:1073:VAL:HG23	2.19	0.42
1:AA:252:TYR:CD1	1:AA:252:TYR:O	2.72	0.42
1:AA:185:VAL:HG22	1:AA:186:GLY:N	2.35	0.42
2:BA:1036:SER:OG	2:BA:1039:VAL:HG13	2.19	0.42
2:BA:1116:LEU:HD23	2:BA:1116:LEU:HA	1.87	0.41
2:BA:1034:SER:HB3	2:BA:1039:VAL:CG2	2.47	0.41
1:AA:329:LEU:HA	1:AA:332:VAL:CG2	2.50	0.41
2:BA:1137:TYR:OH	2:BA:1145:LYS:CE	2.68	0.41
2:BA:1005:LEU:HD13	2:BA:1005:LEU:HA	1.77	0.41
1:AA:293:ILE:O	1:AA:297:LYS:O	2.39	0.41
1:AA:313:VAL:O	1:AA:317:LEU:HG	2.21	0.41
2:BA:1053:ASN:OD1	2:BA:1053:ASN:N	2.54	0.41
1:AA:164:LEU:HD23	1:AA:164:LEU:HA	1.92	0.40
1:AA:118:VAL:O	1:AA:122:ILE:HG12	2.22	0.40
1:AA:303:THR:CG2	1:AA:305:GLU:OE1	2.69	0.40

All (1) symmetry-related close contacts are listed below. The label for Atom-2 includes the symmetry operator and encoded unit-cell translations to be applied.

Atom-1	Atom-2	Interatomic distance (Å)	Clash overlap (Å)
1:AA:252:TYR:OH	2:BA:1006:ARG:NH2[2_555]	2.15	0.05

5.3 Torsion angles (i)

5.3.1 Protein backbone (i)

In the following table, the Percentiles column shows the percent Ramachandran outliers of the chain as a percentile score with respect to all X-ray entries followed by that with respect to entries of similar resolution.

The Analysed column shows the number of residues for which the backbone conformation was analysed, and the total number of residues.

Mol	Chain	Analysed	Favoured	Allowed	Outliers	Percentiles	
1	AA	259/284 (91%)	253 (98%)	6 (2%)	0	100	100
2	BA	158/160 (99%)	155 (98%)	3 (2%)	0	100	100
All	All	417/444 (94%)	408 (98%)	9 (2%)	0	100	100

For Manuscript Review

There are no Ramachandran outliers to report.

5.3.2 Protein sidechains (i)

In the following table, the Percentiles column shows the percent sidechain outliers of the chain as a percentile score with respect to all X-ray entries followed by that with respect to entries of similar resolution.

The Analysed column shows the number of residues for which the sidechain conformation was analysed, and the total number of residues.

Mol	Chain	Analysed	Rotameric	Outliers	Percentiles	
1	AA	208/243 (86%)	204 (98%)	4 (2%)	57	79
2	BA	122/134 (91%)	120 (98%)	2 (2%)	62	82
All	All	330/377 (88%)	324 (98%)	6 (2%)	59	80

All (6) residues with a non-rotameric sidechain are listed below:

Mol	Chain	Res	Type
1	AA	106	LEU
1	AA	188	CYS
1	AA	353	SER
1	AA	368	SER
2	BA	1059	ASP
2	BA	1113	THR

Sometimes sidechains can be flipped to improve hydrogen bonding and reduce clashes. There are no such sidechains identified.

5.3.3 RNA (i)

There are no RNA molecules in this entry.

5.4 Non-standard residues in protein, DNA, RNA chains (i)

There are no non-standard protein/DNA/RNA residues in this entry.

5.5 Carbohydrates (i)

There are no monosaccharides in this entry.

For Manuscript Review

5.6 Ligand geometry (i)

1 ligand is modelled in this entry.

In the following table, the Counts columns list the number of bonds (or angles) for which Mogul statistics could be retrieved, the number of bonds (or angles) that are observed in the model and the number of bonds (or angles) that are defined in the Chemical Component Dictionary. The Link column lists molecule types, if any, to which the group is linked. The Z score for a bond length (or angle) is the number of standard deviations the observed value is removed from the expected value. A bond length (or angle) with $|Z| > 2$ is considered an outlier worth inspection. RMSZ is the root-mean-square of all Z scores of the bond lengths (or angles).

Mol	Type	Chain	Res	Link	Bond lengths			Bond angles		
					Counts	RMSZ	$\#\|Z\| > 2$	Counts	RMSZ	$\#\|Z\| > 2$
3	0VI	AA	401	-	36,39,39	3.15	7 (19%)	47,58,58	2.62	14 (29%)

In the following table, the Chirals column lists the number of chiral outliers, the number of chiral centers analysed, the number of these observed in the model and the number defined in the Chemical Component Dictionary. Similar counts are reported in the Torsion and Rings columns. '-' means no outliers of that kind were identified.

Mol	Type	Chain	Res	Link	Chirals	Torsions	Rings
3	0VI	AA	401	-	-	7/26/38/38	0/3/3/3

All (7) bond length outliers are listed below:

Mol	Chain	Res	Type	Atoms	Z	Observed(Å)	Ideal(Å)
3	AA	401	0VI	C06-C05	-8.02	1.27	1.39
3	AA	401	0VI	C14-N13	8.01	1.44	1.36
3	AA	401	0VI	N13-N12	7.93	1.50	1.38
3	AA	401	0VI	C11-N12	7.83	1.45	1.29
3	AA	401	0VI	C04-C05	7.70	1.52	1.39
3	AA	401	0VI	O08-C04	4.09	1.43	1.37
3	AA	401	0VI	C17-N16	2.62	1.38	1.34

All (14) bond angle outliers are listed below:

Mol	Chain	Res	Type	Atoms	Z	Observed(^{o})	Ideal(^{o})
3	AA	401	0VI	C17-N18-C14	8.90	120.36	114.69
3	AA	401	0VI	C15-N16-C17	7.15	119.79	114.75
3	AA	401	0VI	O08-C04-C05	6.36	122.23	115.54
3	AA	401	0VI	O07-C06-C05	-4.51	110.79	115.54
3	AA	401	0VI	O08-C04-C03	-4.24	116.82	124.12
3	AA	401	0VI	C04-C05-C10	-3.80	110.62	122.14

Continued on next page...

For Manuscript Review

Continued from previous page...

Mol	Chain	Res	Type	Atoms	Z	Observed(°)	Ideal(°)
3	AA	401	0VI	C06-C01-C02	3.18	122.37	118.68
3	AA	401	0VI	C06-C05-C10	2.84	130.76	122.14
3	AA	401	0VI	N18-C17-N16	-2.75	120.67	125.72
3	AA	401	0VI	C28-C27-C22	-2.72	107.42	112.78
3	AA	401	0VI	C22-N21-C15	-2.42	116.23	121.25
3	AA	401	0VI	BR1-C02-C01	2.36	122.55	119.27
3	AA	401	0VI	O07-C06-C01	2.35	128.16	124.12
3	AA	401	0VI	C27-C28-C29	-2.23	108.89	113.84

There are no chirality outliers.

All (7) torsion outliers are listed below:

Mol	Chain	Res	Type	Atoms
3	AA	401	0VI	N16-C15-N21-C22
3	AA	401	0VI	N21-C23-C24-C25
3	AA	401	0VI	C24-C23-N21-C22
3	AA	401	0VI	N21-C22-C27-C28
3	AA	401	0VI	C24-C23-N21-C15
3	AA	401	0VI	C24-C25-C26-F31
3	AA	401	0VI	C24-C25-C26-F32

There are no ring outliers.

No monomer is involved in short contacts.

The following is a two-dimensional graphical depiction of Mogul quality analysis of bond lengths, bond angles, torsion angles, and ring geometry for all instances of the Ligand of Interest. In addition, ligands with molecular weight > 250 and outliers as shown on the validation Tables will also be included. For torsion angles, if less then 5% of the Mogul distribution of torsion angles is within 10 degrees of the torsion angle in question, then that torsion angle is considered an outlier. Any bond that is central to one or more torsion angles identified as an outlier by Mogul will be highlighted in the graph. For rings, the root-mean-square deviation (RMSD) between the ring in question and similar rings identified by Mogul is calculated over all ring torsion angles. If the average RMSD is greater than 60 degrees and the minimal RMSD between the ring in question and any Mogul-identified rings is also greater than 60 degrees, then that ring is considered an outlier. The outliers are highlighted in purple. The color gray indicates Mogul did not find sufficient equivalents in the CSD to analyse the geometry.

For Manuscript Review

Ligand 0VI AA 401

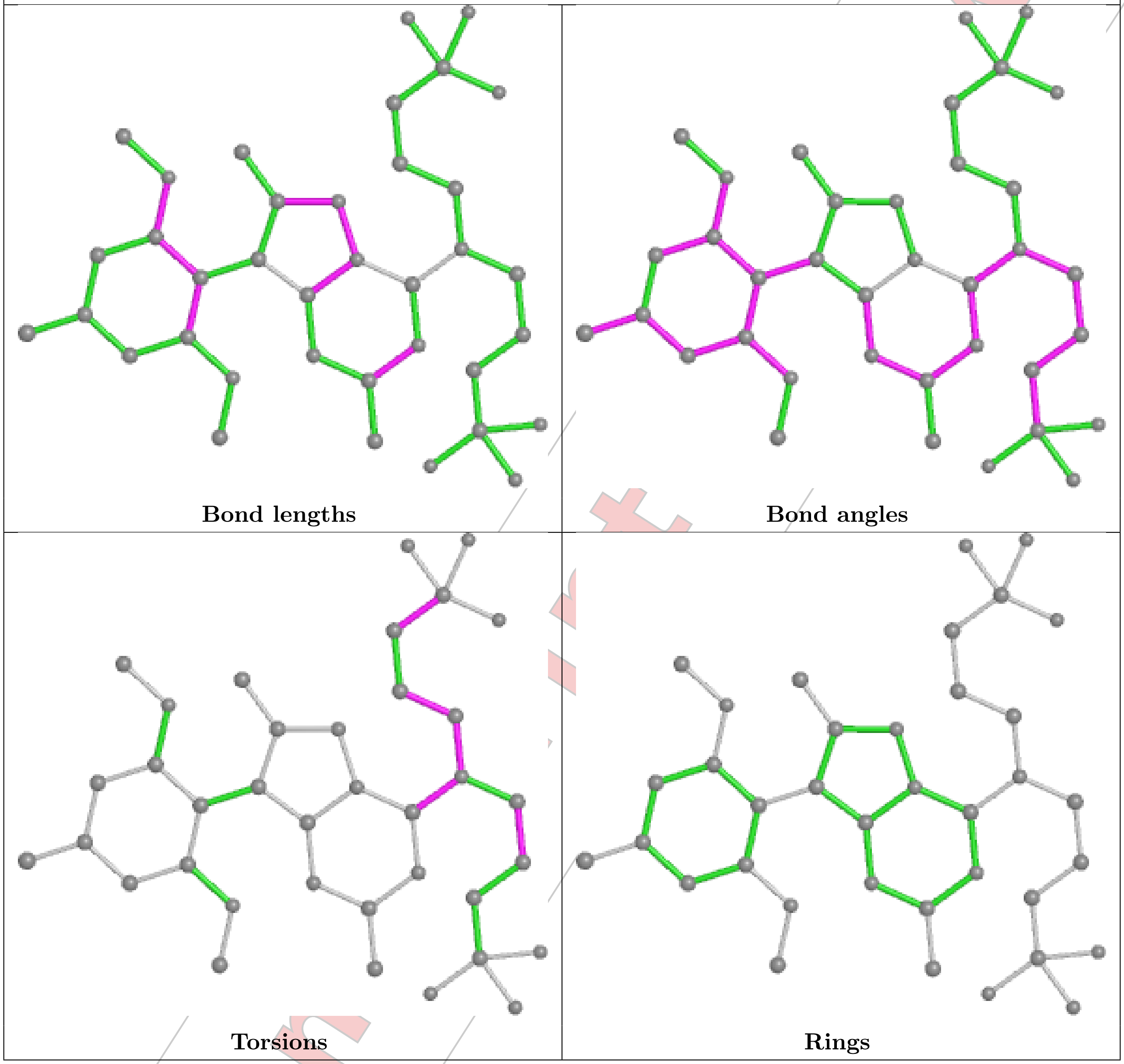

5.7 Other polymers ⓘ

There are no such residues in this entry.

5.8 Polymer linkage issues ⓘ

There are no chain breaks in this entry.

For Manuscript Review

6 Fit of model and data (i)

6.1 Protein, DNA and RNA chains (i)

In the following table, the column labelled '#RSRZ> 2' contains the number (and percentage) of RSRZ outliers, followed by percent RSRZ outliers for the chain as percentile scores relative to all X-ray entries and entries of similar resolution. The OWAB column contains the minimum, median, 95th percentile and maximum values of the occupancy-weighted average B-factor per residue. The column labelled 'Q< 0.9' lists the number of (and percentage) of residues with an average occupancy less than 0.9.

Mol	Chain	Analysed	<RSRZ>	#RSRZ>2			OWAB(Å^2)	Q<0.9
1	AA	263/284 (92%)	-0.21	5 (1%)	66	62	40, 63, 106, 127	0
2	BA	160/160 (100%)	-0.28	5 (3%)	49	42	42, 61, 109, 125	0
All	All	423/444 (95%)	-0.24	10 (2%)	59	53	40, 62, 108, 127	0

All (10) RSRZ outliers are listed below:

Mol	Chain	Res	Type	RSRZ
1	AA	264	PRO	5.3
1	AA	265	GLY	3.9
2	BA	1055	VAL	3.8
2	BA	1054	GLY	3.6
2	BA	1053	ASN	3.1
1	AA	333	ASN	2.2
1	AA	300	ALA	2.1
2	BA	1013	LEU	2.1
1	AA	334	PRO	2.1
2	BA	1042	SER	2.0

6.2 Non-standard residues in protein, DNA, RNA chains (i)

There are no non-standard protein/DNA/RNA residues in this entry.

6.3 Carbohydrates (i)

There are no monosaccharides in this entry.

For Manuscript Review

6.4 Ligands (i)

In the following table, the Atoms column lists the number of modelled atoms in the group and the number defined in the chemical component dictionary. The B-factors column lists the minimum, median, 95th percentile and maximum values of B factors of atoms in the group. The column labelled 'Q< 0.9' lists the number of atoms with occupancy less than 0.9.

Mol	Type	Chain	Res	Atoms	RSCC	RSR	B-factors(Å^2)	Q<0.9
3	0VI	AA	401	37/37	0.98	0.17	45,60,74,77	0

The following is a graphical depiction of the model fit to experimental electron density of all instances of the Ligand of Interest. In addition, ligands with molecular weight > 250 and outliers as shown on the geometry validation Tables will also be included. Each fit is shown from different orientation to approximate a three-dimensional view.

Electron density around 0VI AA 401:

2mF$_o$-DF$_c$ (at 0.7 rmsd) in gray
mF$_o$-DF$_c$ (at 3 rmsd) in purple (negative)
and green (positive)

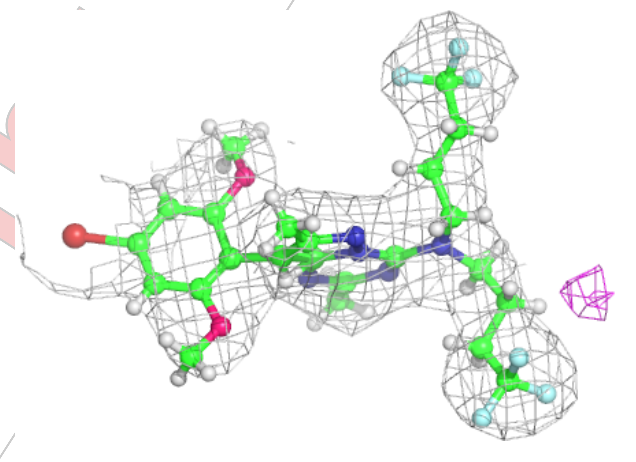

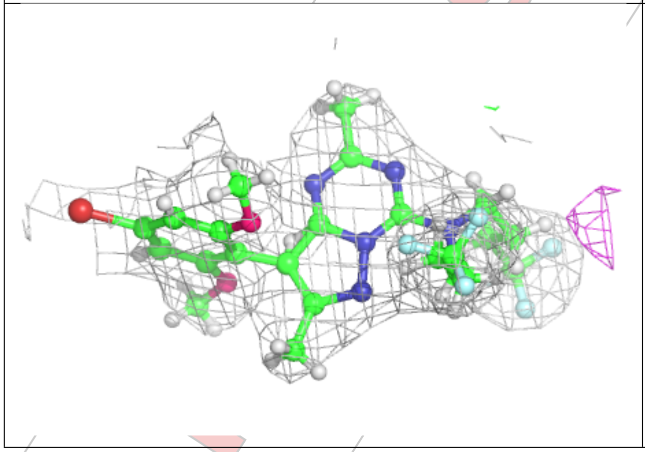

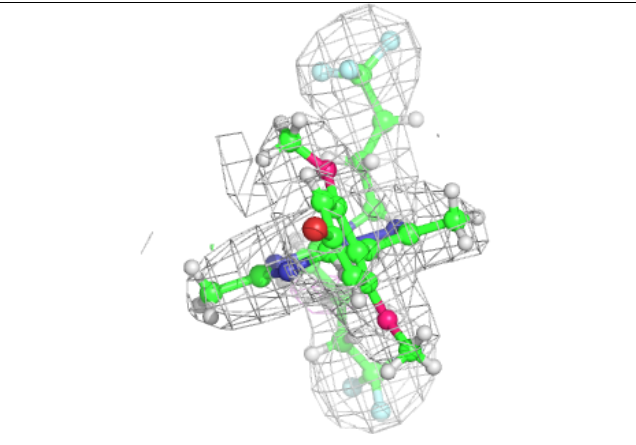

6.5 Other polymers (i)

There are no such residues in this entry.

For Manuscript Review
