# Supplementary material for: Structure-based drug discovery of a corticotropin-releasing hormone receptor 1 antagonist using an X-ray free-electron laser
Source: Exp Mol Med. 2023 Sep 1;55(9):2039–50. doi: 10.1038/s12276-023-01082-1 (PMC10545732; doi:10.1038/s12276-023-01082-1)
Supplement: Supplementary file 6 — validation report_8GTI [file 12276_2023_1082_MOESM6_ESM.pdf]

Full wwPDB X-ray Structure Validation Report (i)

Sep 13, 2022 – 03:13 PM JST

PDB ID	:	8GTI
Title	:	GPCR with compound 2
Deposited on	:	2022-09-08
Resolution	:	2.20 Å(reported)

This wwPDB validation report is for manuscript review

For Manuscript Review

This is a Full wwPDB X-ray Structure Validation Report.

This report is produced by the wwPDB biocuration pipeline after annotation of the structure.

We welcome your comments at *validation@mail.wwpdb.org*
A user guide is available at
https://www.wwpdb.org/validation/2017/XrayValidationReportHelp
with specific help available everywhere you see the (i) symbol.

The types of validation reports are described at http://www.wwpdb.org/validation/2017/FAQs#types.

The following versions of software and data (see references (i)) were used in the production of this report:

MolProbity	:	4.02b-467
Mogul	:	1.8.5 (274361), CSD as541be (2020)
Xtriage (Phenix)	:	1.13
EDS	:	2.30
buster-report	:	1.1.7 (2018)
Percentile statistics	:	20191225.v01 (using entries in the PDB archive December 25th 2019)
Refmac	:	5.8.0158
CCP4	:	7.0.044 (Gargrove)
Ideal geometry (proteins)	:	Engh & Huber (2001)
Ideal geometry (DNA, RNA)	:	Parkinson et al. (1996)
Validation Pipeline (wwPDB-VP)	:	2.30

1 Overall quality at a glance (i)

The following experimental techniques were used to determine the structure:
X-RAY DIFFRACTION

The reported resolution of this entry is 2.20 Å.

Percentile scores (ranging between 0-100) for global validation metrics of the entry are shown in the following graphic. The table shows the number of entries on which the scores are based.

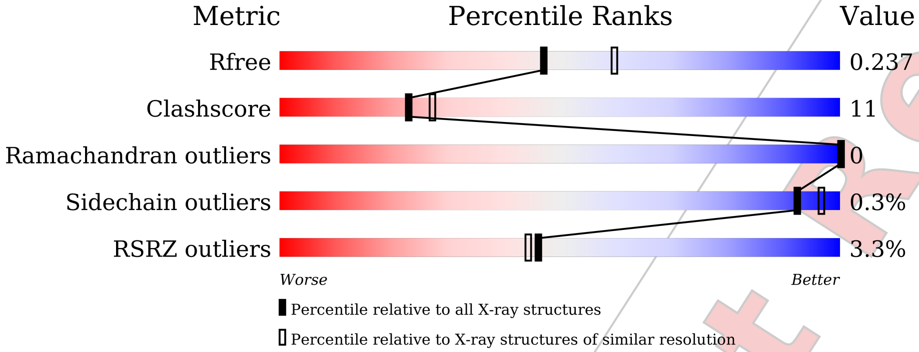

Metric	Whole archive (#Entries)	Similar resolution (#Entries, resolution range(Å))
R_{free}	130704	4898 (2.20-2.20)
Clashscore	141614	5594 (2.20-2.20)
Ramachandran outliers	138981	5503 (2.20-2.20)
Sidechain outliers	138945	5504 (2.20-2.20)
RSRZ outliers	127900	4800 (2.20-2.20)

The table below summarises the geometric issues observed across the polymeric chains and their fit to the electron density. The red, orange, yellow and green segments of the lower bar indicate the fraction of residues that contain outliers for >=3, 2, 1 and 0 types of geometric quality criteria respectively. A grey segment represents the fraction of residues that are not modelled. The numeric value for each fraction is indicated below the corresponding segment, with a dot representing fractions <=5% The upper red bar (where present) indicates the fraction of residues that have poor fit to the electron density. The numeric value is given above the bar.

Mol	Chain	Length	Quality of chain
1	A	284	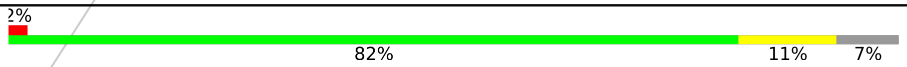
2	B	160	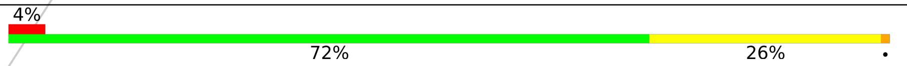

For Manuscript Review

2 Entry composition (i)

There are 4 unique types of molecules in this entry. The entry contains 3568 atoms, of which 165 are hydrogens and 0 are deuteriums.

In the tables below, the ZeroOcc column contains the number of atoms modelled with zero occupancy, the AltConf column contains the number of residues with at least one atom in alternate conformation and the Trace column contains the number of residues modelled with at most 2 atoms.

- Molecule 1 is a protein called Isoform CRF-R2 of Corticotropin-releasing factor receptor 1.

Mol	Chain	Residues	Atoms					ZeroOcc	AltConf	Trace
			Total	C	N	O	S			
1	A	263	2056	1377	324	341	14	0	0	0

There are 25 discrepancies between the modelled and reference sequences:

Chain	Residue	Modelled	Actual	Comment	Reference
A	103	MET	-	initiating methionine	UNP P34998-2
A	120	ALA	VAL	engineered mutation	UNP P34998-2
A	144	ALA	LEU	engineered mutation	UNP P34998-2
A	156	ALA	TRP	engineered mutation	UNP P34998-2
A	160	ALA	SER	engineered mutation	UNP P34998-2
A	228	ALA	LYS	engineered mutation	UNP P34998-2
A	260	ALA	PHE	engineered mutation	UNP P34998-2
A	277	ALA	ILE	engineered mutation	UNP P34998-2
A	309	ALA	TYR	engineered mutation	UNP P34998-2
A	330	ALA	PHE	engineered mutation	UNP P34998-2
A	349	ALA	SER	engineered mutation	UNP P34998-2
A	363	ALA	TYR	engineered mutation	UNP P34998-2
A	374	ALA	-	expression tag	UNP P34998-2
A	375	ALA	-	expression tag	UNP P34998-2
A	376	ALA	-	expression tag	UNP P34998-2
A	377	HIS	-	expression tag	UNP P34998-2
A	378	HIS	-	expression tag	UNP P34998-2
A	379	HIS	-	expression tag	UNP P34998-2
A	380	HIS	-	expression tag	UNP P34998-2
A	381	HIS	-	expression tag	UNP P34998-2
A	382	HIS	-	expression tag	UNP P34998-2
A	383	HIS	-	expression tag	UNP P34998-2
A	384	HIS	-	expression tag	UNP P34998-2
A	385	HIS	-	expression tag	UNP P34998-2
A	386	HIS	-	expression tag	UNP P34998-2

- Molecule 2 is a protein called Endolysin.

For Manuscript Review

Mol	Chain	Residues	Atoms					ZeroOcc	AltConf	Trace
			Total	C	N	O	S			
2	B	160	1231	776	217	234	4	0	0	0

There are 2 discrepancies between the modelled and reference sequences:

Chain	Residue	Modelled	Actual	Comment	Reference
B	1052	SER	CYS	engineered mutation	UNP A0A346FJK3
B	1095	SER	CYS	engineered mutation	UNP A0A346FJK3

- Molecule 3 is OLEIC ACID (three-letter code: OLA) (formula: $C_{18}H_{34}O_2$) (labeled as "Ligand of Interest" by depositor).

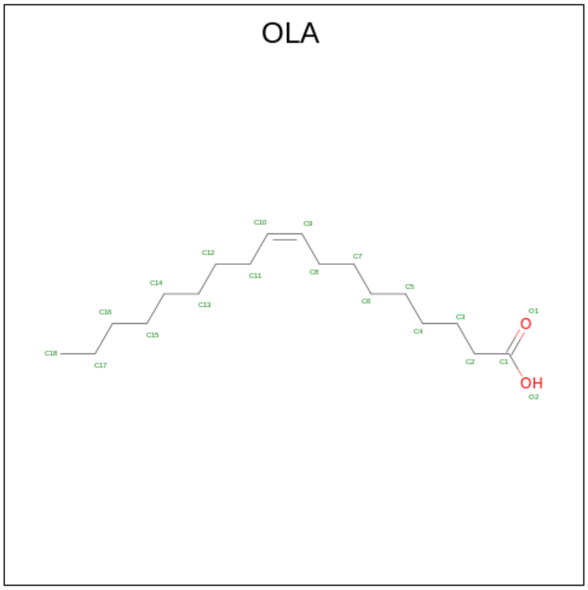

Mol	Chain	Residues	Atoms				ZeroOcc	AltConf
			Total	C	H	O		
3	A	1	24	8	14	2	0	1
3	A	1	53	18	33	2	0	0
3	A	1	53	18	33	2	0	0
3	A	1	37	13	22	2	0	0
3	A	1	53	18	33	2	0	0

- Molecule 4 is 8-(4-bromanyl-2,6-dimethoxy-phenyl)- {N}-butyl- {N}-(cyclopropylmethyl)-2,7-dimethyl-pyrazolo[1,5-a][1,3,5]triazin-4-amine (three-letter code: 0JS) (formula: $C_{23}H_{30}BrN_5O_2$) (labeled as "Ligand of Interest" by depositor).

For Manuscript Review

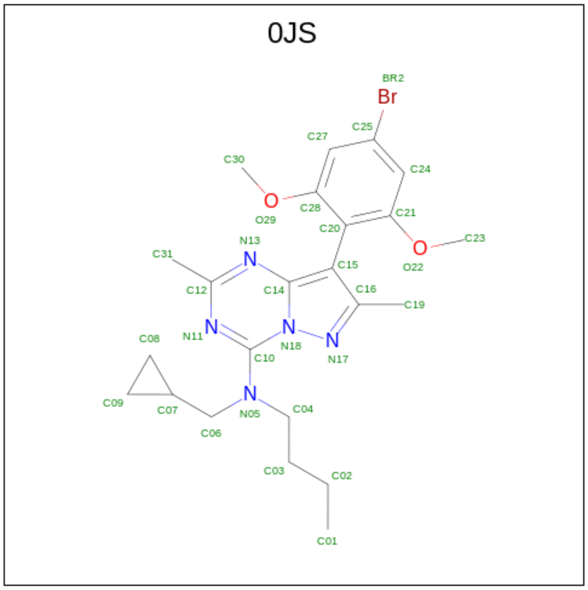

Mol	Chain	Residues	Atoms	ZeroOcc	AltConf
4	A	1	Total Br C H N O<br>61 1 23 30 5 2	0	0

For Manuscript Review

3 Residue-property plots (i)

These plots are drawn for all protein, RNA, DNA and oligosaccharide chains in the entry. The first graphic for a chain summarises the proportions of the various outlier classes displayed in the second graphic. The second graphic shows the sequence view annotated by issues in geometry and electron density. Residues are color-coded according to the number of geometric quality criteria for which they contain at least one outlier: green = 0, yellow = 1, orange = 2 and red = 3 or more. A red dot above a residue indicates a poor fit to the electron density (RSRZ > 2). Stretches of 2 or more consecutive residues without any outlier are shown as a green connector. Residues present in the sample, but not in the model, are shown in grey.

- Molecule 1: Isoform CRF-R2 of Corticotropin-releasing factor receptor 1

Chain A:
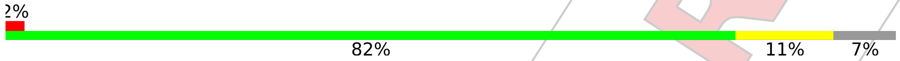

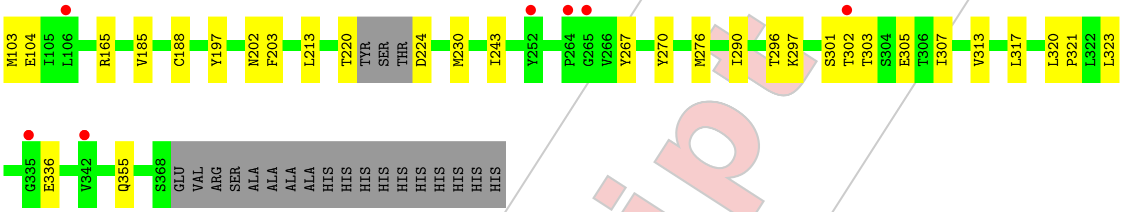

- Molecule 2: Endolysin

Chain B:
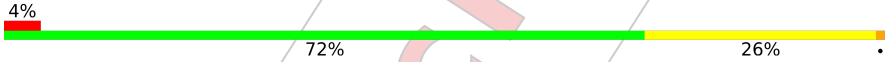

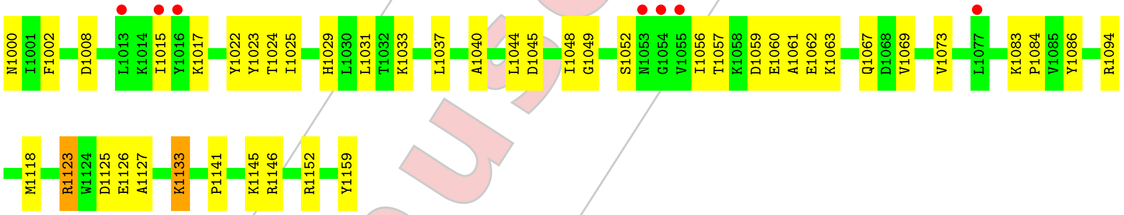

For Manuscript Review

4 Data and refinement statistics (i)

Property	Value	Source
Space group	C 1 2 1	Depositor
Cell constants<br>a, b, c, α, β, γ	95.66Å 70.65Å 86.75Å<br>90.00° 97.82° 90.00°	Depositor
Resolution (Å)	56.64 – 2.20<br>56.64 – 2.20	Depositor<br>EDS
% Data completeness<br>(in resolution range)	99.9 (56.64-2.20)<br>99.9 (56.64-2.20)	Depositor<br>EDS
R_{merge}	(Not available)	Depositor
R_{sym}	(Not available)	Depositor
$< I/\sigma(I) >$ [1]	1.53 (at 2.20Å)	Xtriage
Refinement program	PHENIX 1.18.2_3874	Depositor
R, R_{free}	0.208 , 0.237<br>0.208 , 0.237	Depositor<br>DCC
R_{free} test set	839 reflections (2.87%)	wwPDB-VP
Wilson B-factor (Å^2)	55.4	Xtriage
Anisotropy	0.323	Xtriage
Bulk solvent k_{sol}(e/Å^3), B_{sol}(Å^2)	0.30 , 76.9	EDS
L-test for twinning[2]	$< \|L\| > = 0.47$, $< L^2 > = 0.30$	Xtriage
Estimated twinning fraction	No twinning to report.	Xtriage
F_o,F_c correlation	0.96	EDS
Total number of atoms	3568	wwPDB-VP
Average B, all atoms (Å^2)	72.0	wwPDB-VP

Xtriage's analysis on translational NCS is as follows: *The largest off-origin peak in the Patterson function is 5.39% of the height of the origin peak. No significant pseudotranslation is detected.*

[1]Intensities estimated from amplitudes.

[2]Theoretical values of $< |L| >$, $< L^2 >$ for acentric reflections are 0.5, 0.333 respectively for untwinned datasets, and 0.375, 0.2 for perfectly twinned datasets.

For Manuscript Review

5 Model quality (i)

5.1 Standard geometry (i)

Bond lengths and bond angles in the following residue types are not validated in this section: OLA, 0JS

The Z score for a bond length (or angle) is the number of standard deviations the observed value is removed from the expected value. A bond length (or angle) with $|Z| > 5$ is considered an outlier worth inspection. RMSZ is the root-mean-square of all Z scores of the bond lengths (or angles).

Mol	Chain	Bond lengths		Bond angles	
		RMSZ	#|Z| >5	RMSZ	#|Z| >5
1	A	0.50	0/2116	0.57	0/2894
2	B	0.49	0/1251	0.63	2/1696 (0.1%)
All	All	0.50	0/3367	0.59	2/4590 (0.0%)

There are no bond length outliers.

All (2) bond angle outliers are listed below:

Mol	Chain	Res	Type	Atoms	Z	Observed(^{o})	Ideal(^{o})
2	B	1123	ARG	NE-CZ-NH1	-6.07	117.26	120.30
2	B	1123	ARG	CG-CD-NE	-5.30	100.66	111.80

There are no chirality outliers.

There are no planarity outliers.

5.2 Too-close contacts (i)

In the following table, the Non-H and H(model) columns list the number of non-hydrogen atoms and hydrogen atoms in the chain respectively. The H(added) column lists the number of hydrogen atoms added and optimized by MolProbity. The Clashes column lists the number of clashes within the asymmetric unit, whereas Symm-Clashes lists symmetry-related clashes.

Mol	Chain	Non-H	H(model)	H(added)	Clashes	Symm-Clashes
1	A	2056	0	2014	31	0
2	B	1231	0	1201	54	0
3	A	85	135	119	0	0
4	A	31	30	0	0	0
All	All	3403	165	3334	77	0

The all-atom clashscore is defined as the number of clashes found per 1000 atoms (including

For Manuscript Review

hydrogen atoms). The all-atom clashscore for this structure is 11.

All (77) close contacts within the same asymmetric unit are listed below, sorted by their clash magnitude.

Atom-1	Atom-2	Interatomic distance (Å)	Clash overlap (Å)
2:B:1015:ILE:HD11	2:B:1044:LEU:HD22	1.25	1.08
1:A:224:ASP:N	2:B:1159:TYR:O	1.95	0.99
2:B:1123:ARG:NH2	2:B:1126:GLU:OE1	1.96	0.96
1:A:220:THR:O	2:B:1000:ASN:N	2.03	0.91
2:B:1045:ASP:HA	2:B:1048:ILE:HG22	1.56	0.86
2:B:1125:ASP:OD1	2:B:1152:ARG:NH2	2.09	0.86
2:B:1133:LYS:HD2	2:B:1133:LYS:O	1.78	0.84
1:A:303:THR:O	1:A:307:ILE:HD12	1.80	0.82
2:B:1045:ASP:OD2	2:B:1052:SER:HA	1.80	0.81
2:B:1037:LEU:HD23	2:B:1037:LEU:O	1.82	0.79
1:A:103:MET:HG3	1:A:104:GLU:H	1.51	0.75
2:B:1023:TYR:CD2	2:B:1040:ALA:HB2	2.27	0.69
2:B:1045:ASP:HA	2:B:1048:ILE:CG2	2.25	0.67
2:B:1118:MET:HB3	2:B:1127:ALA:HB2	1.77	0.66
2:B:1152:ARG:HH21	2:B:1152:ARG:HG3	1.62	0.64
2:B:1141:PRO:O	2:B:1145:LYS:HD3	1.97	0.63
1:A:103:MET:HG3	1:A:104:GLU:N	2.12	0.63
2:B:1045:ASP:CG	2:B:1052:SER:HA	2.19	0.63
1:A:220:THR:O	2:B:1000:ASN:CA	2.47	0.62
1:A:185:VAL:HG13	1:A:188:CYS:HB2	1.81	0.61
2:B:1057:THR:HB	2:B:1060:GLU:H	1.65	0.61
2:B:1015:ILE:HG13	2:B:1025:ILE:HD12	1.83	0.61
2:B:1057:THR:CG2	2:B:1059:ASP:HB2	2.31	0.61
2:B:1017:LYS:HA	2:B:1022:TYR:O	2.01	0.60
2:B:1083:LYS:HB3	2:B:1084:PRO:HD3	1.81	0.60
1:A:220:THR:CA	2:B:1000:ASN:N	2.65	0.60
2:B:1022:TYR:CD2	2:B:1033:LYS:HA	2.37	0.60
2:B:1123:ARG:CZ	2:B:1126:GLU:OE1	2.50	0.58
1:A:320:LEU:HB3	1:A:321:PRO:HD3	1.86	0.57
2:B:1118:MET:CB	2:B:1127:ALA:HB2	2.34	0.57
1:A:313:VAL:O	1:A:317:LEU:HG	2.04	0.56
2:B:1045:ASP:O	2:B:1049:GLY:N	2.33	0.56
2:B:1083:LYS:HB3	2:B:1084:PRO:CD	2.36	0.56
2:B:1031:LEU:HD21	2:B:1044:LEU:HD13	1.90	0.53
2:B:1057:THR:HG22	2:B:1059:ASP:H	1.74	0.52
1:A:185:VAL:CG1	1:A:188:CYS:HB2	2.39	0.52
2:B:1057:THR:HG21	2:B:1059:ASP:HB2	1.91	0.52
2:B:1118:MET:HE3	2:B:1123:ARG:HH12	1.75	0.52

Continued on next page...

For Manuscript Review

Continued from previous page...

Atom-1	Atom-2	Interatomic distance (Å)	Clash overlap (Å)
1:A:220:THR:C	2:B:1000:ASN:N	2.62	0.51
2:B:1133:LYS:O	2:B:1133:LYS:CD	2.55	0.51
1:A:267:TYR:HB3	1:A:270:TYR:CD2	2.44	0.51
1:A:336:GLU:OE1	1:A:336:GLU:N	2.39	0.50
2:B:1008:ASP:HB3	2:B:1146:ARG:HH21	1.75	0.50
2:B:1002:PHE:CD1	2:B:1062:GLU:HG3	2.46	0.50
1:A:103:MET:CG	1:A:104:GLU:N	2.72	0.50
1:A:203:PHE:CE1	1:A:276:MET:HE2	2.47	0.50
1:A:220:THR:HA	2:B:1000:ASN:N	2.25	0.49
2:B:1024:THR:HG22	2:B:1025:ILE:N	2.27	0.49
2:B:1037:LEU:HD23	2:B:1037:LEU:C	2.32	0.49
1:A:296:THR:OG1	1:A:297:LYS:N	2.44	0.49
2:B:1123:ARG:NH1	2:B:1126:GLU:OE1	2.46	0.48
2:B:1056:ILE:HD12	2:B:1061:ALA:N	2.28	0.48
1:A:230:MET:HA	1:A:230:MET:HE2	1.96	0.48
1:A:224:ASP:CA	2:B:1159:TYR:O	2.61	0.47
2:B:1023:TYR:HD2	2:B:1040:ALA:HB2	1.79	0.46
2:B:1069:VAL:O	2:B:1073:VAL:HG23	2.16	0.46
2:B:1031:LEU:HD21	2:B:1044:LEU:CD1	2.47	0.45
2:B:1118:MET:HE3	2:B:1123:ARG:NH1	2.31	0.45
2:B:1024:THR:HG23	2:B:1029:HIS:C	2.37	0.45
1:A:203:PHE:CE1	1:A:276:MET:CE	3.00	0.44
2:B:1063:LYS:O	2:B:1067:GLN:HG3	2.16	0.44
1:A:165:ARG:HD2	1:A:202:ASN:OD1	2.16	0.44
1:A:323:LEU:HD22	1:A:355:GLN:HG3	2.00	0.44
1:A:213:LEU:HD23	1:A:290:ILE:HD13	2.00	0.43
2:B:1057:THR:HB	2:B:1060:GLU:N	2.32	0.43
1:A:185:VAL:O	1:A:188:CYS:HB3	2.19	0.43
2:B:1057:THR:H	2:B:1060:GLU:CB	2.31	0.43
1:A:224:ASP:N	2:B:1159:TYR:C	2.69	0.43
1:A:303:THR:HG21	1:A:305:GLU:OE2	2.18	0.43
1:A:197:TYR:HA	1:A:243:ILE:HG13	2.01	0.43
1:A:307:ILE:HD12	1:A:307:ILE:H	1.83	0.43
2:B:1152:ARG:NH2	2:B:1152:ARG:HG3	2.32	0.42
2:B:1045:ASP:OD2	2:B:1052:SER:CA	2.61	0.42
1:A:230:MET:HA	1:A:230:MET:CE	2.50	0.42
1:A:301:SER:O	1:A:302:THR:HG23	2.21	0.41
2:B:1045:ASP:OD1	2:B:1052:SER:HA	2.20	0.41
2:B:1086:TYR:CZ	2:B:1094:ARG:HD3	2.56	0.40

There are no symmetry-related clashes.

For Manuscript Review

5.3 Torsion angles (i)

5.3.1 Protein backbone (i)

In the following table, the Percentiles column shows the percent Ramachandran outliers of the chain as a percentile score with respect to all X-ray entries followed by that with respect to entries of similar resolution.

The Analysed column shows the number of residues for which the backbone conformation was analysed, and the total number of residues.

Mol	Chain	Analysed	Favoured	Allowed	Outliers	Percentiles	
1	A	259/284 (91%)	252 (97%)	7 (3%)	0	100	100
2	B	158/160 (99%)	153 (97%)	5 (3%)	0	100	100
All	All	417/444 (94%)	405 (97%)	12 (3%)	0	100	100

There are no Ramachandran outliers to report.

5.3.2 Protein sidechains (i)

In the following table, the Percentiles column shows the percent sidechain outliers of the chain as a percentile score with respect to all X-ray entries followed by that with respect to entries of similar resolution.

The Analysed column shows the number of residues for which the sidechain conformation was analysed, and the total number of residues.

Mol	Chain	Analysed	Rotameric	Outliers	Percentiles	
1	A	210/243 (86%)	210 (100%)	0	100	100
2	B	125/134 (93%)	124 (99%)	1 (1%)	81	90
All	All	335/377 (89%)	334 (100%)	1 (0%)	92	97

All (1) residues with a non-rotameric sidechain are listed below:

Mol	Chain	Res	Type
2	B	1133	LYS

Sometimes sidechains can be flipped to improve hydrogen bonding and reduce clashes. All (2) such sidechains are listed below:

Mol	Chain	Res	Type
1	A	182	GLN
1	A	289	ASN

For Manuscript Review

5.3.3 RNA (i)

There are no RNA molecules in this entry.

5.4 Non-standard residues in protein, DNA, RNA chains (i)

There are no non-standard protein/DNA/RNA residues in this entry.

5.5 Carbohydrates (i)

There are no monosaccharides in this entry.

5.6 Ligand geometry (i)

7 ligands are modelled in this entry.

In the following table, the Counts columns list the number of bonds (or angles) for which Mogul statistics could be retrieved, the number of bonds (or angles) that are observed in the model and the number of bonds (or angles) that are defined in the Chemical Component Dictionary. The Link column lists molecule types, if any, to which the group is linked. The Z score for a bond length (or angle) is the number of standard deviations the observed value is removed from the expected value. A bond length (or angle) with $|Z| > 2$ is considered an outlier worth inspection. RMSZ is the root-mean-square of all Z scores of the bond lengths (or angles).

Mol	Type	Chain	Res	Link	Bond lengths			Bond angles		
					Counts	RMSZ	$\#\|Z\| > 2$	Counts	RMSZ	$\#\|Z\| > 2$
3	OLA	A	704	-	14,14,19	0.67	0	14,14,19	1.20	1 (7%)
4	0JS	A	706	-	29,34,34	2.29	6 (20%)	35,49,49	2.03	9 (25%)
3	OLA	A	705	-	19,19,19	0.65	0	19,19,19	1.09	1 (5%)
3	OLA	A	703	-	19,19,19	0.55	0	19,19,19	1.08	1 (5%)
3	OLA	A	702	-	19,19,19	0.66	0	19,19,19	0.87	1 (5%)

In the following table, the Chirals column lists the number of chiral outliers, the number of chiral centers analysed, the number of these observed in the model and the number defined in the Chemical Component Dictionary. Similar counts are reported in the Torsion and Rings columns. '-' means no outliers of that kind were identified.

Mol	Type	Chain	Res	Link	Chirals	Torsions	Rings
3	OLA	A	704	-	-	8/12/12/17	-
4	0JS	A	706	-	-	6/20/22/22	0/4/4/4
3	OLA	A	705	-	-	7/17/17/17	-

Continued on next page...

For Manuscript Review

Continued from previous page...

Mol	Type	Chain	Res	Link	Chirals	Torsions	Rings
3	OLA	A	703	-	-	5/17/17/17	-
3	OLA	A	702	-	-	8/17/17/17	-

All (6) bond length outliers are listed below:

Mol	Chain	Res	Type	Atoms	Z	Observed(Å)	Ideal(Å)
4	A	706	0JS	C10-N05	7.85	1.46	1.35
4	A	706	0JS	C20-C15	5.91	1.54	1.50
4	A	706	0JS	BR2-C25	3.68	1.97	1.90
4	A	706	0JS	O29-C28	3.27	1.42	1.37
4	A	706	0JS	C19-C16	2.62	1.54	1.50
4	A	706	0JS	O22-C21	2.21	1.40	1.37

All (13) bond angle outliers are listed below:

Mol	Chain	Res	Type	Atoms	Z	Observed(^{o})	Ideal(^{o})
4	A	706	0JS	C10-N11-C12	7.38	119.94	114.75
4	A	706	0JS	C12-N13-C14	4.93	119.53	115.52
4	A	706	0JS	N13-C12-N11	-3.21	119.87	125.73
4	A	706	0JS	C19-C16-N17	3.13	126.42	119.75
4	A	706	0JS	C07-C06-N05	-3.03	109.05	114.02
3	A	704	OLA	C3-C2-C1	-2.77	107.49	114.47
4	A	706	0JS	C31-C12-N13	2.39	120.89	117.16
3	A	703	OLA	C3-C2-C1	-2.35	108.55	114.47
4	A	706	0JS	C27-C28-C20	-2.23	118.66	121.74
4	A	706	0JS	C28-C27-C25	2.15	121.18	118.68
3	A	705	OLA	C3-C2-C1	-2.12	109.14	114.47
4	A	706	0JS	C08-C07-C06	-2.05	110.92	120.30
3	A	702	OLA	O2-C1-C2	2.02	120.52	114.03

There are no chirality outliers.

All (34) torsion outliers are listed below:

Mol	Chain	Res	Type	Atoms
4	A	706	0JS	N11-C10-N05-C04
4	A	706	0JS	N11-C10-N05-C06
4	A	706	0JS	N18-C10-N05-C04
4	A	706	0JS	N18-C10-N05-C06
4	A	706	0JS	C01-C02-C03-C04
3	A	702	OLA	C1-C2-C3-C4
3	A	704	OLA	C1-C2-C3-C4

Continued on next page...

For Manuscript Review

Continued from previous page...

Mol	Chain	Res	Type	Atoms
3	A	702	OLA	C12-C13-C14-C15
3	A	705	OLA	C7-C8-C9-C10
3	A	705	OLA	C5-C6-C7-C8
3	A	703	OLA	C14-C15-C16-C17
3	A	703	OLA	C7-C8-C9-C10
3	A	705	OLA	C12-C13-C14-C15
3	A	702	OLA	C6-C7-C8-C9
3	A	704	OLA	C2-C3-C4-C5
4	A	706	0JS	N05-C06-C07-C09
3	A	702	OLA	C15-C16-C17-C18
3	A	705	OLA	C11-C12-C13-C14
3	A	704	OLA	C5-C6-C7-C8
3	A	705	OLA	C9-C10-C11-C12
3	A	705	OLA	O1-C1-C2-C3
3	A	703	OLA	C5-C6-C7-C8
3	A	704	OLA	C9-C10-C11-C12
3	A	704	OLA	C4-C5-C6-C7
3	A	705	OLA	O2-C1-C2-C3
3	A	702	OLA	C3-C4-C5-C6
3	A	703	OLA	C3-C4-C5-C6
3	A	702	OLA	C7-C8-C9-C10
3	A	704	OLA	C7-C8-C9-C10
3	A	703	OLA	C6-C7-C8-C9
3	A	702	OLA	C9-C10-C11-C12
3	A	704	OLA	O1-C1-C2-C3
3	A	704	OLA	O2-C1-C2-C3
3	A	702	OLA	C5-C6-C7-C8

There are no ring outliers.

No monomer is involved in short contacts.

The following is a two-dimensional graphical depiction of Mogul quality analysis of bond lengths, bond angles, torsion angles, and ring geometry for all instances of the Ligand of Interest. In addition, ligands with molecular weight > 250 and outliers as shown on the validation Tables will also be included. For torsion angles, if less then 5% of the Mogul distribution of torsion angles is within 10 degrees of the torsion angle in question, then that torsion angle is considered an outlier. Any bond that is central to one or more torsion angles identified as an outlier by Mogul will be highlighted in the graph. For rings, the root-mean-square deviation (RMSD) between the ring in question and similar rings identified by Mogul is calculated over all ring torsion angles. If the average RMSD is greater than 60 degrees and the minimal RMSD between the ring in question and any Mogul-identified rings is also greater than 60 degrees, then that ring is considered an outlier. The outliers are highlighted in purple. The color gray indicates Mogul did not find sufficient equivalents in the CSD to analyse the geometry.

For Manuscript Review

Ligand OLA A 704

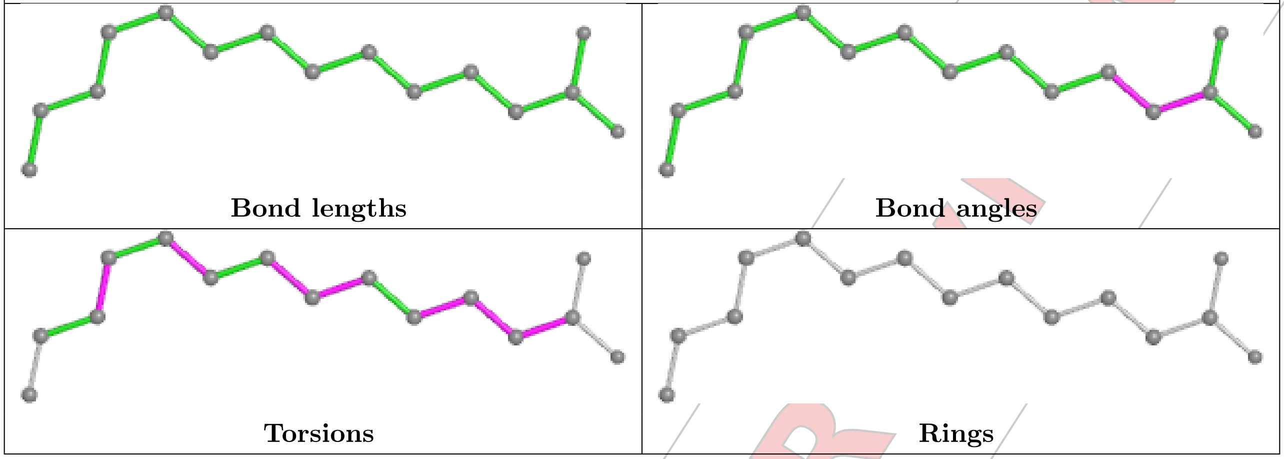

Ligand 0JS A 706

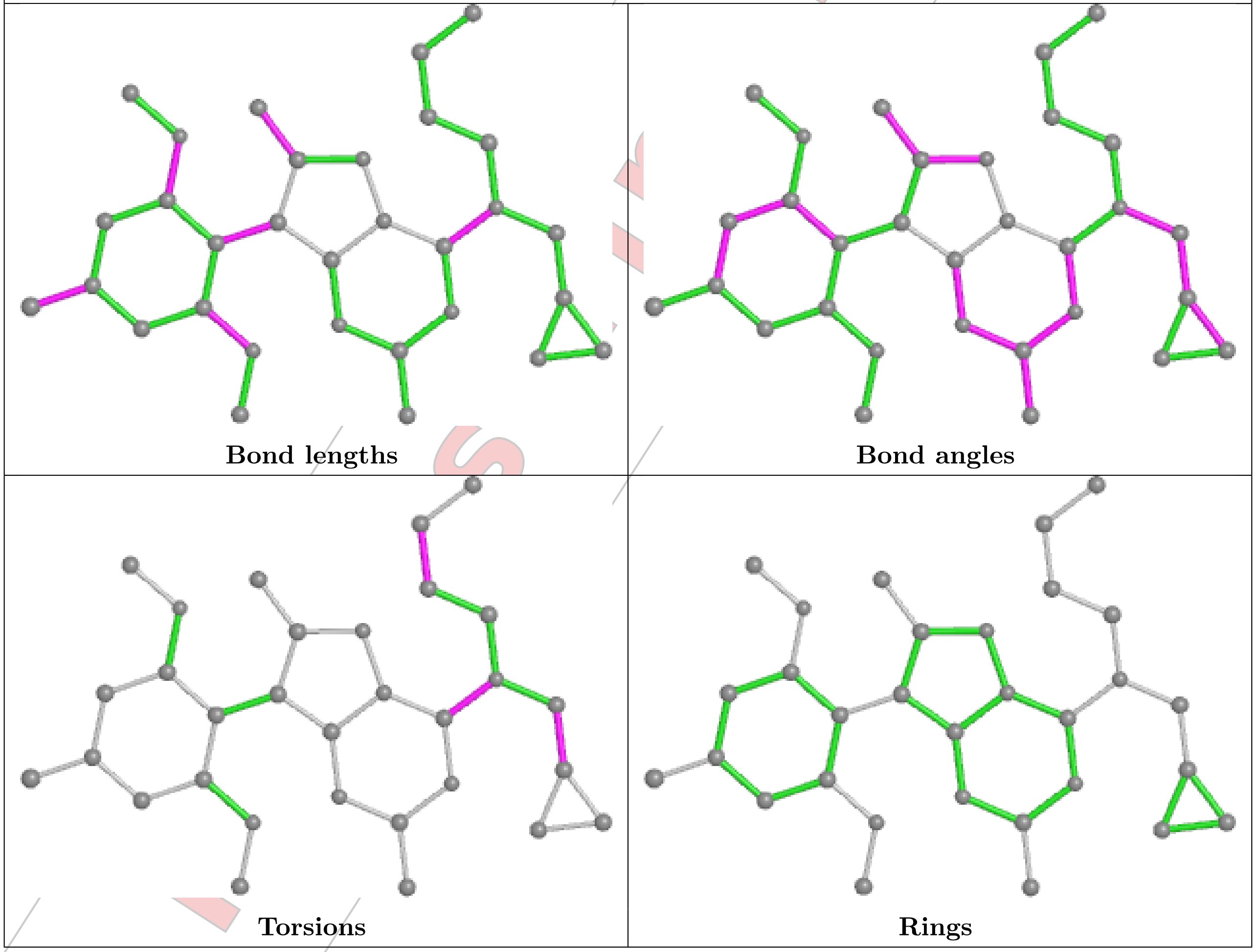

For Review

Ligand OLA A 705

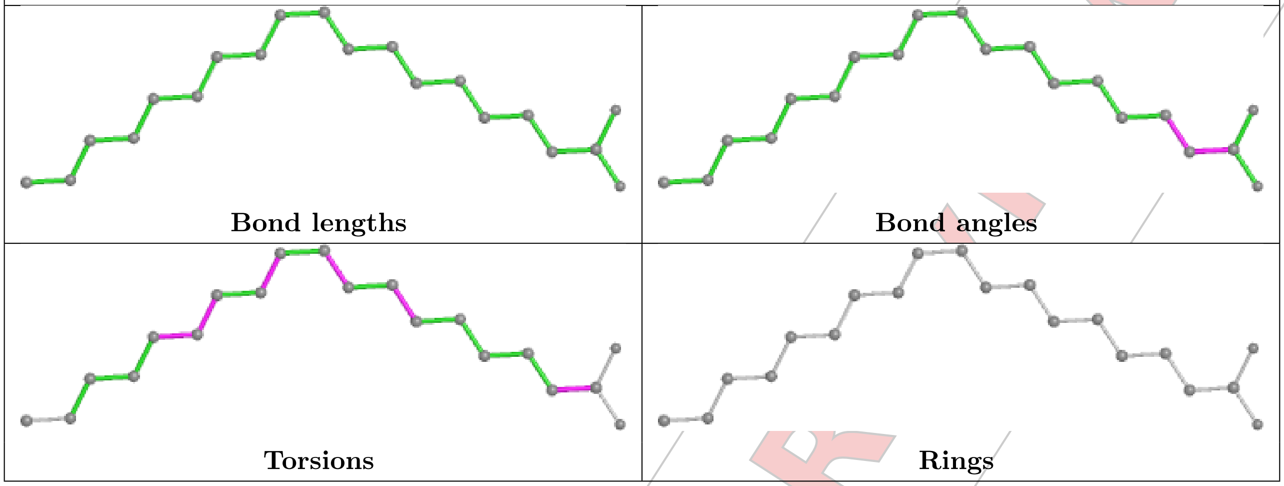

Ligand OLA A 703

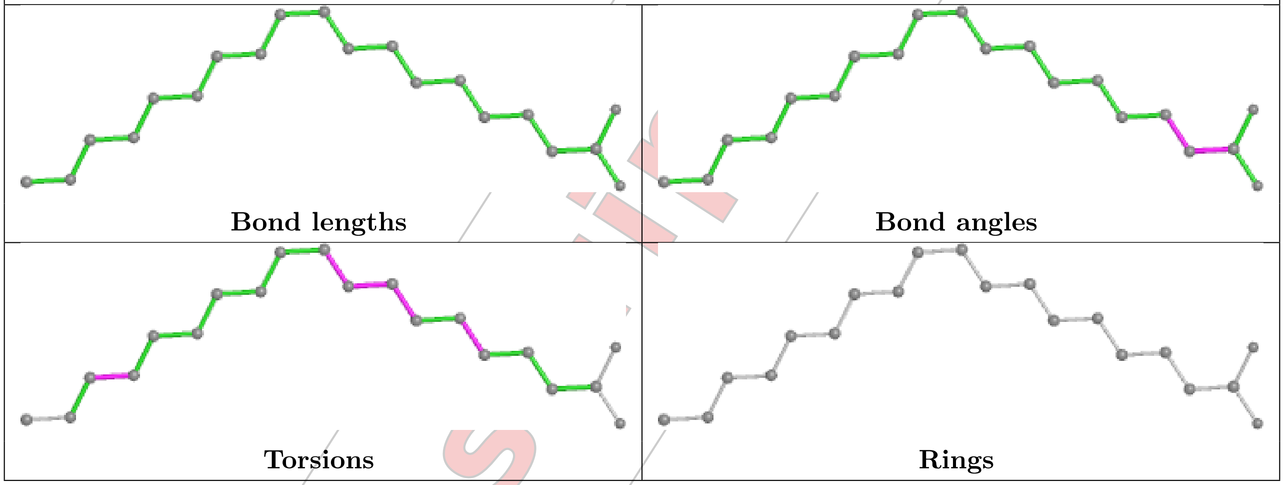

Ligand OLA A 702

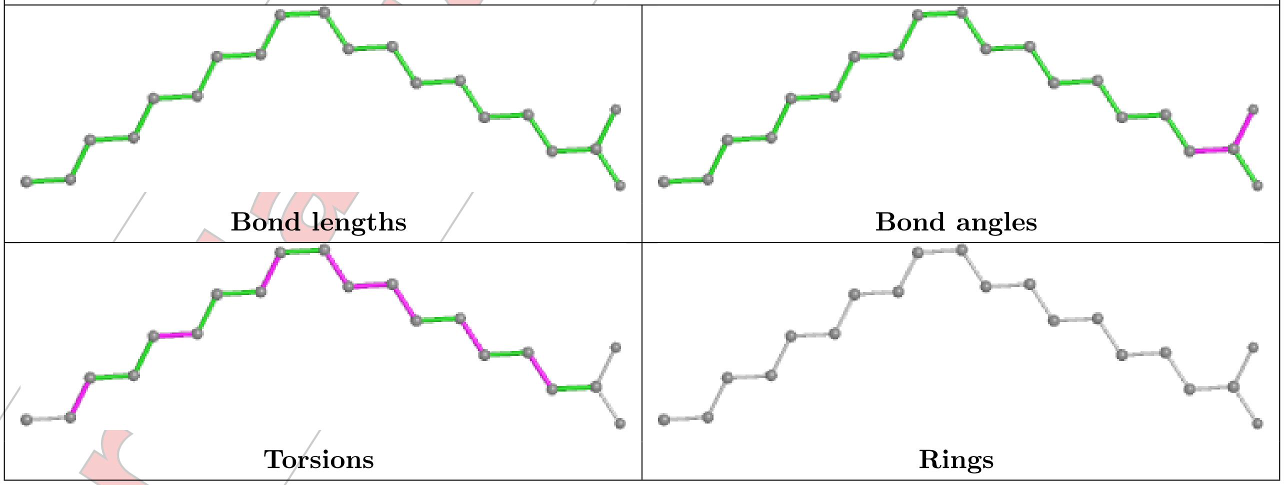

5.7 Other polymers ⓘ

There are no such residues in this entry.

5.8 Polymer linkage issues (i)

There are no chain breaks in this entry.

For Manuscript Review

6 Fit of model and data (i)

6.1 Protein, DNA and RNA chains (i)

In the following table, the column labelled '#RSRZ> 2' contains the number (and percentage) of RSRZ outliers, followed by percent RSRZ outliers for the chain as percentile scores relative to all X-ray entries and entries of similar resolution. The OWAB column contains the minimum, median, 95th percentile and maximum values of the occupancy-weighted average B-factor per residue. The column labelled 'Q< 0.9' lists the number of (and percentage) of residues with an average occupancy less than 0.9.

Mol	Chain	Analysed	<RSRZ>	#RSRZ>2			OWAB(Å^2)	Q<0.9
1	A	263/284 (92%)	0.10	7 (2%)	54	52	43, 64, 114, 150	0
2	B	160/160 (100%)	-0.05	7 (4%)	34	32	47, 66, 129, 150	0
All	All	423/444 (95%)	0.04	14 (3%)	46	44	43, 65, 120, 150	0

All (14) RSRZ outliers are listed below:

Mol	Chain	Res	Type	RSRZ
1	A	264	PRO	6.2
1	A	106	LEU	4.4
1	A	252	TYR	3.9
1	A	265	GLY	3.6
2	B	1015	ILE	3.2
2	B	1016	TYR	3.2
1	A	342	VAL	3.2
1	A	335	GLY	3.0
2	B	1013	LEU	2.8
2	B	1053	ASN	2.6
2	B	1054	GLY	2.5
1	A	302	THR	2.2
2	B	1077	LEU	2.1
2	B	1055	VAL	2.0

6.2 Non-standard residues in protein, DNA, RNA chains (i)

There are no non-standard protein/DNA/RNA residues in this entry.

6.3 Carbohydrates (i)

There are no monosaccharides in this entry.

For Manuscript Review

6.4 Ligands (i)

In the following table, the Atoms column lists the number of modelled atoms in the group and the number defined in the chemical component dictionary. The B-factors column lists the minimum, median, 95th percentile and maximum values of B factors of atoms in the group. The column labelled 'Q< 0.9' lists the number of atoms with occupancy less than 0.9.

Mol	Type	Chain	Res	Atoms	RSCC	RSR	B-factors($Å^2$)	Q<0.9
3	OLA	A	702	20/20	0.75	0.32	70,96,118,123	0
3	OLA	A	705	20/20	0.76	0.25	71,88,100,105	0
3	OLA	A	703	20/20	0.78	0.32	80,111,180,187	0
3	OLA	A	704	15/20	0.83	0.33	72,91,109,110	0
3	OLA	A	701[A]	10/20	0.89	0.20	78,94,105,105	1
3	OLA	A	701[B]	10/20	0.89	0.20	78,94,105,105	1
4	0JS	A	706	31/31	0.98	0.15	46,58,79,81	0

The following is a graphical depiction of the model fit to experimental electron density of all instances of the Ligand of Interest. In addition, ligands with molecular weight > 250 and outliers as shown on the geometry validation Tables will also be included. Each fit is shown from different orientation to approximate a three-dimensional view.

Electron density around OLA A 702:

$2mF_o$-DF_c (at 0.7 rmsd) in gray
mF_o-DF_c (at 3 rmsd) in purple (negative) and green (positive)

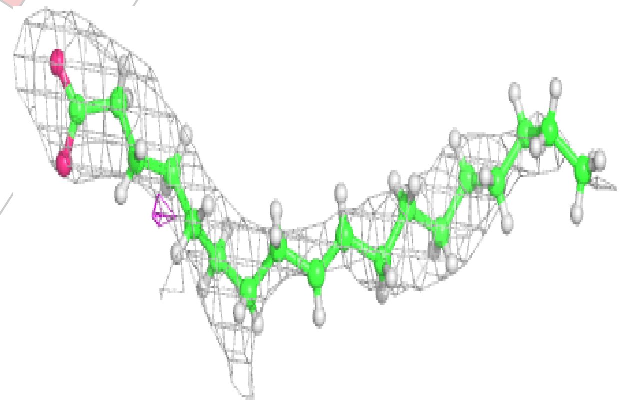

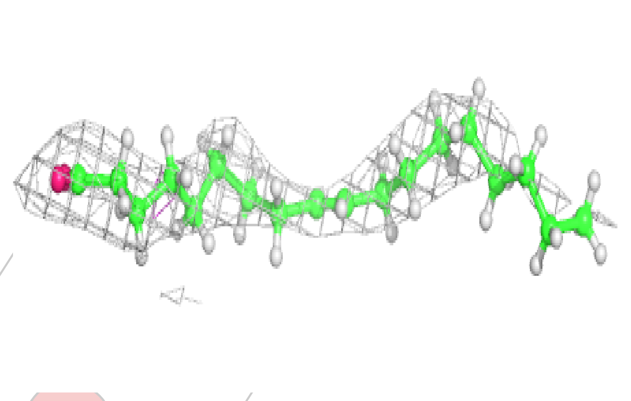

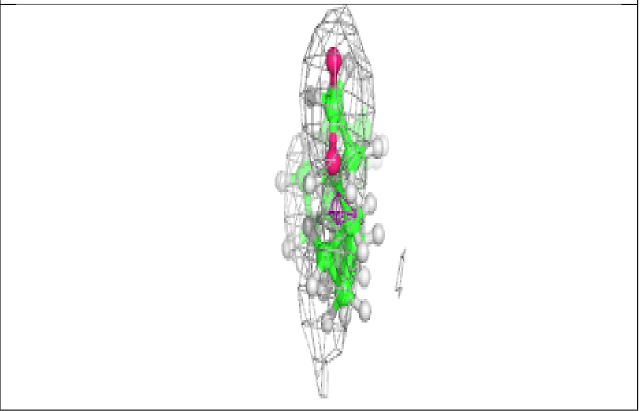

For Manuscript Review

Electron density around OLA A 705:

$2mF_o$-DF_c (at 0.7 rmsd) in gray
mF_o-DF_c (at 3 rmsd) in purple (negative)
and green (positive)

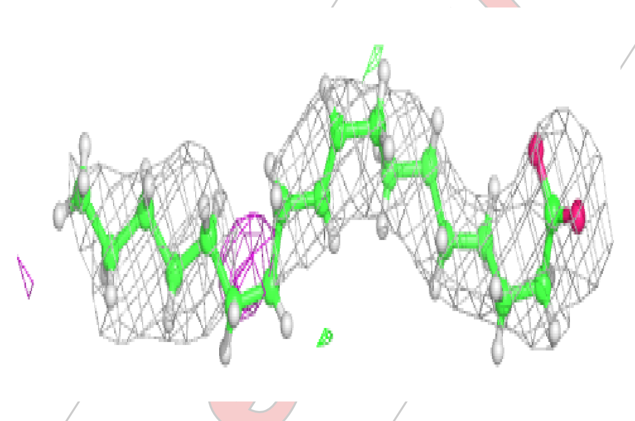

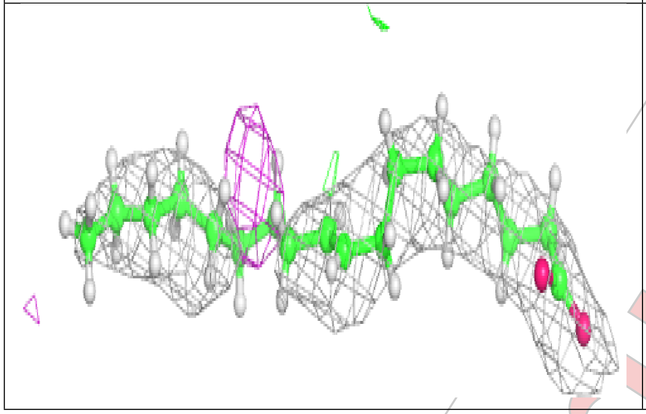

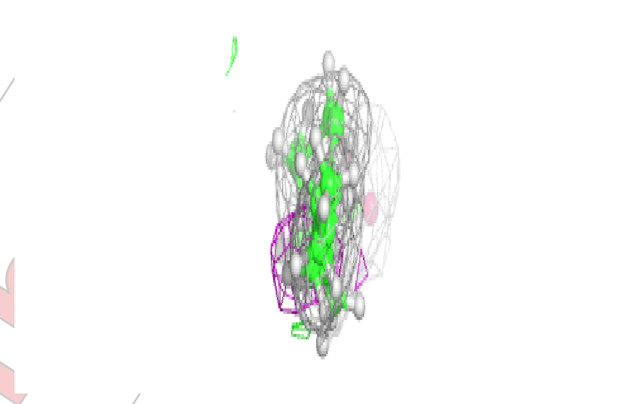

Electron density around OLA A 703:

$2mF_o$-DF_c (at 0.7 rmsd) in gray
mF_o-DF_c (at 3 rmsd) in purple (negative)
and green (positive)

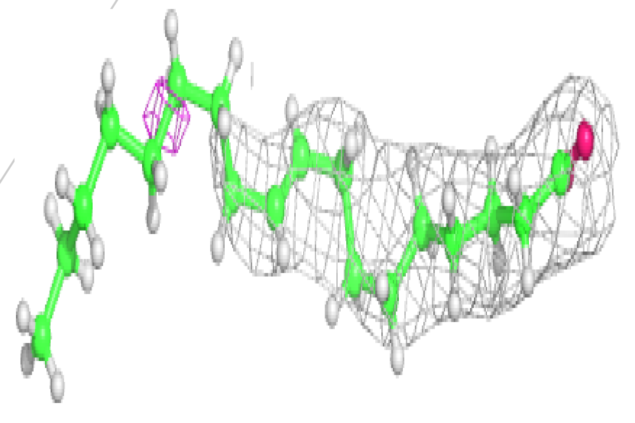

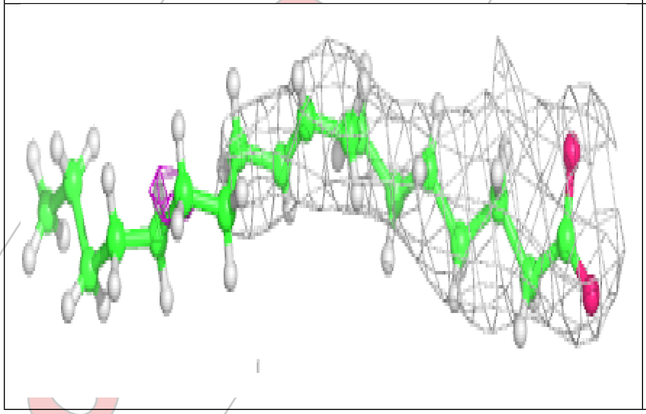

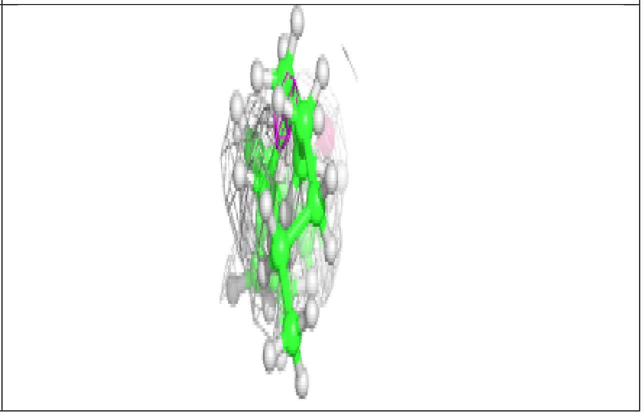

Electron density around OLA A 704:

$2mF_o$-DF_c (at 0.7 rmsd) in gray
mF_o-DF_c (at 3 rmsd) in purple (negative)
and green (positive)

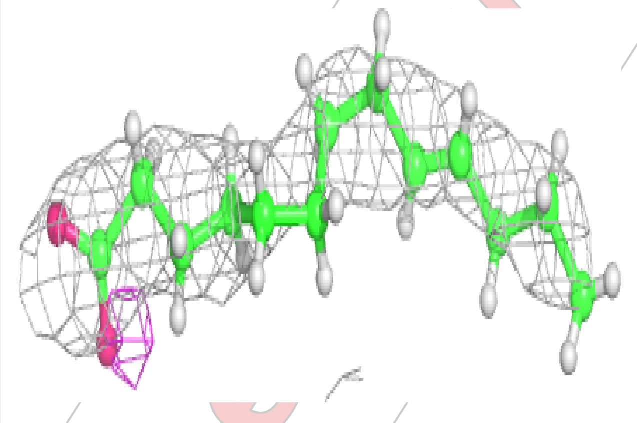

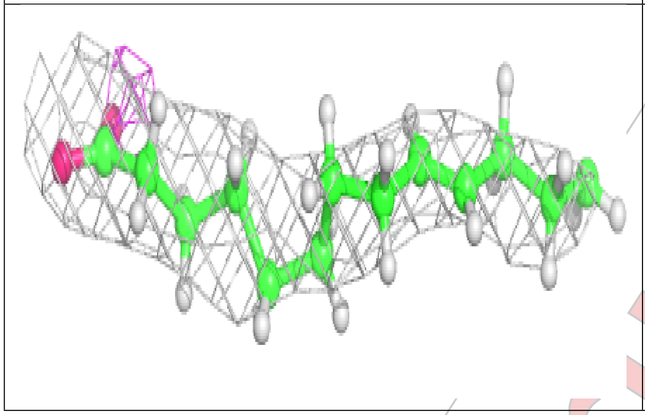

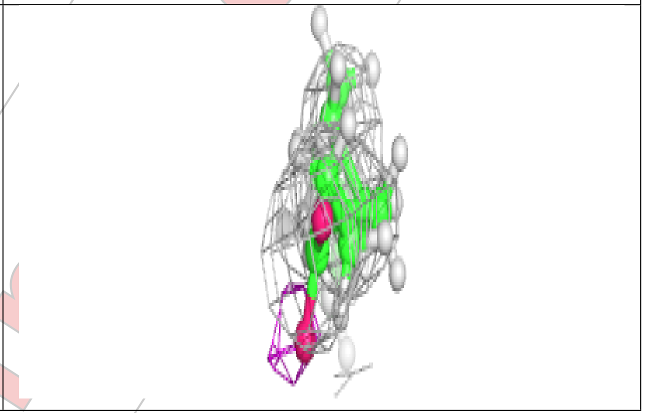

Electron density around 0JS A 706:

$2mF_o$-DF_c (at 0.7 rmsd) in gray
mF_o-DF_c (at 3 rmsd) in purple (negative)
and green (positive)

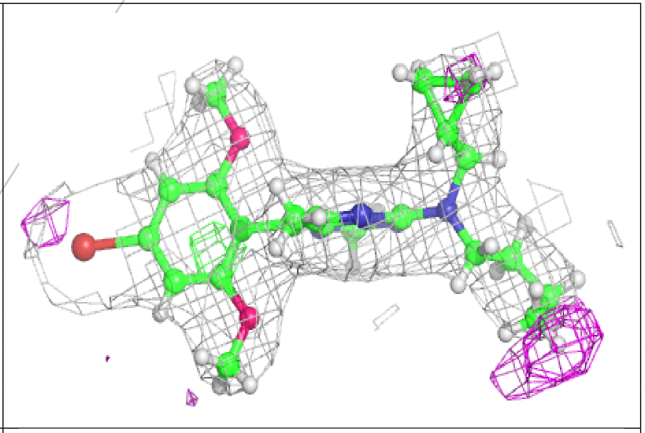

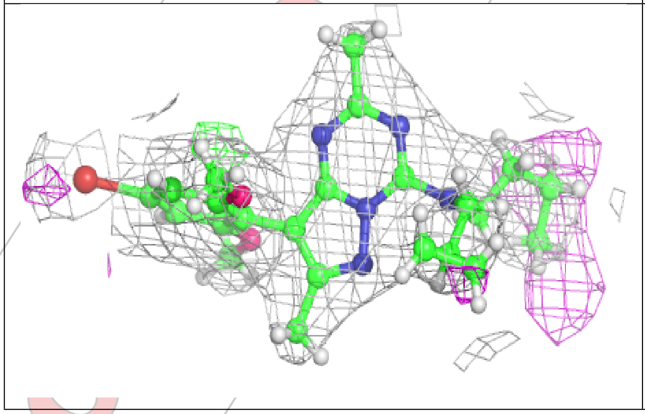

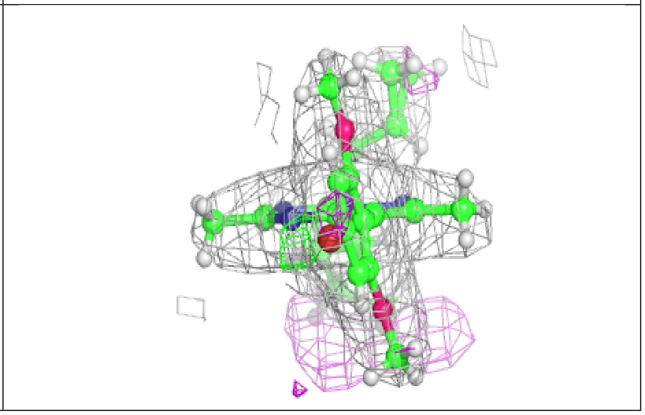

For Manuscript Review

6.5 Other polymers (i)

There are no such residues in this entry.

For Manuscript Review
